# Transition metals and oxidation reactions trigger stargate opening during the initial stages of the replicative cycle of the giant Tupanvirus

**DOI:** 10.1128/mbio.02192-24

**Published:** 2024-09-26

**Authors:** Juliana R. Cortines, Charles M. Bridges, Sundharraman Subramanian, Jason R. Schrad, Glauber R. S. Araújo, Gabriel Henrique Pereira Nunes, Juliana dos Santos Oliveira, Victor Alejandro Essus, Jônatas S. Abrahão, Simon White, Kristin N. Parent, Carolyn Teschke

**Affiliations:** 1 Departamento de Virologia, Instituto de Microbiologia Paulo de Góes, Universidade Federal do Rio de Janeiro, Rio de Janeiro, Brazil; 2 Department of Molecular and Cell Biology, University of Connecticut, Storrs, Connecticut, USA; 3 Department of Biochemistry and Molecular Biology, Michigan State University, East Lansing, Michigan, USA; 4 Laboratório de Biofísica de Fungos, Instituto de Biofísica Carlos Chagas Filho, Universidade Federal do Rio de Janeiro, Rio de Janeiro, Brazil; 5 Department of Microbiology, Federal University of Minas Gerais, Institute of Biological Sciences, Belo Horizonte, Minas Gerais, Brazil; 6 Department of Chemistry, University of Connecticut, Storrs, Connecticut, USA; University of Pittsburgh, Pittsburgh, Pennsylvania, USA

**Keywords:** Tupanvirus, iron, copper, ROS, nutritional immunity

## Abstract

Tupanviruses, members of the family *Mimiviridae*, infect phagocytic cells. Particle uncoating begins inside the phagosome, with capsid opening via the stargate. The mechanism through which this opening takes place is unknown. Once phagocytized, metal ion flux control and ROS are induced to inactivate foreign particles, including viruses. Here, we studied the effect of iron ions, copper ions, and H_2_O_2_ on Tupanvirus particles. Such treatments induced stargate opening *in vitro*, as observed by different microscopy techniques. Metal-treated viruses were found to be non-infectious, leading to the hypothesis that stargate opening likely resulted in the release of the viral seed, which is required for infection initiation. To the best of our knowledge, this is the first description of a giant virus capsid morphological change induced by transition metals and H_2_O_2_, which may be important to describe new virulence factors and capsid uncoating mechanisms.

## INTRODUCTION

Tupanviruses (TPV) are members of the *Mimiviridae* family and are the largest viruses known to date. The capsids are ~450 nm in diameter and are covered by a dense layer of fibrils, except at a single fivefold vertex, where a star-like structure known as the stargate resides (1–4). They have a unique tail located opposite the stargate; this cylindric structure varies considerably in size, with >500 nm in length, and is also covered by fibrils (4, 5).

TPV infection is initiated when viral particles are phagocytized by amoebas. Then, the virus’ stargate opens inside the phagosome, resulting in the sequential release of the viral seed followed by the viral genome. The viral seed is composed of several vesicle-bound macromolecules believed to trigger the phagosomal egress of viral components and factors that induce the formation of the viral factory (3, 4, 6). The viral factory itself and its vicinity are the sites of viral transcription, translation, and particle assembly. Nearing 16 hours post-infection, the cytoplasm is filled with viral progeny, which leads to cell lysis and particle egress (1, 5, 7, 8). Proteomic data showed the presence of metal-binding proteins in the viral seed (3), leading to the hypothesis that metallic ions and phagosomal nutritional immunity could be involved in the initial TPV replication steps (9).

Phagosomes are one of the first lines of intracellular defense. They are built to be remarkably efficient in deactivation and digestion of the internalized contents, including pathogens. During the maturation of the digestive vesicle, many proteins are recruited to act in this process. The vacuolar ATPase (v-ATPase) translocates H^+^ ions into the phagosomal lumen, gradually lowering the internal pH to ≤5 (10). Concurrently, the NADPH oxidase complex is responsible for the production of reactive oxygen species (ROS) including hydrogen peroxide (H_2_O_2_) (11). Other proteins include digestive enzymes such as proteases, lipases, glycosylases, and DNases (10, 12). Another strategy applied for the elimination of the phagocytized particulate is nutritional immunity, which modulates the flux of some transition metals, such as iron, manganese, copper, and zinc (13).

Transition metals are required in trace amounts for numerous biological processes, mainly acting as cofactors to essential enzymes such as oxidoreductases, hydrolases, transferases, and isomerases (14). At high concentrations, many of these metals exhibit cellular toxicity. Nutritional immunity takes advantage of the toxic properties of copper and zinc ions, as their accumulation in the phagosome reacts with ROS, potentiating oxidative damage to the phagocytosed pathogen (9). Additionally, transporters such as natural resistance-associated macrophage proteins are responsible for the efflux of iron ions, which are required for the growth and virulence of some pathogens and can also result in pathogen killing (15).

To escape death from intoxication, some microorganisms can, through metallosensor proteins, increase the transcription of efflux pump genes responsible for exporting copper ions out of the microorganism. For example, *Mycobacterium tuberculosis* increases the transcription of a series of heavy metal efflux transporters (9), while other bacteria produce iron and manganese chelators (siderophores) or have copper efflux systems, as the Gram-negative *Legionella pneumophila*, which produces a siderophore known as legiobactin (9, 13, 16).

Metal ions are required during the biosynthesis of several viruses. They are involved in capsid stabilization and participate as co-factors for enzymes involved in viral genome replication and processing (17). Likewise, it is reasonable to assume metals play an important role in the replicative cycle of viruses infecting professional phagocytes, which exhibit nutritional immunity. These cells rely on the redox properties of metals as their forefront defense. Thus, any phagocytized parasite, including giant viruses (GVs), must escape or exploit these defenses.

The molecular mechanisms of mimivirus’ stargate opening are unknown. A previous study from our groups observed that the capsid of TPV is opened *in vitro* under extreme conditions: acidic pH (<4), high temperature (100°C), or high salinity (~4 M NaCl) (3). Though these harsh conditions lead to structural transitions that mimic those naturally occurring during Tupanvirus infection, high temperature and/or high salinity is not physiological during the natural replication cycle, suggesting other mechanisms are needed to fully open the virions *in vivo*. Therefore, we inquired whether phagosomal components including iron, copper, and H_2_O_2_ would influence the virus capsid structure.

## RESULTS

### Metal-binding associations with calcium, manganese, and nickel are enriched in proteins identified during early infection

Previous proteomic analysis of TPV viral seed provided evidence that metals are important during the initiation of infection due to the detection of mg709 and Cu-Zn superoxidedismutase, among others (3). To examine the putative role of divalent metal ions on viral structure and early replication stages, we used MeBiPred (18) an *in silico* tool to find predicted metal-binding interactions in the Tupanvirus proteome. Data analysis focused on proteins previously identified by mass spectrometry (MS) to be putatively located within the viral seed (3). Using a cutoff of 0.4, we found 592 putative metal-binding interactions covering approximately 74% (141 of 190) of the proteins previously identified by MS. Putative metal-binding proteins broadly fell into seven categories (Fig. S1A): hypothetical proteins (46.1%); DNA replication, transcription, and mRNA processing (17%); ORFans (11.3%); protein synthesis, modification and degradation (9.9%); viral structural proteins (5.7%); cell adhesion, signal transduction, and subcellular trafficking (4.3%); redox/radical scavenging proteins (3.5%); and hydrolase activity (2.1%). Specific metals involved in putative protein-binding interactions were distributed as follows: sodium (17%), calcium (13.8%), zinc (12.8%), magnesium (12.7%), potassium (12.5%), nickel (9.9%), manganese (6.6%) cobalt (6%), iron (6%), and copper (2.3%) (Fig. S1B).

Putative metal-binding proteins within assigned functional categories did not exhibit strict requirements for particular metal cofactors, with all metals represented in all categories, with the exception that proteins annotated with a hydrolase activity function did not appear to associate with cobalt, copper, iron, potassium, or manganese; and that capsid and other annotated structural proteins were not observed to interact with either copper or iron (Fig. S2).

**Fig 1:**
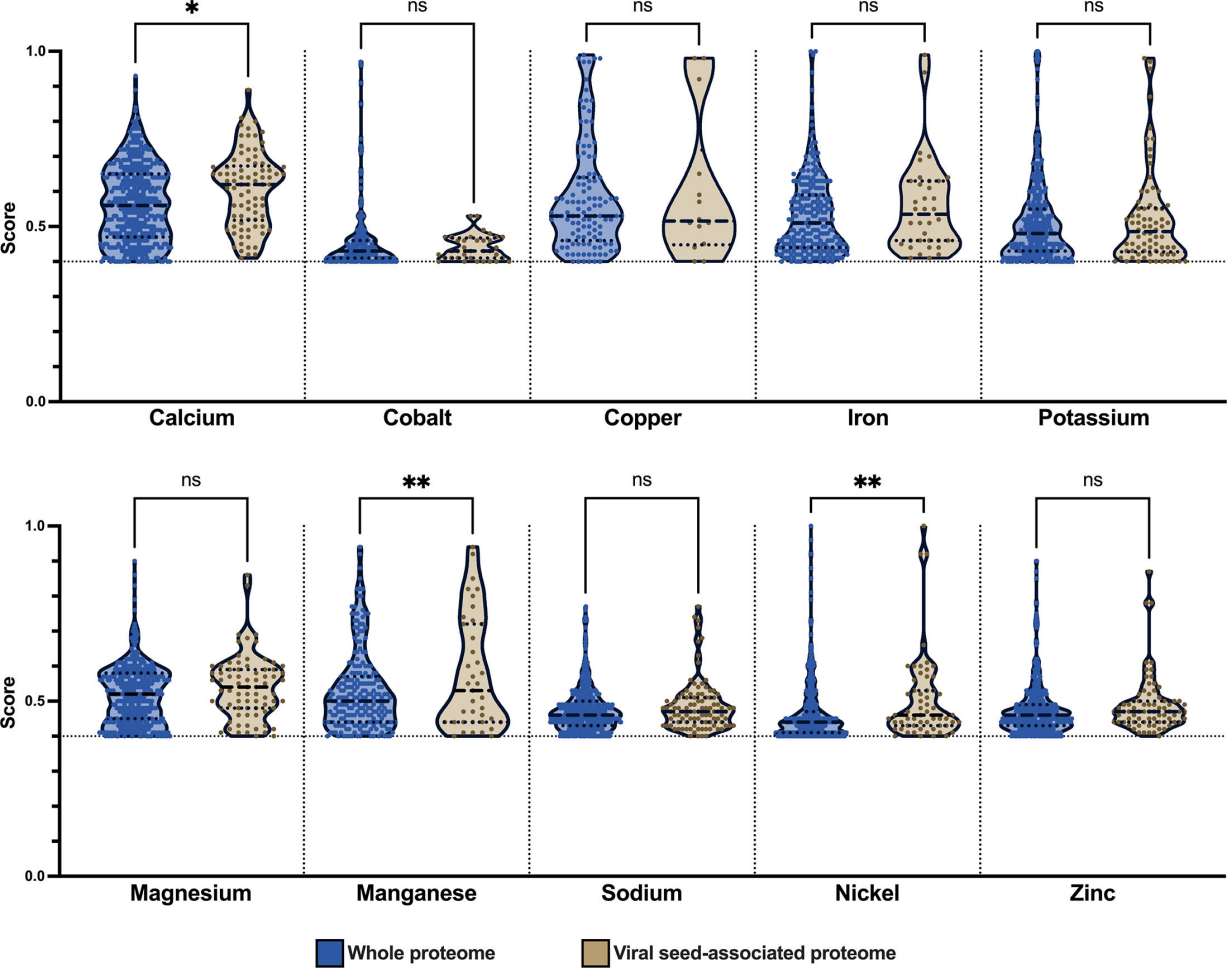
Calcium, manganese, and nickel metal-binding interactions are enriched during early infection. Tupanvirus whole proteome (blue columns) or proteins identified during early infection (tan columns) were analyzed for metal-binding interactions. Distribution of metal-binding interactions above the cutoff value of 0.4 was tested for significant differences that signal enrichment during early infection (*P ≤ 0.05, **P ≤ 0.005). Dashed and dotted lines represent the median and quartiles of the distribution, respectively.

We hypothesized that changes in metal ion concentrations during phagosome maturation could lead to enrichment for particular metal-binding interactions and that these signals could be present in the early infection proteins identified by MS (Fig. 1). When significance was calculated for differences between metal-binding hits for the entire Tupanvirus proteome versus the proteins identified by MS without a cutoff in place, calcium, potassium, magnesium, manganese, and sodium had observed *P* values ≤ 0.001 (data not shown). However, we found that low-scoring metal-binding hits were a major driver of the observed significance, and when only hits above the cutoff of 0.4 were used to determine statistical significance, calcium (*P* ≤ 0.05), manganese (*P* ≤ 0.005), and nickel (*P* ≤ 0.005) were statistically enriched in proteins identified by MS during early infection.

### High concentrations of iron and copper ions induce stargate opening

**Fig 2:**
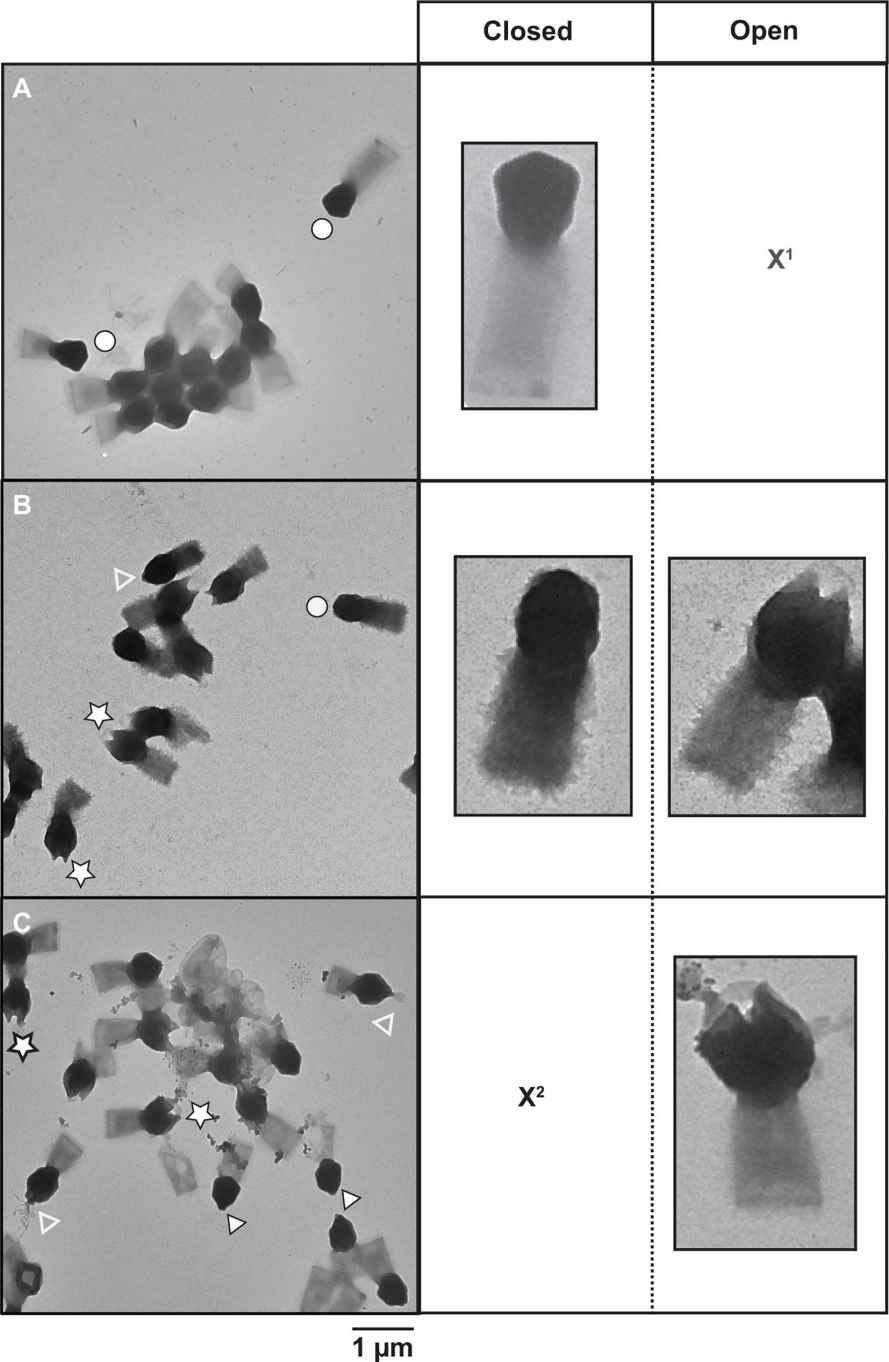
Effect of copper and iron ions on TPV capsids. TEM images of (**A**) untreated TPV, (**B**) 10 mM CuCl_2_-treated particles, and (**C**) 10 mM FeCl_3_-treated particles. Images depict the three stages of stargate features: closed (white circles), pre-opened (white arrowheads), and open (white stars). The empty white arrowheads point at what we hypothesize to be the viral seed exiting the interior of the capsids. X1: open particles not present in this view; X2: closed partciles not present in this view. Scale bar: 1 µm.

**Fig 3:**
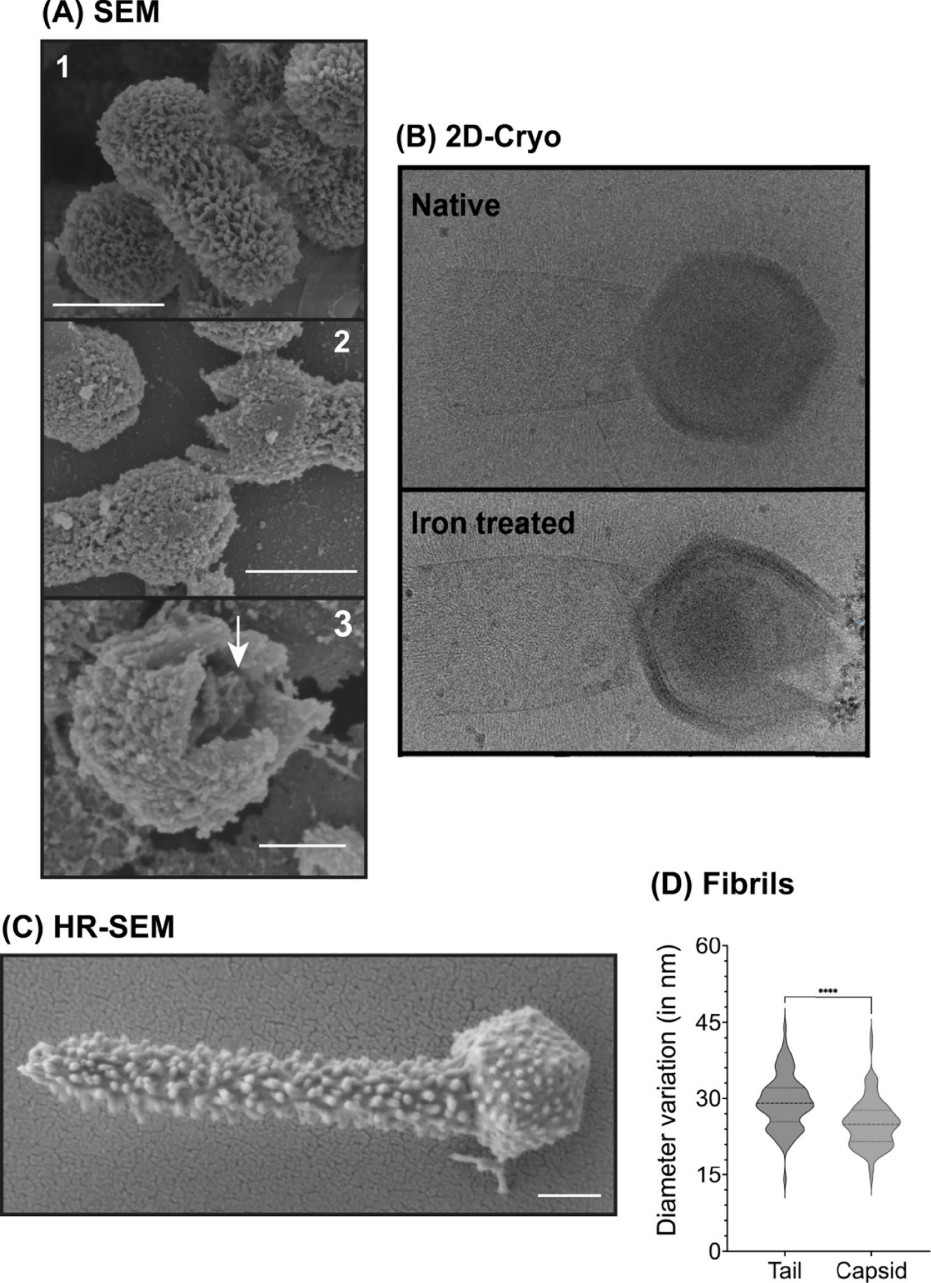
Structural features of TPV particles under native and metal-treated conditions. (**A**) Scanning electron microscopy images of native or copper-treated TPV. Images show (1) the closed, native virus particles; (2) intermediate steps on TPV stargate opening; and (3) the interior of the capsid, where we hypothesize the genome sac is still present even after copper treatment (indicated by the white arrow). Scale bar: 0.5 µm. SEM was not amenable to iron-treated particles, as this metal accumulates on the particle surface. (**B**) 2D cryo-EM images of TPV under native (closed particle, top panel) or iron treated (open stargate, bottom panel); white arrows indicate the genome sac; scale bar: 0.5 µm. (**C**) Ultrahigh-resolution TEM of a native (untreated) TPV particle. In this image, the fibrils are the main feature on the particle surface. The virion shows the tail at its upper-end length, ~1.4 µm. Scale bar: 0.2 µm. (**D**) Based on **C**, fibrils were measured: on average, capsid fibrils measured 25.14 nm, and tail fibrils, 29.29 nm.

*In silico* analysis did not reveal any strong indications that metals are involved in infection enhancement. Thus, we investigated if transition metals could affect the structure of TPV particles. Virions were treated for ~18 hours with 10 mM of either CaCl_2_, CuCl_2_, CoCl_2,_ FeCl_3_, KCl, MgCl_2_, MnCl_2_, NiCl_2_, RbCl, ZnCl_2_, or EDTA. EDTA was used as a control to scavenge divalent or trivalent metals bound to TPV capsids. Transmission electron microscopy (TEM) was used to observe the effects of such treatments on the particle structure, classified as or predominantly associated with (i) no/undetectable effect (CaCl_2_, CoCl_2_, KCl, MgCl_2_, MnCl_2_, NiCl_2_, RbCl, or EDTA), (ii) the tail (ZnCl_2_), or (iii) the capsid (CuCl_2_ and FeCl_3_). ZnCl_2_ disturbed some tails’ integrity, where they appear to have spikes. These are likely due to tangles of the tail fibrils composed of glycans and proteins; the structural modifications observed on the stargate vertex were from the pre-opening stage to the complete opening of the fivefold vertex into a tulip-shaped capsid (Fig. S3; Fig. 2). The pre-opening stage was observed in 65% of the CuCl_2_-treated TPV and 9% of the FeCl_3_-treated particles. Full capsid opening was detected in 30% of CuCl_2_-treated TPV and 72% of FeCl_3_-treated particles. In some copper-treated TPV at the pre-opening stage, a structure exiting the capsid is observed, which we hypothesize as being the viral seed. Scanning electron microscopy of copper-treated TPV particles portrayed all stages of stargate opening (Fig. 3A). 2D cryo-EM of iron-treated TPV (Fig. 3B) and SEM of copper-treated particles indicate in our model that the stargate opening is independent of genome release, as the genome sac is still detected in both imaging techniques (Fig. 3A and B). This may suggest that genome release requires an additional signal to occur. It is noteworthy that iron-treated particles were not amenable to SEM sample preparation due to the extensive deposition of this metal on the particle surface.

### Iron and copper ions accumulate on TPV capsid surface

**Fig 4:**
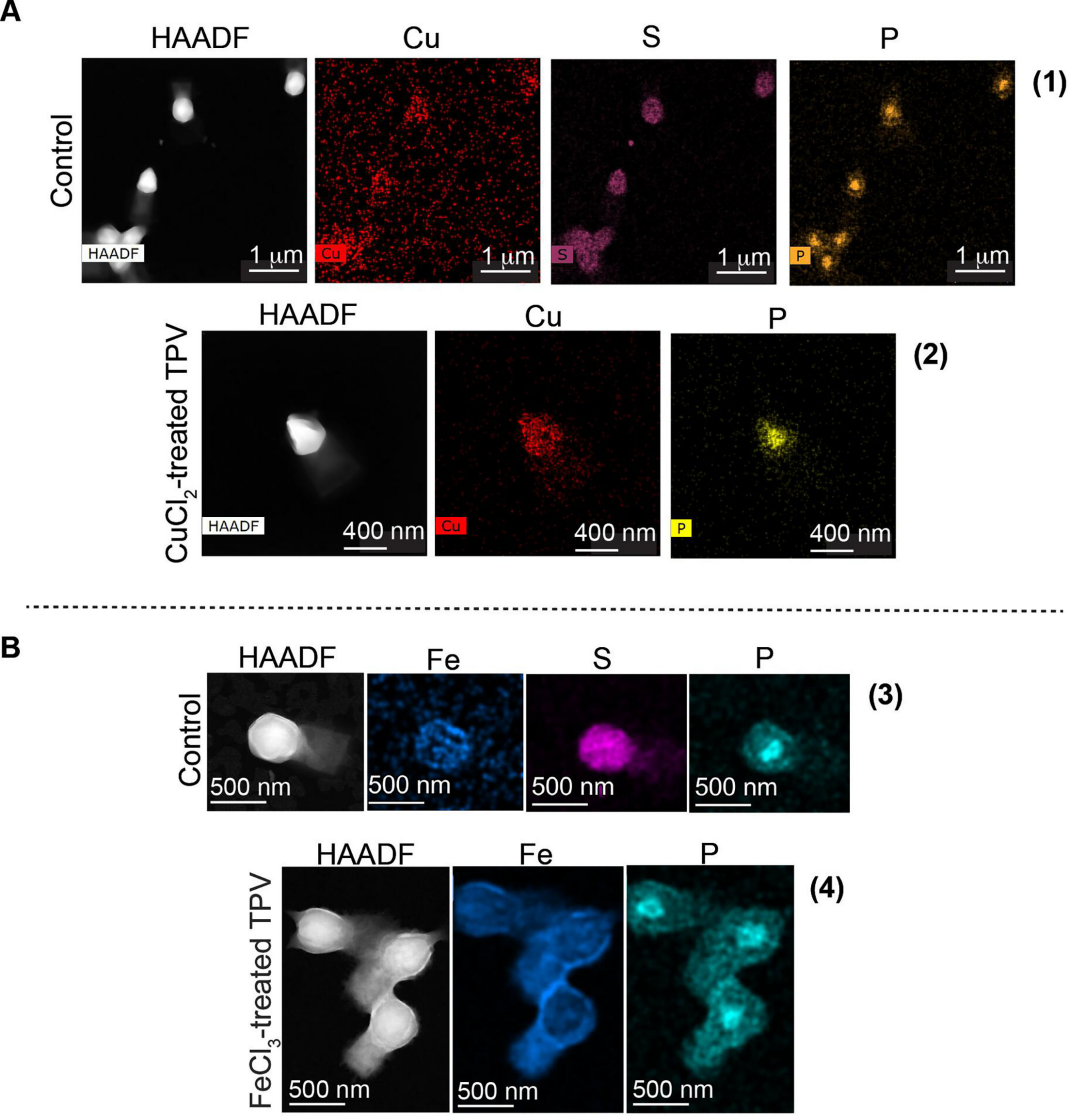
Elemental distribution on TPV surface. TEM associated with energy-dispersive x-ray microanalysis of untreated TPV particles or metal-treated TPV. In these images, the density of colored dots represents the amount of the detected element. (1) Untreated TPV particles and the distribution of the following elements: copper (pink), sulfur (cyan), and phosphorous (dark yellow); (2) 10 mM CuCl_2_-treated TPV particles in the pre-open state. Here, copper is shown in pink and phosphorous in dark-yellow, suggesting the presence of DNA in the interior of the open capsid. (**B**) (3) Untreated TPV particles and the distribution of the following metals: iron (purple), sulfur (cyan), and phosphorous (dark yellow); (4) 10 mM FeCl_3_-treated TPV particles in the open state. Here, the iron electron cloud ghosts the structural feature of the open stargate, but cracks in the capsid are detected. For the elements, iron is shown in purple and phosphorous in dark-yellow, suggesting the presence of DNA in the interior of the open capsid. Scale bars: (**A1**) 1 µm, (**A2**) 400 nm, (**B3** and **B4**) 500 nm. HAADF (high-angle annular dark-field imaging) images were also used to locate the particles on the grid and are the source of element detection.

The high affinity of TPV for copper and iron ions led us to investigate where these metals interact with the viral particles. A study to characterize the different elements in TPV was carried out using TEM associated with energy dispersive x-ray microanalysis (TEM-EDX). Here, the element presence is proportional to the density of the artificially colored dots. The elemental analysis of metal-treated TPV depicts the presence of copper ions mostly on the capsid surface (Fig. 4A, panel 2, pink dots), whereas iron ions were distributed along the capsid and tail (Fig. 4B, panel 4, purple dots). Given this distribution, we hypothesize that surface fibrils may be scavenging these metals. The surface of the TPV capsid is also rich in sulfur (Fig. 4, panels 1 and 3, cyan dots), perhaps associated with the fibrils in the form of sulfated polysaccharide, which can be involved in metal chelation and ROS scavenging (19). Capsid and tail fibril lengths were found to be significantly different, 25.14 nm and 29.29 nm (*P* < 0.0001), respectively, potentially facilitating specific metal affinities (Fig. 3C and D). GV fibrils are built as complex (sometimes unprecedent) glycoproteins (20). The difference in the measured fibril size seen for TPV may be due to protein and/or monosaccharide spatial organization. As observed by TEM-EDX, sulfur is abundantly present on TPV capsid, but not on the tail. The sulfation pattern in saccharides seem to affect metal binding affinities (21), thus corroborating our hypothesis that the differential metal binding pattern is likely related to the fibril structure. Phosphorous ions localize within the interior of the TPV capsid in both CuCl_2_ (Fig. 4A, panel 2, dark yellow) and FeCl_3_ (Fig. 4B, panel 4, dark-yellow) treatments, suggesting that the genome remains within the opened capsids.

### High concentration of H_2_O_2_ also induces stargate opening

**Fig 5:**
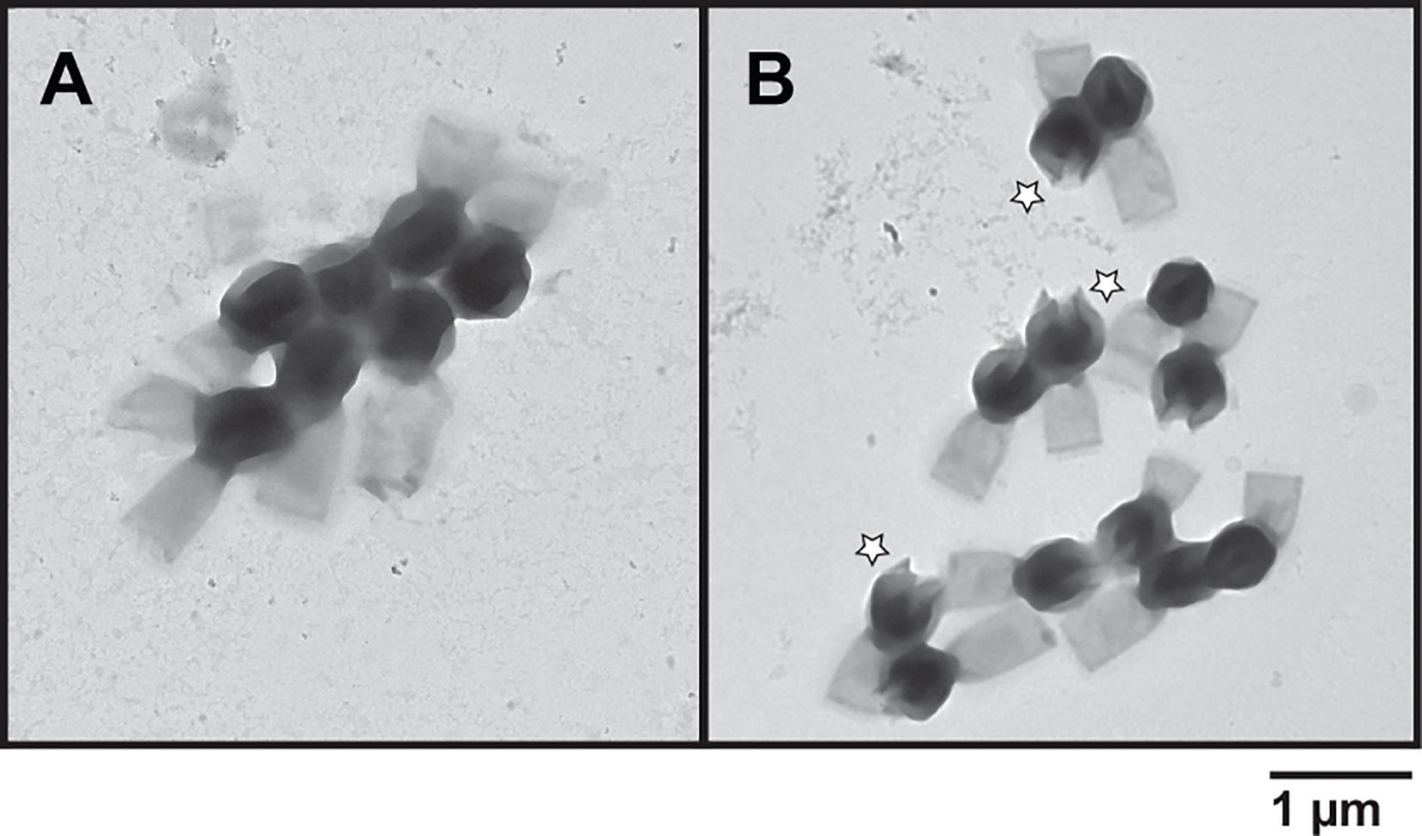
TEM image of H_2_O_2_-treated TPV particles. (**A**) Untreated TPV particles; (**B**) TPV virions were subjected to 2 M H_2_O_2_ treatment, which induced the opening of the stargate, indicated by the white stars. Scale bar: 1 µm.

Because copper and especially iron ions can be involved in oxidation reactions (22, 23), common in the phagosome deadly environment, we hypothesized that H_2_O_2_ may also affect the stargate structure and confirm that the effect on TPV capsid is likely due to protein oxidation/peroxidation (24). Thus, TPV particles were submitted to 2 M H_2_O_2_ for ~18 hours and observed in TEM (Fig. 5). Excitingly, H_2_O_2_ treatment induced stargate opening in 69% of the analyzed particles.

### Infectivity is abolished in metal-treated viruses

**Fig 6:**
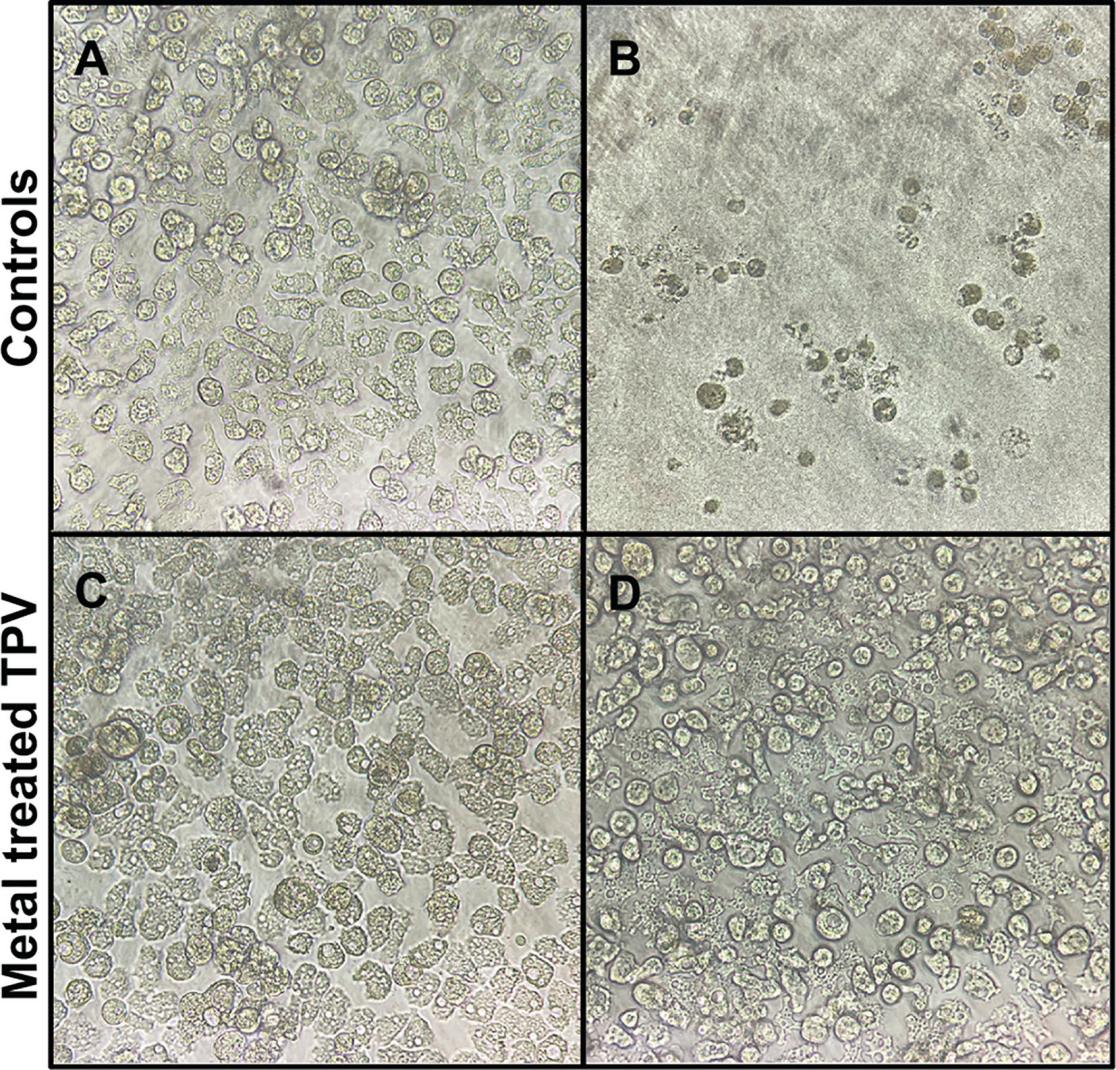
Metal-treated TPV particles are non-infectious. (**A**) Uninfected *Acanthamoeba castellanii* cells, 72 hours of growth; (**B**) untreated TPV titering: 10^−2^ dilution induced complete lysis of *A. castellanii* cells; (**C**) CuCl_2_-treated TPV at 10^−2^ dilution with no apparent cell lysis, characteristic of a non-infectious virus; (**D**) FeCl_3_-treated TPV at 10^−2^ dilution with no apparent cell lysis, characteristic of a non-infectious virus. All pictures are representative of the triplicate dilutions in TCID50 assays observed in an inverted light microscope, with 20× magnification.

Infectivity was measured (25) for CuCl_2_- or FeCl_3_-treated viruses. Relative to untreated TPV, no cytopathic effect was observed even on very low dilutions (10^−2^) of metal-treated particles (Fig. 6). TPV microscopy images suggest that the viral seed is released (Fig. 2) and that the genome sac remains inside the capsid, even though the stargate is wide open (Fig. 3 and 4). Thus, it is likely the viral seed is required for infection and the idea of a second trigger for genome release becomes more likely.

## DISCUSSION

To our surprise, copper and iron metal-binding associations were not found to be significantly enriched during early infection. Additionally, copper and iron were not found to be associated with proteins annotated as capsid or other structural proteins. Possibly due to the novelty of many GV genes, which preclude them from proper annotation. As such, some structural genes that can bind copper or iron could have been misclassified into the “hypothetical” functional category. Furthermore, it is plausible that capsid and solvent-exposed structural genes have evolved to bind copper and iron to sequester ions that engage in the propagation of Fenton chemistry, which could temporally regulate capsid opening during phagosome maturation. Fenton chemistry, oxidation, and acidification can be powerful weapons to fight viral infections within phagosomes. Altogether, they are part of the nutritional immunity strategy.

We suggest that the triggers that initiate infection may be found in the phagosomal vacuole, more specifically in the modulation of transition metals and the oxidative environment controlled by the host cells, known as nutritional immunity. *In vitro*, the presence of iron ions, copper ions, and H_2_O_2_ can promote stargate opening in TPV (Fig. 2 and 5) and is likely inducing the ejection of the viral seed (depicted in Fig. 2 by the white arrowhead), but not the viral genome. We hypothesize that the genome ejection requires a second trigger, since it is still present inside the capsid after iron or copper treatment, as seen by cryo-EM, SEM, and EDX-TEM (Fig. 3A, B and 4). Furthermore, EDX analysis showed differential accumulation of iron and copper ions on virions previously exposed to 10 mM FeCl_3_ or CuCl_2_ (Fig. 4). Both metals rendered the treated viruses non-infectious, likely due to the release of the viral seed (Fig. 2 and 6). In other viruses, excess copper and a lack of iron negatively affect the fidelity of enzymes, hindering their replication cycles (17, 26). Interestingly, when EhV86, an algae GV, is challenged with copper during its replicative cycle, a drop in infectivity is observed. The mechanisms that drive this viral neutralization are yet to be described (27). Our results are remarkable as they demonstrate that the stargate opening can occur *in vitro* in conditions closer to physiological and also offer another method to induce opening *in vitro*. The molecular mechanics of stargate opening remain largely unknown, but the participation of metallic ions and H_2_O_2_ might be associated with structural changes to the seal-like structure. Levels of molecules involved in nutritional immunity processes vary immensely and depend on the invading pathogen (28, 29) but are at least 4 to 100 times lower than the concentrations used here for H_2_O_2_ and metals, respectively (30). One explanation for this discrepancy may be that *in vivo*, the redundancy of mechanisms is overcome *in vitro* by excess concentration of each of the tested molecules. We cannot rule out the incremental participation of other molecules either.

Nutritional immunity and degradation within the phagosome are obstacles that all ingested microbes, including Tupanvirus, must contend with. Production of ROS, decrease in pH, influx of copper and zinc, and efflux of iron and manganese present metabolic challenges for phagocytized bodies (31). However, past (3) and present data from our group suggest Tupanvirus not only withstands but requires some or all of these conditions: low pH, copper ions, iron ions, and ROS for stargate opening and sequential viral replication initiation. Conformational changes and proteolytic cleavages may be induced by peroxidation and oxidation (24), validating our hypothesis that these factors may indeed be involved in stargate opening via proteolysis of a yet unknown stargate protein. In conclusion, TPV evolved mechanisms to overcome nutritional immunity strategies and utilizes these harsh conditions to trigger stargate opening. From an evolutionary point of view, this is extraordinary since phagocytic cells were the primordial forms of life.

## SUPPLEMENTAL MATERIAL

Legends mbio.02192-24-s0001.pdf Supplemental figure legends.

Supplemental material mbio.02192-24-s0002.pdf Supplemental material and methods, figures, and table.

ASM does not own the copyrights to Supplemental Material that may be linked to, or accessed through, an article. The authors have granted ASM a non-exclusive, world-wide license to publish the Supplemental Material files. Please contact the corresponding author directly for reuse.
