# Supplementary material for: Transition metals and oxidation reactions trigger stargate opening during the initial stages of the replicative cycle of the giant Tupanvirus
Source: mBio. 2024 Sep 26;15(10):e02192-24. doi: 10.1128/mbio.02192-24 (PMC11481487; doi:10.1128/mbio.02192-24)
Supplement: Legends — Supplemental figure legends. [file mbio.02192-24-s0001.pdf]

Supplemental Figure Legends

Supplemental Figure S1. Distribution of metal-binding associations from 141 proteins identified by MS above the cutoff value of 0.4 plotted as a percentage of total hits, either by functional category (A) or by metal ion (B).

Supplemental Figure S2. Distribution of metal-binding associations from 141 proteins identified by MS above the cutoff value of 0.4, plotted as a percentage of total hits to metal ions within assigned functional categories (A) or to functional categories pertaining to specific metal ions (B).

Supplemental Figure S3. *TEM images of transition metals-treated TPV particles*. Panels show different metals used to treat TPV particles. Scale bar corresponds to 2 μm.
