# Supplementary material for: Transition metals and oxidation reactions trigger stargate opening during the initial stages of the replicative cycle of the giant Tupanvirus
Source: mBio. 2024 Sep 26;15(10):e02192-24. doi: 10.1128/mbio.02192-24 (PMC11481487; doi:10.1128/mbio.02192-24)
Supplement: Supplemental material — and methods, figures, and table. [file mbio.02192-24-s0002.pdf]

Materials and Methods

Obtaining, purifying, and titrating the viral particles

The TPV virus stocks were obtained through the infection of *A. castellanii* cells (strain 30011) and after 96 hours of infection, the viruses were purified as described by (1). Viral titration by Tissue Culture Infectious Dose (TCID50) method (2).

In silico metal-binding analysis

To identify putative metal-binding proteins in Tupanvirus, translated coding sequences from Tupanvirus deep ocean (GenBank accession MF405918.2, previously Tupanvirus soda lake KY523104.1), were searched using MeBiPred (3) using an identification threshold of 0.4 due to the genomic novelty found in giant viruses. Proteins of interest which have been previously identified by mass spectrometry to be expressed during early infection (4) were further queried against the Conserved Domain Database (5); https://www.ncbi.nlm.nih.gov/cdd/). Statistical analysis and graphing were performed in GraphPad Prism v10 using one-way ANOVA with Sidak's multiple comparisons test. The raw output from MeBiPred are provided in **Supplementary Table S1**.

Iron and copper treatment of virus particles

Tupanvirus particles were incubated overnight at room temperature in the presence of 10 mM of either $CaCl_2$, $CuCl_2$, $CoCl_2$, $FeCl_3$, KCl, $MgCl_2$, $MnCl_2$, $NiCl_2$, RbCl, $ZnCl_2$ or EDTA - diluted in 20 mM HEPES, pH 7.4 or PBS.

Infectivity of copper and iron treated TPV particles

One million amoeba cells were plated on a 96-well plate and incubated overnight. Iron- and copper- treated viruses were washed to extract virion-released material and excess metals that could affect the infectivity assay. Quadruplicates of serial dilutions of the TPV-iron treated or not treated particles were performed, using PYG media as the diluent. Fifty microliter of each dilution was placed over the cell layer, incubated for one hour. Sequentially, 200 μL of PYG was added, and plate was incubated at room temperature for up to 7 days. The plate was observed daily for cytopathic effects and infectivity was calculated using the Tissue Culture Infectious Dose (TCID50) method.

Transmission electronic microscopy of metal treated TPV particles

Five μL of metal treated and untreated Tupanvirus particles were layered on glow discharged FORMVAR coated copper grids (EMS, Hatfield, PA) washed, and air dried. Due to their natural electron density, the samples didn't require any contrasting agent. Images were obtained in a FEI Tecnai 12 G2 Spirit BioTWI, with direct magnification of 9,300 x. Data was captured using an AMT NanoSprint12.

2D Cryo-Electron Microscopy

Small (~5 μL) aliquots of virus particles were applied to R2/2 Quantifoil grids (Electron Microscopy Solutions) that had been plasma cleaned for 60 seconds in a Pelco easiGlow glow discharge unit. The samples were plunge frozen in liquid ethane using a Vitrobot Mark IV set to 4 ˚C and 100% humidity using 4 sec of blotting time per grid. Frozen-hydrated samples were stored, transferred, and imaged under liquid nitrogen temperatures.

Cryo-EM experiments were performed at the RTSF Cryo-EM Core Facility at Michigan State University. Virus particles were imaged using a Talos Arctica operated at 200 keV, under low dose conditions controlled by EPU. Micrographs were recorded on a Ceta camera operating in linear mode. Micrographs were collected at nominal magnifications ranging 11k, 13.5k and 17.5k, (6.03, 7.60, and 9.66 Å/pixel, respectively). The objective lens defocus settings ranged from 5 to 10 μm underfocus. Micrographs were collected for 4-15 seconds, resulting in a total dose of <60 e-/Å2 per sample.

Transmission electron microscopy associated with energy dispersive x-ray microanalysis of metal treated TPV particles

Five μL of metal treated and untreated Tupanvirus particles were layered on glow discharged FORMVAR coated gold grids (Spi Supplies, West Chester, PA), washed and air dried. Due to their natural electron density, samples didn't require any contrasting agent. Elemental analysis experiments were performed with a Talos F200X equipped with a Super-X EDX system. Data were acquired in its STEM mode (convergence angle: 10.5 mrad) with 0.5 nA beam current by Velox software.

Supplemental Materials References

1. Oliveira J dos S, Oliveira DF, Essus VA, Nunes GHP, Honorato L, Oliveira L, Nimrichter L, Peralta JM, Guimaraes AJ, Foguel D, Cortines JR. 2021. Paving the way towards understanding the inflammatory pathways triggered by giant viruses in mammalian cells: effect of mimivirus-cell interactions on IκBα degradation. bioRxiv 2021.09.16.460633.

2. Reed LJ, Muench H. 1938. A simple method of estimating fifty per cent endpoints. Am J Epidemiol 27:493–497.

3. Aptekmann AA, Buongiorno J, Giovannelli D, Glamoclija M, Ferreiro DU, Bromberg Y. 2022. mebipred: identifying metal-binding potential in protein sequence. Bioinformatics 38.

4. Schrad JR, Abrahão JS, Cortines JR, Parent KN. 2020. Structural and Proteomic Characterization of the Initiation of Giant Virus Infection. Cell 181:1046-1061.e6.

5. Lu S, Wang J, Chitsaz F, Derbyshire MK, Geer RC, Gonzales NR, Gwadz M, Hurwitz DI, Marchler GH, Song JS, Thanki N, Yamashita RA, Yang M, Zhang D, Zheng C, Lanczycki CJ, Marchler-Bauer A. 2020. CDD/SPARCLE: The conserved domain database in 2020. Nucleic Acids Res 48.

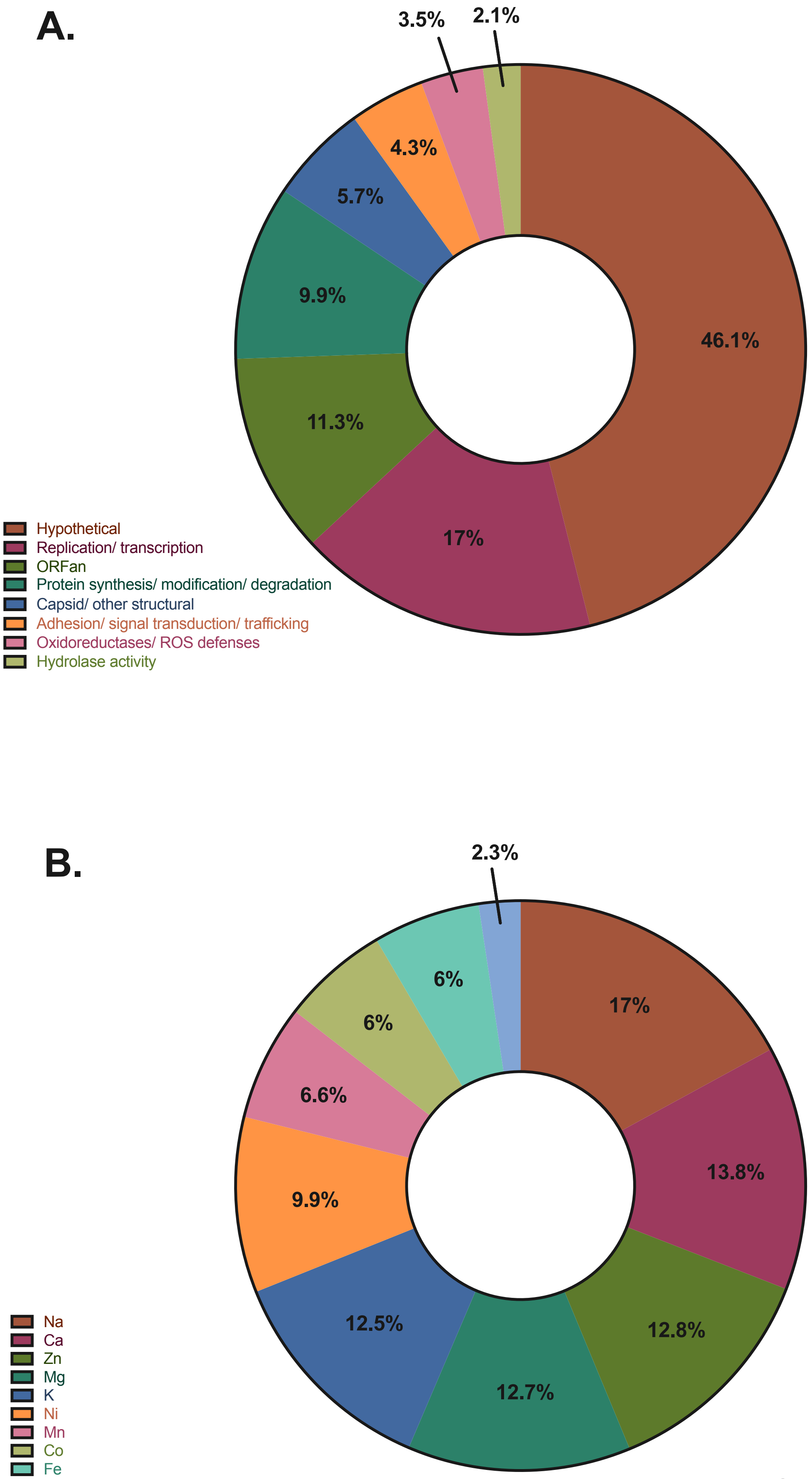
A.
3.5%
2.1%
4.3%
5.7%
9.9%
46.1%
11.3%
17%
Hypothetical
Replication/ transcription
ORFan
Protein synthesis/ modification/ degradation
Capsid/ other structural
Adhesion/ signal transduction/ trafficking
Oxidoreductases/ ROS defenses
Hydrolase activity
B.
2.3%
6%
17%
6%
6.6%
13.8%
9.9%
12.5%
12.8%
12.7%
Na
Ca
Zn
Mg
K
Ni
Mn
Co
Fe
Cu

Figure S1

Figure S2

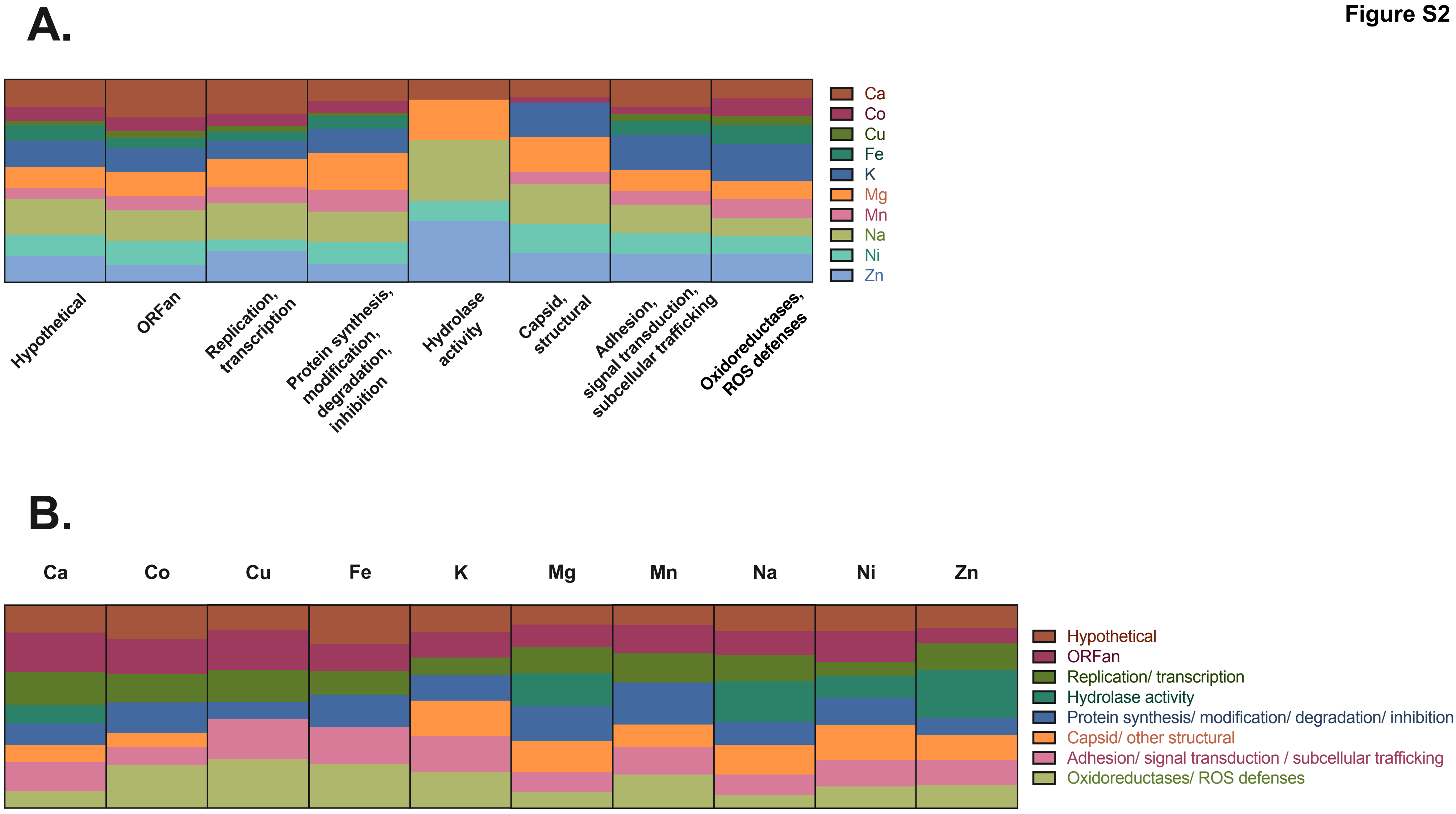
A.
Ca
Co
Cu
Fe
K
Mg
Mn
Na
Ni
Zn
Hypothetical
ORFan
Replication, transcription
Protein synthesis, modification, degradation, inhibition
Hydrolase activity
Capsid, structural
Adhesion, signal transduction, subcellular trafficking
Oxidoreductases, ROS defenses
B.
Ca
Co
Cu
Fe
K
Mg
Mn
Na
Ni
Zn
Hypothetical
ORFan
Replication/ transcription
Hydrolase activity
Protein synthesis/ modification/ degradation/ inhibition
Capsid/ other structural
Adhesion/ signal transduction / subcellular trafficking
Oxidoreductases/ ROS defenses

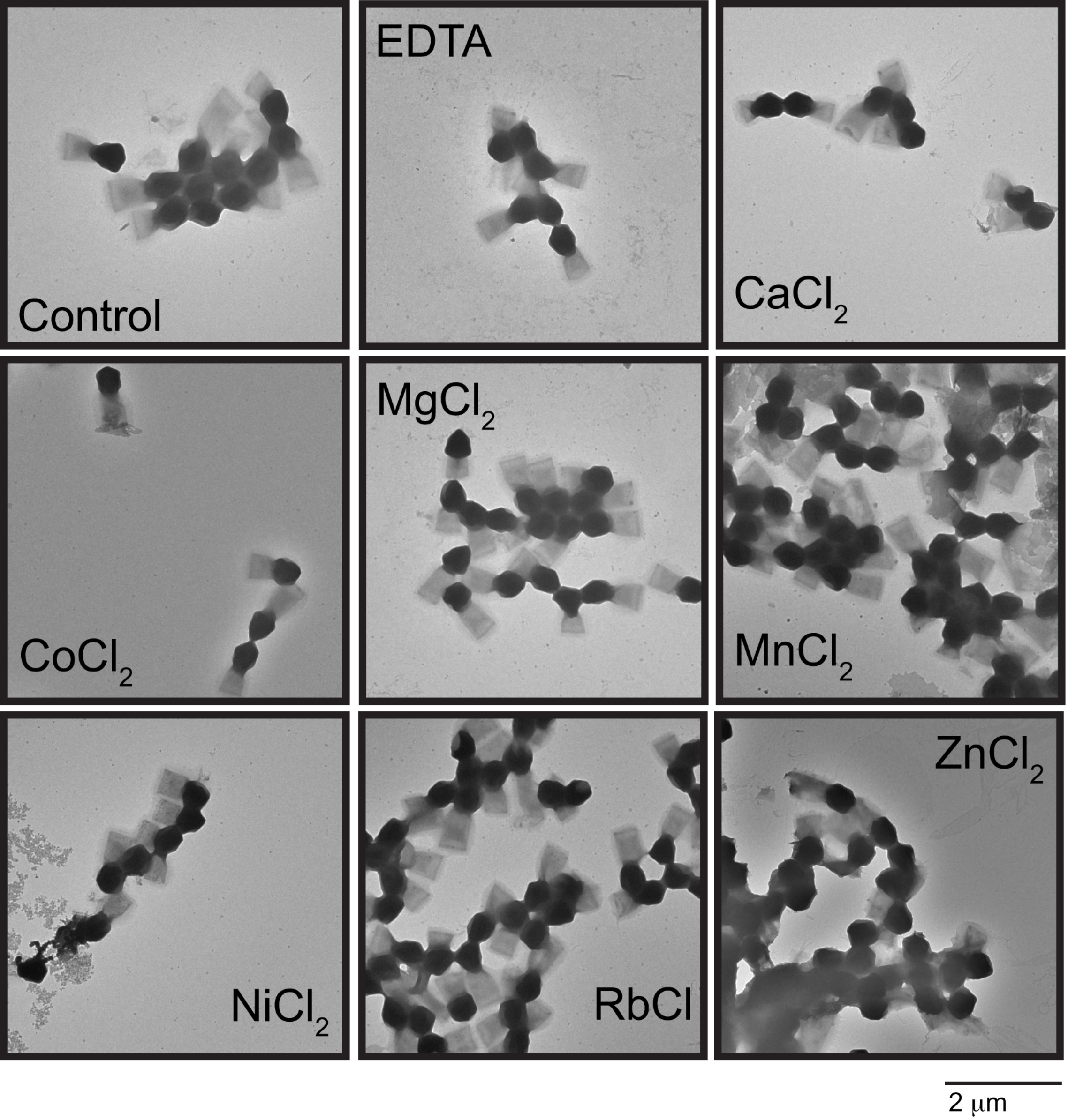

Figure S3

#	**Summary: Metal Binding Proteins above treshold of 0.5**										
# SUPPLMENTAL TABLE 1 *Raw output from MeBiPred*		Ca	Co	Cu	Fe	K	Mg	Mn	Na	Ni	Zn
#		302	25	74	134	158	265	119	133	102	112
ID	MetalBinding	Ca	Co	Cu	Fe	K	Mg	Mn	Na	Ni	Zn
KY523104.1_AUL77469.1_MF405918.2_AUL78745.2_MG777_PROTEIN_CDS	0.38	0.51	0.63	0.29	0.32	0.4	0.22	0.53	0.47	0.36	0.37
KY523104.1_AUL77470.1_MF405918.2_AUL78746.2_FORK_HEAD_DOMAIN-CONTAINING_PROTEIN_CDS	0.58	0.34	0.44	0.28	0.44	0.34	0.19	0.18	0.27	0.45	0.46
KY523104.1_AUL77471.1_MF405918.2_AUL78747.2_PUTATIVE_ORFAN_CDS	0.2	0.28	0.25	0.09	0.01	0.18	0.37	0.11	0.46	0.43	0.12
KY523104.1_AUL77472.1_MF405918.2_AUL78748.2_HYPOTHETICAL_PROTEIN_CDS	0.23	0.14	0.28	0.04	0.02	0.28	0.28	0.15	0.19	0.44	0.17
KY523104.1_AUL77473.1_MF405918.2_QKU33423.1_PUTATIVE_ORFAN_CDS	0.39	0.68	0.42	0.22	0.01	0.27	0.37	0.2	0.42	0.1	0.19
KY523104.1_AUL77474.1_MF405918.2_QKU33424.1_PHOSPHATIDYLETHANOLAMINE-BINDING_PROTEIN-LIKE_PROTEIN	0.18	0.24	0.16	0.02	0.28	0.12	0.11	0.03	0.03	0.31	0.19
KY523104.1_AUL77475.1_MF405918.2_QKU33425.1_HYPOTHETICAL_PROTEIN_CDS	0.4	0.15	0.33	0.25	0.51	0.6	0.54	0.58	0.42	0.53	0.43
KY523104.1_AUL77476.1_MF405918.2_QKU33426.1_PUTATIVE_ORFAN_CDS	0.31	0.08	0.21	0.05	0.01	0.26	0.16	0.07	0.14	0.38	0.4
KY523104.1_AUL77477.1_MF405918.2_QKU33427.1_HYPOTHETICAL_PROTEIN_CDS	0.4	0.93	0.08	0.14	0	0	0.04	0	0.37	0	0
KY523104.1_AUL77478.1_MF405918.2_QKU33428.1_METHIONYL-TRNA_SYNTHETASE_CDS	0.75	0.73	0.46	0.23	0.31	0.5	0.25	0.13	0.45	0.44	0.56
KY523104.1_AUL77479.1_MF405918.2_QKU33429.1_PUTATIVE_ORFAN_CDS	0.13	0.65	0.16	0.01	0	0.09	0.04	0	0.01	0.32	0.03
KY523104.1_AUL77480.1_MF405918.2_QKU33430.1_MANNOSE-SPECIFIC_LECTIN_CDS	0.25	0.31	0.22	0.14	0.05	0.28	0.2	0.04	0.25	0.41	0.34
KY523104.1_AUL77481.1_MF405918.2_QKU33431.1_PUTATIVE_LECTIN_CDS	0.11	0.14	0.15	0.02	0.05	0.09	0.16	0.02	0.58	0.37	0.08
KY523104.1_AUL77482.1_MF405918.2_QKU33432.1_HYPOTHETICAL_PROTEIN_CDS	0.4	0.54	0.39	0.33	0.51	0.43	0.2	0.37	0.43	0.33	0.44
KY523104.1_AUL77483.1_MF405918.2_QKU33433.1_HYPOTHETICAL_PROTEIN_CDS	0.23	0.32	0.34	0.58	0.05	0.26	0.21	0.09	0.27	0.44	0.16
KY523104.1_AUL77484.1_MF405918.2_QKU33434.1_HYPOTHETICAL_PROTEIN_CDS	0.26	0.25	0.29	0.11	0.02	0.29	0.43	0.4	0.05	0.39	0.38
KY523104.1_AUL77485.1_MF405918.2_QKU33435.1_MG971_PROTEIN_CDS	0.4	0.35	0.07	0	0	0.23	0.62	0.13	0.33	0.04	0.17
KY523104.1_AUL77486.1_MF405918.2_QKU33436.1_HYPOTHETICAL_PROTEIN_CDS	0.72	0.58	0.29	0.23	0.19	0.41	0.42	0.04	0.44	0.43	0.51
KY523104.1_AUL77487.1_MF405918.2_QKU33437.1_ANKYRIN_CDS	0.29	0.23	0.39	0.42	0.06	0.38	0.46	0.38	0.3	0.41	0.39
KY523104.1_AUL77488.1_MF405918.2_QKU33438.1_MG760_PROTEIN_CDS	0.27	0.43	0.38	0.12	0.4	0.33	0.41	0.49	0.24	0.37	0.3
KY523104.1_AUL77489.1_MF405918.2_QKU33439.1_HYPOTHETICAL_PROTEIN_CDS	0.14	0.14	0.13	0.01	0	0.06	0.16	0.01	0.33	0.37	0.07
KY523104.1_AUL77490.1_MF405918.2_QKU33440.1_NUCLEIC_ACID-BINDING_PROTEIN_CDS	0.22	0.39	0.36	0.05	0.14	0.28	0.29	0.51	0.28	0.32	0.22
KY523104.1_AUL77491.1_MF405918.2_QKU33441.1_PUTATIVE_TRANSMEMBRANE_PROTEIN_CDS	0.36	0.17	0.31	0.26	0.04	0.36	0.33	0.47	0.49	0.43	0.43
KY523104.1_AUL77492.1_MF405918.2_QKU33442.1_HYPOTHETICAL_PROTEIN_CDS	0.21	0.29	0.37	0.1	0.34	0.52	0.38	0.25	0.24	0.48	0.13
KY523104.1_AUL77493.1_MF405918.2_QKU33443.1_CYTOCHROME_B5-LIKE_PROTEIN_CDS	0.15	0.08	0.11	0	0.24	0.1	0.15	0.02	0.08	0.29	0.21
KY523104.1_AUL77494.1_MF405918.2_QKU33444.1_PUTATIVE_ORFAN_CDS	0.33	0.22	0.4	0.45	0.26	0.43	0.18	0.56	0.4	0.31	0.38
KY523104.1_AUL77495.1_MF405918.2_QKU33445.1_PUTATIVE_ORFAN_CDS	0.24	0.06	0.17	0.01	0.42	0.12	0.11	0.03	0.02	0.34	0.3
KY523104.1_AUL77495.1_MF405918.2_QKU33445.1_PUTATIVE_ORFAN_CDS	0.1	0.03	0.11	0	0.02	0.07	0.29	0.02	0.15	0.29	0.14
KY523104.1_AUL77494.1_MF405918.2_QKU33444.1_PUTATIVE_ORFAN_CDS	0.09	0.02	0.11	0	0.02	0.07	0.28	0.02	0.15	0.29	0.13
KY523104.1_AUL77496.1_MF405918.2_QKU33446.1_PUTATIVE_ORFAN_CDS	0.3	0.48	0.37	0.1	0.22	0.54	0.26	0.15	0.3	0.41	0.37
KY523104.1_AUL77497.1_MF405918.2_QKU33447.1_PUTATIVE_ORFAN_CDS	0.19	0.06	0.14	0.01	0.01	0.31	0.21	0.06	0.07	0.3	0.16
KY523104.1_AUL77498.1_MF405918.2_AUL78773.2_HYPOTHETICAL_PROTEIN_CDS	0.29	0.4	0.36	0.17	0.01	0.51	0.48	0.44	0.36	0.39	0.35
KY523104.1_AUL77499.1_MF405918.2_QKU33448.1_PUTATIVE_ORFAN_CDS	0.61	0.21	0.38	0.36	0.49	0.31	0.24	0.16	0.4	0.46	0.46
KY523104.1_AUL77500.1_MF405918.2_QKU33449.1_HYPOTHETICAL_PROTEIN_CDS	0.4	0.74	0.32	0.17	0.37	0.21	0.58	0.44	0.38	0	0.14
KY523104.1_AUL77501.1_MF405918.2_QKU33450.1_HYPOTHETICAL_PROTEIN_CDS	0.18	0.06	0.33	0.02	0.01	0.16	0.33	0.03	0.05	0.39	0.08
KY523104.1_AUL77502.1_MF405918.2_QKU33451.1_PUTATIVE_ORFAN_CDS	0.16	0.11	0.22	0.02	0.02	0.46	0.31	0.03	0.08	0.43	0.08
KY523104.1_AUL77503.1_MF405918.2_AUL78778.2_PUTATIVE_ORFAN_CDS	0.25	0.08	0.17	0.04	0	0.51	0.13	0.02	0.12	0.35	0.34
KY523104.1_AUL77504.1_MF405918.2_QKU33452.1_PUTATIVE_ORFAN_CDS	0.27	0.4	0.36	0.13	0.04	0.3	0.29	0.43	0.17	0.34	0.39

KY523104.1_AUL77505.1_MF405918.2_QKU33453.1_PUTATIVE_ORFAN_CDS	0.17	0.17	0.15	0.57	0	0.08	0.2	0.03	0.06	0.27	0.18
KY523104.1_AUL77506.1_MF405918.2_QKU33454.1_HYPOTHETICAL_PROTEIN_CDS	0.74	0.58	0.28	0.27	0.39	0.3	0.45	0.03	0.47	0.6	0.62
KY523104.1_AUL77507.1_MF405918.2_QKU33455.1_MIMIVIRUS_TRANSLATION_INITIATION_FACTOR_2_GAMMA_SUB	0.73	0.47	0.24	0.19	0.09	0.27	0.47	0.05	0.44	0.42	0.52
KY523104.1_AUL77508.1_MF405918.2_QKU33456.1_PUTATIVE_ORFAN_CDS	0.38	0.06	0.45	0.97	0	0.36	0.02	0.09	0.33	0.29	0.41
KY523104.1_AUL77509.1_MF405918.2_QKU33457.1_PUTATIVE_ANKYRIN_REPEAT_PROTEIN_CDS	0.44	0.2	0.29	0.46	0.34	0.31	0.39	0.1	0.39	0.49	0.47
KY523104.1_AUL77510.1_MF405918.2_QKU33458.1_PUTATIVE_ANKYRIN_REPEAT_PROTEIN_CDS	0.4	0.58	0.3	0.04	0.13	0.28	0.58	0.41	0.45	0.1	0.38
KY523104.1_AUL77511.1_MF405918.2_QKU33459.1_PUTATIVE_GTP-BINDING_PROTEIN_CDS	0.23	0.28	0.29	0.12	0.14	0.29	0.52	0.16	0.21	0.31	0.25
KY523104.1_AUL77512.1_MF405918.2_QKU33460.1_MACROCIN_O-METHYLTRANSFERASE_CDS	0.23	0.54	0.41	0.14	0.07	0.33	0.25	0.39	0.35	0.31	0.27
KY523104.1_AUL77513.1_MF405918.2_QKU33461.1_MG19_PROTEIN_CDS	0.22	0.21	0.29	0.07	0	0.38	0.4	0.12	0.19	0.43	0.18
KY523104.1_AUL77514.1_MF405918.2_QKU33462.1_HYPOTHETICAL_PROTEIN_CDS	0.38	0.54	0.42	0.31	0.44	0.37	0.35	0.36	0.5	0.4	0.35
KY523104.1_AUL77515.1_MF405918.2_QKU33463.1_PUTATIVE_ORFAN_CDS	0.22	0.12	0.23	0.02	0.44	0.15	0.4	0.06	0.11	0.37	0.14
KY523104.1_AUL77516.1_MF405918.2_QKU33464.1_MIMIVIRUS_GTP-BINDING_TRANSLATION_ELONGATION/INITIATIC	0.4	0.73	0.35	0.8	0	0.92	0.57	0.28	0.48	0	0.08
KY523104.1_AUL77517.1_MF405918.2_QKU33465.1_MG749_PROTEIN_CDS	0.48	0.74	0.43	0.35	0.25	0.25	0.32	0.17	0.43	0.21	0.5
KY523104.1_AUL77518.1_MF405918.2_QKU33466.1_TYROSINE-PROTEIN_PHOSPHATASE_CDS	0.24	0.18	0.16	0.04	0	0.1	0.13	0.02	0.37	0.43	0.22
KY523104.1_AUL77519.1_MF405918.2_QKU33467.1_PUTATIVE_ORFAN_CDS	0.39	0.67	0.43	0.33	0.58	0.34	0.11	0.25	0.4	0.19	0.2
KY523104.1_AUL77520.1_MF405918.2_QKU33468.1_HYPOTHETICAL_PROTEIN_CDS	0.55	0.04	0.22	0.1	0	0.17	0.08	0.08	0.12	0.35	0.47
KY523104.1_AUL77521.1_MF405918.2_QKU33469.1_HYPOTHETICAL_PROTEIN_CDS	0.29	0.44	0.35	0.07	0	0.56	0.19	0.23	0.47	0.4	0.32
KY523104.1_AUL77522.1_MF405918.2_QKU33470.1_MG747_PROTEIN_CDS	0.36	0.59	0.39	0.16	0.12	0.56	0.35	0.31	0.43	0.33	0.38
KY523104.1_AUL77523.1_MF405918.2_QKU33471.1_ANKYRIN_REPEAT_PROTEIN_CDS	0.27	0.43	0.45	0.14	0	0.5	0.3	0.11	0.43	0.53	0.24
KY523104.1_AUL77524.1_MF405918.2_QKU33472.1_HYPOTHETICAL_PROTEIN_CDS	1	0.46	0.15	0.01	0	0.98	0.05	0	0.55	0.03	0.9
KY523104.1_AUL77525.1_MF405918.2_QKU33473.1_PUTATIVE_ORFAN_CDS	0.22	0.25	0.13	0.03	0	0.16	0.13	0.01	0.02	0.37	0.31
KY523104.1_AUL77524.1_MF405918.2_QKU33472.1_HYPOTHETICAL_PROTEIN_CDS	0.09	0.28	0.07	0	0	0.03	0.04	0	0.01	0.2	0.02
KY523104.1_AUL77526.1_MF405918.2_QKU33474.1_MG746_PROTEIN_CDS	0.44	0.41	0.32	0.24	0.36	0.35	0.29	0.15	0.37	0.4	0.44
KY523104.1_AUL77527.1_MF405918.2_QKU33475.1_GLUCOSAMINE-FRUCTOSE-6-PHOSPHATE_AMINOTRANSFERASE_C	0.35	0.31	0.34	0.4	0.14	0.35	0.52	0.2	0.47	0.36	0.43
KY523104.1_AUL77528.1_MF405918.2_QKU33476.1_PUTATIVE_ORFAN_CDS	0.25	0.38	0.24	0.08	0.02	0.21	0.15	0.07	0.03	0.36	0.35
KY523104.1_AUL77529.1_MF405918.2_QKU33477.1_PUTATIVE_ORFAN_CDS	0.3	0.58	0.39	0.16	0.04	0.32	0.22	0.2	0.35	0.31	0.39
KY523104.1_AUL77530.1_MF405918.2_QKU33478.1_PUTATIVE_ORFAN_CDS	0.18	0.04	0.14	0.01	0.02	0.09	0.19	0.02	0.39	0.34	0.31
KY523104.1_AUL77531.1_MF405918.2_QKU33479.1_HYPOTHETICAL_PROTEIN_CDS	0.4	0.64	0.44	0.03	0.04	0.38	0.62	0.25	0.46	0.04	0.4
KY523104.1_AUL77532.1_MF405918.2_QKU33480.1_PUTATIVE_ORFAN_CDS	0.36	0.48	0.43	0.07	0.35	0.5	0.5	0.44	0.41	0.6	0.34
KY523104.1_AUL77533.1_MF405918.2_QKU33481.1_PUTATIVE_ORFAN_CDS	0.4	0.65	0.35	0.14	0	0.68	0.5	0.26	0.5	0.27	0.35
KY523104.1_AUL77534.1_MF405918.2_QKU33482.1_ASPARAGINYL_T-RNA_SYNTHETASE_CDS	0.64	0.47	0.32	0.27	0.39	0.53	0.42	0.37	0.43	0.37	0.49
KY523104.1_AUL77535.1_MF405918.2_QKU33483.1_HYPOTHETICAL_PROTEIN_CDS	0.19	0.22	0.25	0.03	0	0.56	0.1	0.12	0.15	0.24	0.09
KY523104.1_AUL77536.1_MF405918.2_QKU33484.1_MG741_PROTEIN_CDS	0.87	0.83	0.47	0.25	0.48	0.55	0.32	0.11	0.42	0.07	0.73
KY523104.1_AUL77536.1_MF405918.2_QKU33484.1_MG741_PROTEIN_CDS	0.21	0.26	0.34	0.58	0.01	0.3	0.41	0.16	0.4	0.44	0.13
KY523104.1_AUL77537.1_MF405918.2_QKU33485.1_PUTATIVE_ORFAN_CDS	0.21	0.26	0.34	0.58	0.01	0.3	0.41	0.16	0.4	0.44	0.13
KY523104.1_AUL77538.1_MF405918.2_QKU33486.1_PUTATIVE_ORFAN_CDS	0.33	0.36	0.26	0.16	0.01	0.28	0.17	0.03	0.2	0.4	0.42
KY523104.1_AUL77539.1_MF405918.2_QKU33487.1_PHOSPHOINOSITIDE_3-KINASE_CDS	0.68	0.47	0.34	0.27	0.18	0.54	0.37	0.06	0.48	0.38	0.55
KY523104.1_AUL77540.1_MF405918.2_QKU33488.1_ALKYLATED_DNA_REPAIR_PROTEIN_ALKB-LIKE_8_ISOFORMX1_CD	0.27	0.21	0.28	0.23	0.01	0.31	0.23	0.34	0.19	0.39	0.38
KY523104.1_AUL77541.1_MF405918.2_QKU33489.1_STOMATIN_FAMILY_PROTEIN/PROHIBITIN-FAMILY_MEMBRANE_P	0.34	0.05	0.46	0.2	0	0.35	0.56	0.07	0.47	0.36	0.42
KY523104.1_AUL77542.1_MF405918.2_QKU33490.1_GLYCOSYL_TRANSFERASE_FAMILY_PROTEIN_CDS	0.29	0.24	0.27	0.18	0.09	0.28	0.26	0.3	0.14	0.39	0.42
KY523104.1_AUL77543.1_MF405918.2_QKU33491.1_PUTATIVE_ORFAN_CDS	0.19	0.07	0.17	0.01	0.02	0.09	0.3	0.07	0.05	0.34	0.24
KY523104.1_AUL77544.1_MF405918.2_QKU33492.1_MG735_PROTEIN_CDS	0.8	0.12	0.28	0.33	0	0.38	0.2	0.08	0.44	0.34	0.57

KY523104.1_AUL77545.1_MF405918.2_QKU33493.1_PUTATIVE_ORFAN_CDS	0.21	0.19	0.18	0.03	0	0.11	0.16	0.06	0.25	0.43	0.21
KY523104.1_AUL77546.1_MF405918.2_QKU33494.1_MG989_PROTEIN_CDS	0.28	0.25	0.33	0.04	0	0.31	0.54	0.53	0.38	0.4	0.26
KY523104.1_AUL77547.1_MF405918.2_QKU33495.1_PUTATIVE_ORFAN_CDS	0.71	0.17	0.43	0.8	0.48	0.34	0.17	0.4	0.44	0.39	0.52
KY523104.1_AUL77548.1_MF405918.2_QKU33496.1_PUTATIVE_HISTONE_DEMETHYLASE_CDS	0.65	0.5	0.35	0.22	0.22	0.41	0.44	0.55	0.35	0.32	0.47
KY523104.1_AUL77549.1_MF405918.2_QKU33497.1_PUTATIVE_ORFAN_CDS	0.03	0.01	0.1	0	0	0.05	0.3	0.01	0.26	0.32	0.02
KY523104.1_AUL77550.1_MF405918.2_QKU33498.1_PUTATIVE_ORFAN_CDS	0.01	0.01	0.03	0	0	0.02	0.06	0	0.01	0.13	0.04
KY523104.1_AUL77551.1_MF405918.2_QKU33499.1_HYPOTHETICAL_PROTEIN_CDS	0.33	0.06	0.27	0.16	0.08	0.27	0.15	0.11	0.48	0.41	0.44
KY523104.1_AUL77552.1_MF405918.2_QKU33500.1_PUTATIVE_ORFAN_CDS	0.23	0.09	0.12	0.01	0.03	0.06	0.29	0.02	0.2	0.34	0.24
KY523104.1_AUL77553.1_MF405918.2_QKU33501.1_MANNOSE-6P_ISOMERASE_CDS	0.25	0.24	0.19	0.16	0.01	0.35	0.18	0.03	0.09	0.42	0.28
KY523104.1_AUL77554.1_MF405918.2_QKU33502.1_HYPOTHETICAL_PROTEIN_CDS	0.29	0.12	0.27	0.03	0.01	0.3	0.56	0.13	0.15	0.28	0.31
KY523104.1_AUL77555.1_MF405918.2_QKU33503.1_PUTATIVE_LEUCINE-RICH_REPEAT-CONTAINING_PROTEIN_37B_CD	0.23	0.36	0.34	0.11	0.01	0.36	0.47	0.47	0.25	0.41	0.24
KY523104.1_AUL77556.1_MF405918.2_QKU33504.1_PUTATIVE_ORFAN_CDS	0.25	0.49	0.39	0.16	0.19	0.31	0.26	0.15	0.37	0.38	0.25
KY523104.1_AUL77557.1_MF405918.2_QKU33505.1_PUTATIVE_ORFAN_CDS	0.33	0.39	0.34	0.28	0.11	0.39	0.3	0.09	0.31	0.41	0.43
KY523104.1_AUL77558.1_MF405918.2_QKU33506.1_PUTATIVE_SERPIN-LIKE_PROTEIN_CDS	0.39	0.46	0.34	0.28	0.06	0.41	0.35	0.34	0.47	0.38	0.44
KY523104.1_AUL77559.1_MF405918.2_QKU33507.1_PUTATIVE_ORFAN_CDS	0.23	0.21	0.15	0.03	0.31	0.11	0.1	0.02	0.06	0.38	0.29
KY523104.1_AUL77560.1_MF405918.2_QKU33508.1_PHOSPHOMEVALONATE_KINASE_CDS	0.17	0.18	0.26	0.03	0.01	0.22	0.45	0.05	0.24	0.38	0.08
KY523104.1_AUL77561.1_MF405918.2_QKU33509.1_PUTATIVE_ORFAN_CDS	0.39	0.32	0.45	0.07	0.63	0.35	0.27	0.38	0.25	0.62	0.38
KY523104.1_AUL77562.1_MF405918.2_QKU33510.1_P-LOOP_ATPASE/GTPASE-CONTAINING_DOMAIN_PROTEIN_CDS	0.4	0.61	0.29	0.07	0.04	0.35	0.57	0.32	0.49	0.12	0.43
KY523104.1_AUL77563.1_MF405918.2_QKU33511.1_PUTATIVE_ORFAN_CDS	0.14	0.09	0.16	0.01	0.37	0.12	0.12	0.03	0.31	0.4	0.08
KY523104.1_AUL77564.1_MF405918.2_QKU33512.1_PUTATIVE_HELICASE_CDS	0.32	0.46	0.34	0.3	0.04	0.39	0.3	0.13	0.48	0.44	0.38
KY523104.1_AUL77565.1_MF405918.2_QKU33513.1_PUTATIVE_ORFAN_CDS	0.2	0.14	0.2	0.04	0.24	0.39	0.3	0.05	0.08	0.41	0.12
KY523104.1_AUL77566.1_MF405918.2_QKU33514.1_PUTATIVE_PHOSPHATIDYLCHOLINE_CERAMIDE_CHOLINEPHOSPH(	0.2	0.14	0.2	0.04	0.24	0.39	0.3	0.05	0.08	0.41	0.12
KY523104.1_AUL77566.1_MF405918.2_QKU33514.1_PUTATIVE_PHOSPHATIDYLCHOLINE_CERAMIDE_CHOLINEPHOSPH(	0.31	0.2	0.2	0.08	0.02	0.19	0.17	0.03	0.07	0.41	0.4
KY523104.1_AUL77565.1_MF405918.2_QKU33513.1_PUTATIVE_ORFAN_CDS	0.38	0.12	0.16	0.01	0	0.12	0.09	0.01	0.03	0.36	0.43
KY523104.1_AUL77567.1_MF405918.2_QKU33515.1_VALINE_TRNA-LIGASE_CDS	0.45	0.64	0.22	0.19	0.02	0.5	0.55	0.19	0.52	0.24	0.49
KY523104.1_AUL77568.1_MF405918.2_QKU33516.1_HYPOTHETICAL_PROTEIN_CDS	0.61	0.15	0.35	0.35	0.01	0.3	0.19	0.1	0.43	0.39	0.49
KY523104.1_AUL77569.1_MF405918.2_QKU33517.1_HYPOTHETICAL_PROTEIN_CDS	0.2	0.46	0.3	0.1	0.02	0.25	0.33	0.16	0.15	0.36	0.21
KY523104.1_AUL77570.1_MF405918.2_QKU33518.1_PUTATIVE_ORFAN_CDS	0.17	0.56	0.35	0.11	0	0.21	0.17	0.06	0.19	0.32	0.09
KY523104.1_AUL77571.1_MF405918.2_QKU33519.1_PUTATIVE_ORFAN_CDS	0.26	0.06	0.25	0.14	0.08	0.32	0.5	0.11	0.15	0.38	0.42
KY523104.1_AUL77572.1_MF405918.2_QKU33520.1_PUTATIVE_ORFAN_CDS	0.8	0.27	0.32	0.32	0.03	0.32	0.31	0.08	0.34	0.39	0.5
KY523104.1_AUL77573.1_MF405918.2_QKU33521.1_PUTATIVE_ORFAN_CDS	0.04	0.02	0.14	0	0	0.56	0.37	0.07	0.04	0.24	0.03
KY523104.1_AUL77574.1_MF405918.2_QKU33522.1_SERINE_PROTEINASE_INHIBITOR_CDS	0.41	0.7	0.4	0.22	0.01	0.21	0.43	0.12	0.43	0.02	0.13
KY523104.1_AUL77575.1_MF405918.2_QKU33523.1_PUTATIVE_ORFAN_CDS	0.21	0.56	0.37	0.11	0.01	0.29	0.45	0.2	0.35	0.31	0.17
KY523104.1_AUL77576.1_MF405918.2_QKU33524.1_PUTATIVE_ORFAN_CDS	0.28	0.1	0.3	0.56	0.02	0.48	0.28	0.12	0.1	0.36	0.38
KY523104.1_AUL77577.1_MF405918.2_QKU33525.1_PUTATIVE_ORFAN_CDS	0.55	0.66	0.41	0.23	0.36	0.36	0.11	0.16	0.41	0.31	0.44
KY523104.1_AUL77578.1_MF405918.2_QKU33526.1_HYPOTHETICAL_PROTEIN_CDS	0.4	0.27	0.25	0.05	0.5	0.41	0.59	0.52	0.34	0.22	0.39
KY523104.1_AUL77579.1_MF405918.2_QKU33527.1_HYPOTHETICAL_PROTEIN_CDS	0.29	0.3	0.39	0.15	0.42	0.32	0.48	0.37	0.53	0.43	0.17
KY523104.1_AUL77580.1_MF405918.2_QKU33528.1_PUTATIVE_ORFAN_CDS	0.4	0.3	0.24	0.02	0.09	0.33	0.6	0.56	0.39	0.11	0.43
KY523104.1_AUL77581.1_MF405918.2_QKU33529.1_MG457_PROTEIN_CDS	0.25	0.36	0.48	0.14	0.02	0.18	0.22	0.12	0.08	0.42	0.26
KY523104.1_AUL77582.1_MF405918.2_QKU33530.1_PUTATIVE_ORFAN_CDS	0.18	0.05	0.21	0.73	0	0.22	0.15	0.05	0.1	0.3	0.1
KY523104.1_AUL77583.1_MF405918.2_QKU33531.1_A32-LIKE_PACKAGING_ATPASE_CDS	0.39	0.28	0.3	0.29	0.47	0.43	0.34	0.22	0.47	0.52	0.43
KY523104.1_AUL77584.1_MF405918.2_QKU33532.1_PUTATIVE_F-BOX_AND_FNIP_REPEAT-CONTAINING_PROTEIN_CDS	0.22	0.55	0.43	0.18	0.02	0.37	0.25	0.18	0.41	0.32	0.2

KY523104.1_AUL77585.1_MF405918.2_QKU33533.1_GLUTAMINE_TRNA-SYNTHETASE_CDS	0.34	0.46	0.35	0.16	0.26	0.31	0.21	0.35	0.52	0.56	0.2
KY523104.1_AUL77586.1_MF405918.2_QKU33534.1_PUTATIVE_ORFAN_CDS	0.18	0.14	0.08	0	0.07	0.04	0.23	0.01	0.01	0.23	0.14
KY523104.1_AUL77587.1_MF405918.2_QKU33535.1_PUTATIVE_ORFAN_CDS	0.28	0.31	0.38	0.1	0.63	0.34	0.32	0.43	0.35	0.55	0.26
KY523104.1_AUL77588.1_MF405918.2_QKU33536.1_HYPOTHETICAL_PROTEIN_CDS	0.4	0.62	0.39	0.23	0.05	0.45	0.35	0.32	0.43	0.31	0.44
KY523104.1_AUL77589.1_MF405918.2_QKU33537.1_HYPOTHETICAL_PROTEIN_CDS	0.34	0.57	0.43	0.38	0.17	0.39	0.26	0.21	0.45	0.36	0.37
KY523104.1_AUL77590.1_MF405918.2_QKU33538.1_AAA_FAMILY_ATPASE_CDS	0.4	0.1	0.31	0.15	0.35	0.29	0.6	0.16	0.45	0.2	0.48
KY523104.1_AUL77591.1_MF405918.2_QKU33539.1_HYPOTHETICAL_PROTEIN_CDS	0.17	0.23	0.12	0.02	0.02	0.07	0.2	0.01	0.06	0.29	0.15
KY523104.1_AUL77592.1_MF405918.2_QKU33540.1_PUTATIVE_ORFAN_CDS	0.03	0.02	0.03	0	0	0.02	0.13	0	0	0.16	0.02
KY523104.1_AUL77593.1_MF405918.2_QKU33541.1_HYPOTHETICAL_PROTEIN_CDS	0.67	0.58	0.28	0.22	0.15	0.68	0.47	0.19	0.43	0.33	0.5
KY523104.1_AUL77594.1_MF405918.2_QKU33542.1_PUTATIVE_HNH_ENDONUCLEASE_CDS	0.44	0.35	0.37	0.22	0.35	0.48	0.2	0.15	0.43	0.39	0.44
KY523104.1_AUL77595.1_MF405918.2_AUL78871.2_HYPOTHETICAL_PROTEIN_CDS	0.16	0.06	0.28	0.01	0.18	0.2	0.42	0.33	0.1	0.39	0.08
KY523104.1_AUL77596.1_MF405918.2_QKU33543.1_PUTATIVE_ORFAN_CDS	0.62	0.07	0.31	0.24	0.37	0.28	0.37	0.17	0.17	0.44	0.46
KY523104.1_AUL77597.1_MF405918.2_QKU33544.1_HYPOTHETICAL_PROTEIN_CDS	0.29	0.61	0.44	0.2	0.45	0.42	0.29	0.2	0.39	0.24	0.17
KY523104.1_AUL77598.1_MF405918.2_QKU33545.1_HYPOTHETICAL_PROTEIN_CDS	0.33	0	0.19	0	0	0.03	0.52	0.75	0.21	0.09	0.22
KY523104.1_AUL77599.1_MF405918.2_QKU33546.1_PUTATIVE_OXIREDUCTASE_CDS	0.51	0.39	0.24	0.23	0.03	0.61	0.38	0.18	0.51	0.37	0.49
KY523104.1_AUL77600.1_MF405918.2_QKU33547.1_HYPOTHETICAL_PROTEIN_CDS	0.4	0.67	0.37	0.33	0.07	0.4	0.35	0.22	0.46	0.24	0.44
KY523104.1_AUL77601.1_MF405918.2_QKU33548.1_PUTATIVE_ORFAN_CDS	0.27	0.58	0.38	0.07	0.01	0.38	0.48	0.2	0.3	0.27	0.3
KY523104.1_AUL77602.1_MF405918.2_QKU33549.1_PUTATIVE_AAA+_FAMILY_ATPASE_CDS	0.45	0.48	0.32	0.26	0.28	0.63	0.2	0.08	0.49	0.43	0.45
KY523104.1_AUL77603.1_MF405918.2_QKU33550.1_HYPOTHETICAL_PROTEIN_CDS	0.14	0.55	0.37	0.04	0	0.32	0.16	0.51	0.21	0.22	0.19
KY523104.1_AUL77604.1_MF405918.2_AUL78879.2_HYPOTHETICAL_PROTEIN_CDS	0.23	0.08	0.22	0.64	0	0.14	0.06	0.04	0.11	0.38	0.14
KY523104.1_AUL77605.1_MF405918.2_AUL78880.2_PUTATIVE_ORFAN_CDS	0.1	0.06	0.07	0	0.02	0.05	0.25	0.02	0.17	0.23	0.1
KY523104.1_AUL77606.1_MF405918.2_QKU33551.1_PUTATIVE_ORFAN_CDS	0.05	0.03	0.05	0	0	0.04	0.37	0.02	0.05	0.18	0.06
KY523104.1_AUL77607.1_MF405918.2_QKU33552.1_PUTATIVE_ORFAN_CDS	0.43	0.09	0.3	0.17	0.36	0.3	0.54	0.15	0.11	0.33	0.43
KY523104.1_AUL77608.1_MF405918.2_QKU33553.1_PUTATIVE_ORFAN_CDS	0.37	0.22	0.42	0.76	0.32	0.34	0.32	0.2	0.48	0.43	0.41
KY523104.1_AUL77609.1_MF405918.2_QKU33554.1_CYTIDYLTRANSFERASERELATED_DOMAIN_CONTAINING_PROTEIN_	0.39	0.29	0.32	0.13	0.26	0.43	0.58	0.32	0.41	0.18	0.44
KY523104.1_AUL77610.1_MF405918.2_QKU33555.1_UBIQUITIN-CONJUGATING_ENZYME_E2_CDS	0.65	0.29	0.25	0.24	0.13	0.28	0.48	0.05	0.39	0.46	0.5
KY523104.1_AUL77611.1_MF405918.2_QKU33556.1_PUTATIVE_ORFAN_CDS	0.16	0.62	0.3	0.01	0	0.26	0.27	0.58	0.22	0.07	0.22
KY523104.1_AUL77612.1_MF405918.2_QKU33557.1_PUTATIVE_ORFAN_CDS	0.4	0.05	0.26	0.28	0.54	0.35	0.43	0.34	0.49	0.52	0.44
KY523104.1_AUL77613.1_MF405918.2_QKU33558.1_HYPOTHETICAL_PROTEIN_CDS	0.39	0.6	0.4	0.21	0.02	0.46	0.25	0.23	0.45	0.34	0.39
KY523104.1_AUL77614.1_MF405918.2_QKU33559.1_PUTATIVE_ORFAN_CDS	0.4	0.68	0.38	0.26	0.44	0.29	0.33	0.42	0.41	0.06	0.21
KY523104.1_AUL77615.1_MF405918.2_QKU33560.1_PUTATIVE_ORFAN_CDS	0.3	0.07	0.23	0.12	0.33	0.23	0.21	0.07	0.17	0.42	0.41
KY523104.1_AUL77616.1_MF405918.2_AUL78892.2_PUTATIVE_ORFAN_CDS	0.2	0.24	0.25	0.05	0.28	0.41	0.13	0.04	0.31	0.46	0.09
KY523104.1_AUL77617.1_MF405918.2_QKU33561.1_PUTATIVE_ORFAN_CDS	0.15	0.01	0.36	0	0.7	0.36	0.12	0.24	0.15	0.4	0.05
KY523104.1_AUL77618.1_MF405918.2_QKU33562.1_PUTATIVE_ORFAN_CDS	0.68	0.05	0.22	0.1	0.36	0.18	0.07	0.04	0.08	0.42	0.44
KY523104.1_AUL77619.1_MF405918.2_QKU33563.1_PUTATIVE_ORFAN_CDS	0.86	0.28	0.32	0.25	0.17	0.26	0.07	0.04	0.27	0.43	0.48
KY523104.1_AUL77620.1_MF405918.2_QKU33564.1_START_DOMAIN_CONTAINING_PROTEIN_CDS	0.24	0.24	0.21	0.12	0.03	0.21	0.28	0.15	0.09	0.4	0.26
KY523104.1_AUL77621.1_MF405918.2_QKU33565.1_HYPOTHETICAL_PROTEIN_CDS	0.3	0.51	0.37	0.18	0.04	0.3	0.5	0.33	0.4	0.3	0.36
KY523104.1_AUL77622.1_MF405918.2_QKU33566.1_HYPOTHETICAL_PROTEIN_CDS	0.63	0.37	0.36	0.33	0.13	0.4	0.3	0.13	0.46	0.44	0.49
KY523104.1_AUL77623.1_MF405918.2_QKU33567.1_REPEAT_PROTEIN_CDS	0.29	0.13	0.34	0.2	0.22	0.5	0.47	0.26	0.32	0.41	0.41
KY523104.1_AUL77624.1_MF405918.2_QKU33568.1_ULILYSIN_CDS	0.34	0.47	0.34	0.19	0.09	0.51	0.36	0.24	0.44	0.42	0.42
KY523104.1_AUL77625.1_MF405918.2_QKU33569.1_HYPOTHETICAL_PROTEIN_CDS	0.18	0.33	0.16	0.06	0	0.12	0.06	0.01	0.12	0.4	0.14
KY523104.1_AUL77626.1_MF405918.2_QKU33570.1_NAD-DEPENDENT_DEHYDRATASE_CDS	0.28	0.21	0.33	0.11	0.01	0.44	0.19	0.36	0.47	0.41	0.37

KY523104.1_AUL77627.1_MF405918.2_QKU33571.1_PUTATIVE_ORFAN_CDS	0.11	0.06	0.04	0	0	0.02	0.24	0	0.01	0.16	0.1
KY523104.1_AUL77626.1_MF405918.2_QKU33570.1_NAD-DEPENDENT_DEHYDRATASE_CDS	0.9	0.17	0.3	0.04	0.04	0.18	0.59	0.19	0.17	0.45	0.25
KY523104.1_AUL77628.1_MF405918.2_QKU33572.1_PUTATIVE_ORFAN_CDS	0.41	0.76	0.38	0.24	0.35	0.15	0.13	0.09	0.4	0.06	0.24
KY523104.1_AUL77629.1_MF405918.2_QKU33573.1_HYPOTHETICAL_PROTEIN_CDS	0.33	0.53	0.35	0.11	0.03	0.56	0.33	0.2	0.41	0.44	0.39
KY523104.1_AUL77630.1_MF405918.2_QKU33574.1_PUTATIVE_ORFAN_CDS	0.05	0.03	0.03	0	0	0.02	0.26	0	0.01	0.12	0.05
KY523104.1_AUL77631.1_MF405918.2_QKU33575.1_PUTATIVE_ORFAN_CDS	0.2	0.06	0.15	0.01	0.26	0.1	0.16	0.04	0.04	0.26	0.42
KY523104.1_AUL77632.1_MF405918.2_QKU33576.1_HYPOTHETICAL_PROTEIN_CDS	0.59	0.27	0.32	0.26	0.03	0.29	0.26	0.05	0.23	0.42	0.46
KY523104.1_AUL77633.1_MF405918.2_QKU33577.1_HYPOTHETICAL_PROTEIN_CDS	0.41	0.84	0.31	0.47	0.12	0.05	0.41	0.04	0.42	0.01	0.73
KY523104.1_AUL77634.1_MF405918.2_QKU33578.1_HYPOTHETICAL_PROTEIN_CDS	0.38	0.68	0.39	0.23	0	0.24	0.12	0.11	0.42	0.16	0.11
KY523104.1_AUL77635.1_MF405918.2_QKU33579.1_TRYPTOPHANYL_TRNA-SYNTHETASE_CDS	0.75	0.23	0.34	0.37	0.39	0.35	0.23	0.11	0.46	0.44	0.5
KY523104.1_AUL77636.1_MF405918.2_QKU33580.1_PUTATIVE_ORFAN_CDS	0.4	0.74	0.32	0.08	0.06	0.22	0.59	0.33	0.37	0	0.25
KY523104.1_AUL77637.1_MF405918.2_QKU33581.1_MIMIVIRUS_TRANSLATION_INITIATION_FACTOR_5A_CDS	0.26	0.21	0.26	0.14	0.02	0.3	0.44	0.14	0.13	0.4	0.33
KY523104.1_AUL77638.1_MF405918.2_QKU33582.1_PHENYLALANYL_TRNA-SYNTHETASE_CDS	0.83	0.75	0.45	0.31	0.09	0.46	0.29	0.02	0.46	0.47	0.6
KY523104.1_AUL77639.1_MF405918.2_QKU33583.1_PUTATIVE_ORFAN_CDS	0.18	0.6	0.38	0.12	0	0.23	0.13	0.08	0.23	0.29	0.13
KY523104.1_AUL77640.1_MF405918.2_QKU33584.1_PUTATIVE_ORFAN_CDS	0.29	0.52	0.33	0.18	0.01	0.31	0.31	0.11	0.42	0.38	0.39
KY523104.1_AUL77641.1_MF405918.2_QKU33585.1_PUTATIVE_ORFAN_CDS	0.82	0.29	0.44	0.32	0.17	0.34	0.28	0.27	0.44	0.29	0.54
KY523104.1_AUL77642.1_MF405918.2_QKU33586.1_HYPOTHETICAL_PROTEIN_CDS	0.21	0.3	0.33	0.08	0	0.16	0.08	0.08	0.51	0.47	0.11
KY523104.1_AUL77643.1_MF405918.2_QKU33587.1_PUTATIVE_ORFAN_CDS	0.18	0.27	0.12	0.02	0.01	0.06	0.25	0.01	0.03	0.3	0.14
KY523104.1_AUL77644.1_MF405918.2_AUL78920.2_HYPOTHETICAL_PROTEIN_CDS	0.39	0.32	0.32	0.21	0.23	0.32	0.13	0.12	0.4	0.42	0.44
KY523104.1_AUL77645.1_MF405918.2_AUL78921.2_HYPOTHETICAL_PROTEIN_CDS	0.16	0.06	0.1	0.01	0	0.11	0.1	0.01	0.45	0.36	0.16
KY523104.1_AUL77646.1_MF405918.2_QKU33588.1_PUTATIVE_ORFAN_CDS	0.35	0.36	0.37	0.23	0.43	0.33	0.49	0.27	0.3	0.38	0.43
KY523104.1_AUL77647.1_MF405918.2_QKU33589.1_HYPOTHETICAL_PROTEIN_CDS	0.74	0.28	0.43	0.05	0.15	0.52	0.38	0.77	0.38	0.03	0.47
KY523104.1_AUL77647.1_MF405918.2_QKU33589.1_HYPOTHETICAL_PROTEIN_CDS	0.2	0.08	0.34	0.04	0.55	0.4	0.56	0.28	0.23	0.31	0.11
KY523104.1_AUL77648.1_MF405918.2_QKU33590.1_PUTATIVE_ORFAN_CDS	0.2	0.08	0.34	0.04	0.55	0.4	0.56	0.28	0.23	0.31	0.11
KY523104.1_AUL77649.1_MF405918.2_QKU33591.1_STRUCTURAL_PPIASE-LIKE_PROTEIN_CDS	0.25	0.5	0.31	0.12	0.04	0.31	0.51	0.31	0.33	0.33	0.24
KY523104.1_AUL77650.1_MF405918.2_QKU33592.1_PUTATIVE_ORFAN_CDS	0.11	0.12	0.1	0.01	0	0.07	0.03	0	0.4	0.4	0.04
KY523104.1_AUL77651.1_MF405918.2_QKU33593.1_PUTATIVE_ORFAN_CDS	0.29	0.06	0.34	0.43	0.02	0.39	0.31	0.1	0.45	0.47	0.39
KY523104.1_AUL77652.1_MF405918.2_QKU33594.1_HYPOTHETICAL_PROTEIN_CDS	0.02	0.01	0.05	0	0	0.04	0	0	0	0.18	0.01
KY523104.1_AUL77653.1_MF405918.2_QKU33595.1_HYPOTHETICAL_PROTEIN_CDS	0.39	0.22	0.29	0.28	0.2	0.34	0.4	0.36	0.47	0.41	0.46
KY523104.1_AUL77654.1_MF405918.2_QKU33596.1_ANKYRIN_REPEAT_PROTEIN_CDS	0.43	0.07	0.29	0.23	0.23	0.29	0.52	0.3	0.44	0.41	0.47
KY523104.1_AUL77655.1_MF405918.2_QKU33597.1_THIOL_OXIDOREDUCTASE_E10R_CDS	0.64	0.21	0.41	0.29	0.41	0.47	0.37	0.44	0.23	0.37	0.45
KY523104.1_AUL77656.1_MF405918.2_QKU33598.1_HYPOTHETICAL_PROTEIN_CDS	0.43	0.06	0.22	0.05	0.01	0.17	0.24	0.09	0.04	0.31	0.48
KY523104.1_AUL77657.1_MF405918.2_AUL78934.2_PUTATIVE_ORFAN_CDS	0.38	0.35	0.3	0.23	0.5	0.27	0.24	0.33	0.53	0.5	0.37
KY523104.1_AUL77658.1_MF405918.2_QKU33599.1_PUTATIVE_PEPTIDASE_S9_CDS	0.19	0.26	0.28	0.06	0.11	0.25	0.33	0.21	0.19	0.42	0.15
KY523104.1_AUL77659.1_MF405918.2_QKU33600.1_PUTATIVE_ORFAN_CDS	0.39	0.65	0.42	0.22	0.25	0.32	0.32	0.26	0.42	0.17	0.27
KY523104.1_AUL77660.1_MF405918.2_QKU33601.1_HELICASE_CDS	0.38	0.23	0.29	0.14	0.01	0.32	0.55	0.46	0.43	0.36	0.46
KY523104.1_AUL77661.1_MF405918.2_QKU33602.1_MG709_PROTEIN_CDS	0.44	0.14	0.24	0.14	0.02	0.32	0.18	0.05	0.09	0.39	0.44
KY523104.1_AUL77662.1_MF405918.2_QKU33603.1_HYPOTHETICAL_PROTEIN_CDS	0.4	0.78	0.33	0.42	0.63	0.15	0.31	0.16	0.4	0.02	0.43
KY523104.1_AUL77663.1_MF405918.2_QKU33604.1_MG166_PROTEIN_CDS	0.28	0.03	0.26	0.16	0.08	0.48	0.26	0.15	0.47	0.41	0.38
KY523104.1_AUL77664.1_MF405918.2_QKU33605.1_NAD-DEPENDENT_DNA_LIGASE_CDS	0.43	0.51	0.3	0.11	0.76	0.35	0.53	0.46	0.53	0.96	0.42
KY523104.1_AUL77665.1_MF405918.2_QKU33606.1_PUTATIVE_ORFAN_CDS	0.24	0.21	0.37	0.12	0.41	0.29	0.34	0.2	0.05	0.43	0.29
KY523104.1_AUL77666.1_MF405918.2_QKU33607.1_HYPOTHETICAL_PROTEIN_CDS	0.18	0.1	0.19	0.01	0.05	0.19	0.41	0.04	0.05	0.37	0.15

KY523104.1_AUL77667.1_MF405918.2_QKU33608.1_HYPOTHETICAL_PROTEIN_CDS	0.31	0.23	0.39	0.06	0.65	0.34	0.48	0.35	0.34	0.49	0.25
KY523104.1_AUL77668.1_MF405918.2_QKU33609.1_NTP_PYROPHOSPHOHYDROLASE_CDS	0.18	0.34	0.18	0.06	0	0.13	0.09	0.01	0.38	0.44	0.15
KY523104.1_AUL77669.1_MF405918.2_QKU33610.1_PUTATIVE_ORFAN_CDS	0.17	0.06	0.12	0	0.01	0.15	0.17	0.02	0.13	0.28	0.28
KY523104.1_AUL77670.1_MF405918.2_QKU33611.1_PUTATIVE_ORFAN_CDS	0.42	0.11	0.51	0.98	0.54	0.31	0.67	0.06	0.38	0.15	0.5
KY523104.1_AUL77671.1_MF405918.2_QKU33612.1_HYPOTHETICAL_PROTEIN_CDS	0.4	0.22	0.28	0.16	0.43	0.61	0.59	0.29	0.47	0.25	0.38
KY523104.1_AUL77672.1_MF405918.2_QKU33613.1_HYPOTHETICAL_PROTEIN_CDS	0.29	0.38	0.33	0.27	0.01	0.35	0.52	0.36	0.33	0.34	0.33
KY523104.1_AUL77673.1_MF405918.2_QKU33614.1_PUTATIVE_ORFAN_CDS	0.55	0.38	0.3	0.18	0.38	0.27	0.54	0.13	0.3	0.4	0.47
KY523104.1_AUL77674.1_MF405918.2_QKU33615.1_PUTATIVE_ORFAN_CDS	0.25	0.13	0.3	0.06	0.2	0.25	0.37	0.27	0.27	0.43	0.2
KY523104.1_AUL77675.1_MF405918.2_QKU33616.1_HYPOTHETICAL_PROTEIN_CDS	0.38	0.08	0.33	0.14	0.14	0.55	0.57	0.34	0.5	0.45	0.38
KY523104.1_AUL77676.1_MF405918.2_QKU33617.1_PUTATIVE_ORFAN_CDS	0.89	0.03	0.24	0.04	0	0.2	0.02	0.08	0.02	0.31	0.51
KY523104.1_AUL77677.1_MF405918.2_QKU33618.1_HYPOTHETICAL_PROTEIN_CDS	0.35	0.32	0.34	0.13	0.02	0.28	0.56	0.39	0.39	0.28	0.43
KY523104.1_AUL77678.1_MF405918.2_QKU33619.1_PUTATIVE_ORFAN_CDS	0.24	0.77	0.37	0.14	0	0.22	0	0.07	0.42	0.23	0.07
KY523104.1_AUL77679.1_MF405918.2_QKU33620.1_PUTATIVE_ORFAN_CDS	0.16	0.21	0.33	0.03	0.03	0.53	0.02	0.56	0.1	0.13	0.13
KY523104.1_AUL77680.1_MF405918.2_QKU33621.1_PUTATIVE_N-ACETYL_TRANSFERASE_CDS	0.4	0.08	0.3	0.23	0.63	0.52	0.5	0.68	0.32	0.6	0.43
KY523104.1_AUL77681.1_MF405918.2_QKU33622.1_PUTATIVE_ORFAN_CDS	0.21	0.35	0.28	0.12	0.31	0.23	0.28	0.1	0.14	0.41	0.13
KY523104.1_AUL77682.1_MF405918.2_QKU33623.1_HYPOTHETICAL_PROTEIN_CDS	0.21	0.34	0.4	0.11	0	0.35	0.4	0.27	0.34	0.4	0.15
KY523104.1_AUL77683.1_MF405918.2_QKU33624.1_PUTATIVE_ATP-DEPENDENT_HELICASE_CDS	0.4	0.63	0.42	0.54	0.42	0.56	0.45	0.26	0.45	0.43	0.44
KY523104.1_AUL77684.1_MF405918.2_QKU33625.1_HYPOTHETICAL_PROTEIN_CDS	0.24	0.09	0.36	0.03	0.67	0.33	0.37	0.47	0.14	0.41	0.19
KY523104.1_AUL77685.1_MF405918.2_QKU33626.1_ANKYRIN_REPEAT_PROTEIN_CDS	0.4	0.62	0.4	0.04	0.14	0.37	0.32	0.6	0.38	0.07	0.38
KY523104.1_AUL77686.1_MF405918.2_QKU33627.1_PUTATIVE_ORFAN_CDS	0.4	0.16	0.31	0.17	0.04	0.27	0.27	0.48	0.1	0.36	0.43
KY523104.1_AUL77687.1_MF405918.2_QKU33628.1_PUTATIVE_HELICASE_CDS	0.48	0.45	0.25	0.29	0.21	0.4	0.38	0.06	0.5	0.42	0.49
KY523104.1_AUL77688.1_MF405918.2_QKU33629.1_HYPOTHETICAL_PROTEIN_CDS	0.24	0.31	0.39	0.28	0.44	0.56	0.43	0.33	0.29	0.45	0.17
KY523104.1_AUL77689.1_MF405918.2_QKU33630.1_PUTATIVE_PATATIN-LIKE_PHOSPHOLIPASE_CDS	0.38	0.59	0.41	0.58	0.04	0.38	0.34	0.4	0.47	0.33	0.38
KY523104.1_AUL77690.1_MF405918.2_QKU33631.1_PUTATIVE_ORFAN_CDS	0.44	0.7	0.24	0.31	0.2	0.57	0.51	0.33	0.56	0.31	0.49
KY523104.1_AUL77691.1_MF405918.2_QKU33632.1_HYPOTHETICAL_PROTEIN_CDS	0.39	0.06	0.28	0	0	0.43	0.5	0.76	0.32	0.06	0.22
KY523104.1_AUL77692.1_MF405918.2_QKU33633.1_HYPOTHETICAL_PROTEIN_CDS	0.58	0.55	0.42	0.16	0.09	0.5	0.26	0.43	0.36	0.3	0.45
KY523104.1_AUL77693.1_MF405918.2_QKU33634.1_PUTATIVE_ATP-DEPENDENT_DNA_HELICASE_CDS	0.44	0.23	0.19	0.09	0.1	0.4	0.6	0.67	0.55	0.26	0.5
KY523104.1_AUL77694.1_MF405918.2_QKU33635.1_HYPOTHETICAL_PROTEIN_CDS	0.39	0.3	0.37	0.17	0.55	0.42	0.18	0.36	0.52	0.66	0.33
KY523104.1_AUL77695.1_MF405918.2_QKU33636.1_PUTATIVE_ORFAN_CDS	0.24	0.31	0.31	0.09	0.08	0.49	0.22	0.05	0.26	0.48	0.2
KY523104.1_AUL77696.1_MF405918.2_QKU33637.1_OXIDOREDUCTASE_FAD-BINDING_CDS	0.4	0.72	0.43	0.74	0.57	0.52	0.37	0.26	0.55	0.32	0.29
KY523104.1_AUL77697.1_MF405918.2_QKU33638.1_HYPOTHETICAL_PROTEIN_CDS	0.27	0.23	0.25	0.13	0.05	0.27	0.4	0.07	0.05	0.41	0.37
KY523104.1_AUL77698.1_MF405918.2_QKU33639.1_HYPOTHETICAL_PROTEIN_CDS	0.97	0.04	0.22	0.04	0	0.18	0.01	0.05	0.02	0.32	0.51
KY523104.1_AUL77699.1_MF405918.2_QKU33640.1_HYPOTHETICAL_PROTEIN_CDS	0.93	0.1	0.24	0.31	0.02	0.32	0.18	0.06	0.44	0.3	0.72
KY523104.1_AUL77700.1_MF405918.2_QKU33641.1_MG366_PROTEIN_CDS	0.4	0.33	0.26	0.12	0.17	0.24	0.58	0.32	0.42	0.18	0.47
KY523104.1_AUL77701.1_MF405918.2_QKU33642.1_GLUTAMINE_SYNTHETASE_CDS	0.4	0.07	0.37	0	0	0.67	0.49	0.75	0.38	0.16	0.36
KY523104.1_AUL77702.1_MF405918.2_QKU33643.1_PUTATIVE_ORFAN_CDS	0.23	0.15	0.14	0.02	0.02	0.14	0.14	0.08	0.11	0.36	0.28
KY523104.1_AUL77703.1_MF405918.2_QKU33644.1_ALANYL-TRNA_SYNTHETASE_CDS	0.31	0.4	0.35	0.17	0.2	0.48	0.26	0.2	0.45	0.42	0.4
KY523104.1_AUL77704.1_MF405918.2_QKU33645.1_PUTATIVE_ORFAN_CDS	0.24	0.19	0.32	0.05	0.53	0.41	0.13	0.14	0.16	0.46	0.19
KY523104.1_AUL77705.1_MF405918.2_QKU33646.1_PUTATIVE_ORFAN_CDS	0.3	0.07	0.18	0.02	0.01	0.14	0.19	0.02	0.02	0.35	0.45
KY523104.1_AUL77706.1_MF405918.2_QKU33647.1_PUTATIVE_ORFAN_CDS	0.01	0.02	0.13	0	0	0.53	0	0	0.05	0.25	0.01
KY523104.1_AUL77707.1_MF405918.2_QKU33648.1_PUTATIVE_ATP-DEPENDENT_RNA_HELICASE_CDS	0.43	0.45	0.4	0.23	0.09	0.41	0.43	0.19	0.31	0.4	0.45
KY523104.1_AUL77708.1_MF405918.2_QKU33649.1_HYPOTHETICAL_PROTEIN_CDS	0.26	0.17	0.38	0.6	0.01	0.49	0.34	0.2	0.14	0.39	0.24

KY523104.1_AUL77709.1_MF405918.2_AUL78989.2_PUTATIVE_ORFAN_CDS	0.34	0.63	0.42	0.24	0.19	0.28	0.35	0.22	0.53	0.41	0.13
KY523104.1_AUL77710.1_MF405918.2_QKU33650.1_HYPOTHETICAL_PROTEIN_CDS	0.4	0.56	0.41	0.33	0.51	0.42	0.35	0.31	0.46	0.43	0.4
KY523104.1_AUL77711.1_MF405918.2_QKU33651.1_GLYCOSYL_HYDROLASE_FAMILY_18_CDS	0.8	0.48	0.08	0.21	0.09	0.17	0.5	0	0.48	0.38	0.78
KY523104.1_AUL77712.1_MF405918.2_QKU33652.1_HYPOTHETICAL_PROTEIN_CDS	0.4	0.4	0.35	0.21	0	0.47	0.15	0.13	0.52	0.35	0.43
KY523104.1_AUL77713.1_MF405918.2_QKU33653.1_ABC_TRANSPORTER_ATP-BINDING_PROTEIN_PERMEASE_CDS	0.4	0.05	0.22	0	0	0.31	0.61	0.32	0.35	0.09	0.24
KY523104.1_AUL77714.1_MF405918.2_QKU33654.1_MG680_PROTEIN_CDS	0.23	0.57	0.4	0.22	0.01	0.28	0.05	0.09	0.47	0.38	0.18
KY523104.1_AUL77715.1_MF405918.2_QKU33655.1_PUTATIVE_ORFAN_CDS	0.58	0.07	0.56	0.32	0.11	0.36	0.45	0.08	0.37	0.36	0.48
KY523104.1_AUL77716.1_MF405918.2_QKU33656.1_HELIX-TURN-HELIX_XRE-FAMILY_LIKE_PROTEIN_CDS	0.4	0.75	0.5	0.42	0.68	0.34	0.03	0.21	0.39	0.23	0.29
KY523104.1_AUL77717.1_MF405918.2_QKU33657.1_HYPOTHETICAL_PROTEIN_CDS	0.39	0.6	0.42	0.42	0.41	0.32	0.29	0.23	0.53	0.4	0.33
KY523104.1_AUL77718.1_MF405918.2_QKU33658.1_HYPOTHETICAL_PROTEIN_CDS	0.36	0.56	0.36	0.19	0.28	0.55	0.26	0.2	0.48	0.52	0.36
KY523104.1_AUL77719.1_MF405918.2_QKU33659.1_PUTATIVE_ORFAN_CDS	0.72	0.15	0.18	0.1	0	0.71	0.23	0.11	0.47	0.31	0.49
KY523104.1_AUL77720.1_MF405918.2_QKU33660.1_PUTATIVE_ORFAN_CDS	0.36	0.08	0.39	0	0.1	0.22	0.1	0.71	0.51	0.36	0.27
KY523104.1_AUL77721.1_MF405918.2_QKU33661.1_PUTATIVE_DNA_REPAIR_PROTEIN_CDS	0.54	0.66	0.26	0.27	0.63	0.6	0.46	0.6	0.49	0.15	0.54
KY523104.1_AUL77722.1_MF405918.2_QKU33662.1_HYPOTHETICAL_PROTEIN_CDS	0.28	0.05	0.37	0.01	0.44	0.26	0.3	0.47	0.5	0.39	0.15
KY523104.1_AUL77723.1_MF405918.2_QKU33663.1_HYPOTHETICAL_PROTEIN_CDS	0.41	0.29	0.25	0.25	0.44	0.25	0.53	0.31	0.48	0.44	0.48
KY523104.1_AUL77724.1_MF405918.2_QKU33664.1_PUTATIVE_ORFAN_CDS	0.4	0.67	0.45	0.06	0.45	0.38	0.55	0.55	0.4	0.07	0.29
KY523104.1_AUL77725.1_MF405918.2_QKU33665.1_MG347_PROTEIN_CDS	0.48	0.09	0.22	0.07	0.01	0.17	0.14	0.07	0.04	0.33	0.48
KY523104.1_AUL77726.1_MF405918.2_QKU33666.1_HYPOTHETICAL_PROTEIN_CDS	0.86	0.12	0.44	0.28	0.77	0.12	0.33	0.12	0.39	0.51	0.58
KY523104.1_AUL77727.1_MF405918.2_QKU33667.1_ANKYRIN_REPEAT_PROTEIN_CDS	0.36	0.28	0.31	0.26	0	0.49	0.51	0.07	0.39	0.41	0.46
KY523104.1_AUL77728.1_MF405918.2_QKU33668.1_PUTATIVE_ORFAN_CDS	0.3	0.1	0.31	0.23	0.41	0.33	0.45	0.16	0.37	0.44	0.42
KY523104.1_AUL77729.1_MF405918.2_QKU33669.1_PUTATIVE_ORFAN_CDS	0.5	0.35	0.33	0.21	0.46	0.35	0.2	0.2	0.28	0.44	0.44
KY523104.1_AUL77730.1_MF405918.2_QKU33670.1_PUTATIVE_ORFAN_CDS	0.39	0.55	0.33	0.01	0	0.43	0.43	0.61	0.35	0.04	0.37
KY523104.1_AUL77731.1_MF405918.2_QKU33671.1_PUTATIVE_ORFAN_CDS	0.2	0.33	0.2	0.08	0.01	0.13	0.24	0.03	0.1	0.38	0.19
KY523104.1_AUL77732.1_MF405918.2_QKU33672.1_PUTATIVE_ORFAN_CDS	0.28	0.51	0.41	0.19	0.35	0.32	0.37	0.24	0.45	0.38	0.27
KY523104.1_AUL77733.1_MF405918.2_QKU33673.1_PUTATIVE_ORFAN_CDS	0.14	0.26	0.12	0.03	0.02	0.07	0.33	0.02	0.05	0.26	0.14
KY523104.1_AUL77734.1_MF405918.2_QKU33674.1_PUTATIVE_ORFAN_CDS	0.12	0.48	0.25	0.05	0	0.14	0.12	0.08	0.02	0.29	0.14
KY523104.1_AUL77735.1_MF405918.2_QKU33675.1_AAA_ATPASE_DOMAIN-CONTAINING_PROTEIN_CDS	0.33	0.31	0.29	0.18	0.32	0.25	0.54	0.35	0.32	0.31	0.43
KY523104.1_AUL77736.1_MF405918.2_QKU33676.1_PUTATIVE_ORFAN_CDS	0.32	0.18	0.19	0.06	0.23	0.15	0.09	0.05	0.04	0.34	0.43
KY523104.1_AUL77737.1_MF405918.2_QKU33677.1_PUTATIVE_ORFAN_CDS	0.19	0.12	0.12	0.02	0.01	0.14	0.21	0.02	0.41	0.36	0.16
KY523104.1_AUL77738.1_MF405918.2_QKU33678.1_PHOSPHOLIPID-TRANSPORTING_P-TYPE_ATPASE_CDS	0.39	0.43	0.4	0.05	0	0.6	0.59	0.51	0.51	0.22	0.32
KY523104.1_AUL77739.1_MF405918.2_QKU33679.1_PUTATIVE_ORFAN_CDS	0.35	0.09	0.16	0.02	0.06	0.13	0.11	0.04	0.03	0.28	0.5
KY523104.1_AUL77740.1_MF405918.2_QKU33680.1_HYPOTHETICAL_PROTEIN_CDS	0.4	0.53	0.5	0.06	0.71	0.22	0.43	0.49	0.46	0.85	0.36
KY523104.1_AUL77741.1_MF405918.2_QKU33681.1_PUTATIVE_ORFAN_CDS	0.36	0.24	0.33	0.16	0.02	0.45	0.27	0.09	0.18	0.4	0.43
KY523104.1_AUL77742.1_MF405918.2_QKU33682.1_PUTATIVE_ORFAN_CDS	0.09	0.02	0.11	0	0.02	0.08	0.21	0.03	0.34	0.23	0.28
KY523104.1_AUL77743.1_MF405918.2_QKU33683.1_HYPOTHETICAL_PROTEIN_CDS	0.36	0.59	0.38	0.12	0.41	0.38	0.27	0.51	0.44	0.29	0.29
KY523104.1_AUL77744.1_MF405918.2_QKU33684.1_PUTATIVE_LYSOCARDIOLIPIN_ACYLTRANSFERASE_1-LIKE_PROTEIN	0.23	0.35	0.36	0.17	0.02	0.49	0.37	0.15	0.3	0.47	0.17
KY523104.1_AUL77745.1_MF405918.2_QKU33685.1_PUTATIVE_ORFAN_CDS	0.68	0.19	0.29	0.3	0.01	0.25	0.09	0.07	0.27	0.39	0.48
KY523104.1_AUL77746.1_MF405918.2_QKU33686.1_HYPOTHETICAL_PROTEIN_CDS	0.23	0.03	0.16	0.01	0	0.46	0.26	0.04	0.05	0.31	0.2
KY523104.1_AUL77747.1_MF405918.2_QKU33687.1_PUTATIVE_ORFAN_CDS	0.33	0.48	0.37	0.22	0	0.44	0.18	0.35	0.47	0.41	0.38
KY523104.1_AUL77748.1_MF405918.2_QKU33688.1_HISTONE_DEACETYLASE_CDS	0.59	0.5	0.19	0	0	0.99	0.16	0.08	0.6	0.11	0.61
KY523104.1_AUL77749.1_MF405918.2_QKU33689.1_THIOREDOXIN-LIKE_PROTEIN_CDS	0.18	0.26	0.16	0.03	0	0.17	0.26	0.02	0.18	0.4	0.13
KY523104.1_AUL77750.1_MF405918.2_QKU33690.1_HYPOTHETICAL_PROTEIN_CDS	0.3	0.28	0.29	0.03	0	0.46	0.57	0.42	0.39	0.24	0.18

KY523104.1_AUL77751.1_MF405918.2_QKU33691.1_PUTATIVE_ORFAN_CDS	0.41	0.44	0.23	0.02	0.35	0.11	0.65	0.12	0.38	0.01	0.48
KY523104.1_AUL77752.1_MF405918.2_QKU33692.1_HYPOTHETICAL_PROTEIN_CDS	0.21	0.23	0.25	0	0	0.34	0.58	0.17	0.27	0.25	0.11
KY523104.1_AUL77753.1_MF405918.2_QKU33693.1_HYPOTHETICAL_PROTEIN_CDS	0.55	0.19	0.27	0.26	0.08	0.29	0.34	0.1	0.34	0.43	0.46
KY523104.1_AUL77754.1_MF405918.2_QKU33694.1_HYPOTHETICAL_PROTEIN_CDS	0.24	0.11	0.36	0.3	0.01	0.31	0.41	0.15	0.42	0.42	0.18
KY523104.1_AUL77755.1_MF405918.2_QKU33695.1_MG661_PROTEIN_CDS	0.4	0.65	0.4	0.21	0.19	0.58	0.41	0.3	0.46	0.24	0.37
KY523104.1_AUL77756.1_MF405918.2_QKU33696.1_HYPOTHETICAL_PROTEIN_CDS	0.95	0.48	0.12	0.52	0.22	0.32	0.52	0.01	0.57	0.59	0.9
KY523104.1_AUL77757.1_MF405918.2_QKU33697.1_PUTATIVE_ORFAN_CDS	0.14	0.06	0.23	0.01	0.05	0.16	0.38	0.28	0.01	0.35	0.19
KY523104.1_AUL77758.1_MF405918.2_QKU33698.1_PUTATIVE_ATP-DEPENDENT_RNA_HELICASE_CDS	0.4	0.44	0.27	0.15	0.07	0.3	0.56	0.19	0.47	0.24	0.47
KY523104.1_AUL77759.1_MF405918.2_QKU33699.1_ALKALINE_PHOSPHATASE_CDS	0.43	0.3	0.3	0.55	0.47	0.38	0.45	0.17	0.41	0.5	0.47
KY523104.1_AUL77760.1_MF405918.2_QKU33700.1_PUTATIVE_ORFAN_CDS	0.27	0.17	0.16	0.03	0.01	0.15	0.13	0.02	0.04	0.38	0.38
KY523104.1_AUL77761.1_MF405918.2_QKU33701.1_NCPA_CDS	0.68	0.64	0.19	0.18	0.4	0.59	0.53	0.21	0.51	0.29	0.61
KY523104.1_AUL77762.1_MF405918.2_QKU33702.1_PUTATIVE_CYTOCHROME_P450_1A1-LIKE_ISOFORM_X2_CDS	0.41	0.08	0.3	0	0.04	0.28	0.11	0.48	0.57	0.23	0.47
KY523104.1_AUL77763.1_MF405918.2_QKU33703.1_PUTATIVE_ORFAN_CDS	0.19	0.15	0.23	0	0.81	0.21	0.23	0.08	0.03	0.38	0.05
KY523104.1_AUL77764.1_MF405918.2_QKU33704.1_PUTATIVE_ORFAN_CDS	0.29	0.22	0.2	0.08	0.01	0.34	0.14	0.04	0.05	0.38	0.4
KY523104.1_AUL77765.1_MF405918.2_QKU33705.1_PUTATIVE_ORFAN_CDS	0.34	0.32	0.38	0.14	0.56	0.39	0.23	0.3	0.34	0.55	0.37
KY523104.1_AUL77766.1_MF405918.2_QKU33706.1_PUTATIVE_ORFAN_CDS	0.18	0.33	0.18	0.04	0.01	0.15	0.53	0.08	0.04	0.34	0.11
KY523104.1_AUL77767.1_MF405918.2_QKU33707.1_RNA_LIGASE_2_CDS	0.41	0.66	0.43	0.28	0.05	0.47	0.23	0.15	0.46	0.37	0.47
KY523104.1_AUL77768.1_MF405918.2_QKU33708.1_PUTATIVE_ORFAN_CDS	0.25	0.35	0.37	0.3	0.02	0.5	0.44	0.2	0.27	0.44	0.27
KY523104.1_AUL77769.1_MF405918.2_QKU33709.1_PUTATIVE_ORFAN_CDS	0.24	0.39	0.38	0.49	0.38	0.31	0.23	0.24	0.26	0.41	0.22
KY523104.1_AUL77770.1_MF405918.2_QKU33710.1_HYPOTHETICAL_PROTEIN_CDS	0.51	0.16	0.33	0.29	0.4	0.36	0.26	0.25	0.39	0.42	0.45
KY523104.1_AUL77771.1_MF405918.2_QKU33711.1_PUTATIVE_FE2OG_OXYGENASE_FAMILY_OXIDOREDUCTASE_CDS	0.29	0.22	0.34	0.02	0.3	0.21	0.22	0.16	0.52	0.41	0.1
KY523104.1_AUL77772.1_MF405918.2_QKU33712.1_HYPOTHETICAL_PROTEIN_CDS	0.25	0.13	0.23	0.01	0.59	1	0.07	0.39	0.39	0.05	0.14
KY523104.1_AUL77773.1_MF405918.2_QKU33713.1_ATP-DEPENDENT_RNA_HELICASE_CDS	0.38	0.5	0.24	0.04	0	0.21	0.59	0.25	0.45	0.08	0.35
KY523104.1_AUL77774.1_MF405918.2_QKU33714.1_HYPOTHETICAL_PROTEIN_CDS	0.63	0.35	0.36	0.32	0.54	0.37	0.33	0.27	0.39	0.48	0.46
KY523104.1_AUL77775.1_MF405918.2_QKU33715.1_PUTATIVE_MEMBRANE_PROTEIN_AT3G27390_CDS	0.37	0.34	0.33	0.3	0.45	0.41	0.39	0.24	0.45	0.48	0.42
KY523104.1_AUL77776.1_MF405918.2_QKU33716.1_PUTATIVE_ORFAN_CDS	0.41	0.72	0.4	0.42	0.59	0.35	0.32	0.37	0.4	0.04	0.25
KY523104.1_AUL77777.1_MF405918.2_QKU33717.1_PUTATIVE_ORFAN_CDS	0.33	0.29	0.32	0.2	0	0.45	0.52	0.4	0.49	0.39	0.39
KY523104.1_AUL77778.1_MF405918.2_QKU33718.1_PUTATIVE_ORFAN_CDS	0.11	0.06	0.06	0	0	0.04	0.23	0.01	0.02	0.17	0.35
KY523104.1_AUL77779.1_MF405918.2_QKU33719.1_PUTATIVE_ORFAN_CDS	0.4	0.64	0.34	0.2	0.01	0.55	0.41	0.32	0.44	0.27	0.44
KY523104.1_AUL77780.1_MF405918.2_QKU33720.1_ANKYRIN_REPEAT_DOMAIN_CONTAINING_PROTEIN_CDS	0.25	0.04	0.31	0.04	0.36	0.29	0.44	0.47	0.09	0.4	0.25
KY523104.1_AUL77781.1_MF405918.2_QKU33721.1_LYSINE-TRNA_SYNTHETASE_CDS	0.36	0.54	0.46	0.1	0.32	0.38	0.46	0.2	0.49	0.65	0.29
KY523104.1_AUL77782.1_MF405918.2_QKU33722.1_HYPOTHETICAL_PROTEIN_CDS	0.3	0.35	0.32	0.08	0.1	0.31	0.49	0.54	0.3	0.36	0.35
KY523104.1_AUL77783.1_MF405918.2_QKU33723.1_PUTATIVE_ORFAN_CDS	0.41	0.16	0.31	0.22	0.01	0.43	0.34	0.24	0.52	0.48	0.46
KY523104.1_AUL77784.1_MF405918.2_QKU33724.1_HYPOTHETICAL_PROTEIN_CDS	0.26	0.32	0.33	0.16	0.01	0.35	0.43	0.1	0.2	0.39	0.38
KY523104.1_AUL77785.1_MF405918.2_QKU33725.1_HYPOTHETICAL_PROTEIN_CDS	0.28	0.2	0.19	0.09	0.24	0.26	0.1	0.07	0.09	0.38	0.35
KY523104.1_AUL77786.1_MF405918.2_QKU33726.1_HYPOTHETICAL_PROTEIN_CDS	0.4	0.21	0.37	0.03	0.61	0.5	0.47	0.36	0.37	0.63	0.36
KY523104.1_AUL77787.1_MF405918.2_QKU33727.1_HYPOTHETICAL_PROTEIN_CDS	0.41	0.63	0.31	0.34	0.01	0.37	0.41	0.15	0.52	0.39	0.46
KY523104.1_AUL77788.1_MF405918.2_QKU33728.1_HYDROLASE_CDS	0.24	0.27	0.3	0.12	0	0.46	0.39	0.11	0.2	0.45	0.25
KY523104.1_AUL77789.1_MF405918.2_QKU33729.1_HYPOTHETICAL_PROTEIN_CDS	0.39	0.33	0.38	0.05	0	0.43	0.59	0.21	0.45	0.56	0.35
KY523104.1_AUL77790.1_MF405918.2_QKU33730.1_CYTOCHROME_450-LIKE_PROTEIN_CDS	0.56	0.5	0.35	0.29	0.18	0.41	0.34	0.15	0.44	0.41	0.47
KY523104.1_AUL77791.1_MF405918.2_QKU33731.1_PUTATIVE_TRIACYLGLYCEROL_LIPASE_CDS	0.24	0.3	0.43	0.12	0.01	0.22	0.18	0.02	0.05	0.41	0.28
KY523104.1_AUL77792.1_MF405918.2_QKU33732.1_PUTATIVE_DNA_HELICASE_CDS	0.38	0.46	0.22	0.04	0.1	0.37	0.59	0.27	0.4	0.1	0.31

KY523104.1_AUL77793.1_MF405918.2_QKU33733.1_PUTATIVE_ORFAN_CDS	0.25	0.29	0.3	0.21	0.01	0.39	0.38	0.12	0.25	0.44	0.26
KY523104.1_AUL77794.1_MF405918.2_QKU33734.1_MIMIVIRUS_TRANSLATION_INITIATION_FACTOR_4E,_1_CDS	0.27	0.2	0.29	0.19	0.13	0.3	0.47	0.12	0.48	0.38	0.35
KY523104.1_AUL77795.1_MF405918.2_QKU33735.1_PUTATIVE_5'-3'EXONUCLEASE20_CDS	0.58	0.63	0.34	0.35	0.19	0.45	0.29	0.13	0.48	0.37	0.49
KY523104.1_AUL77796.1_MF405918.2_QKU33736.1_PHOSPHOESTERASE-LIKE_PROTEIN_CDS	0.4	0.65	0.46	0.12	0.38	0.56	0.48	0.37	0.48	0.24	0.34
KY523104.1_AUL77797.1_MF405918.2_QKU33737.1_PUTATIVE_5'-3'_EXONUCLEASE_20_CDS	0.39	0.15	0.35	0.15	0.24	0.31	0.58	0.32	0.48	0.3	0.42
KY523104.1_AUL77798.1_MF405918.2_QKU33738.1_HYPOTHETICAL_PROTEIN_CDS	0.4	0.37	0.28	0.33	0.55	0.35	0.44	0.3	0.61	0.6	0.42
KY523104.1_AUL77799.1_MF405918.2_QKU33739.1_PUTATIVE_DIVERGENT_NUDIX_HYDROLASE_CDS	0.27	0.25	0.27	0.15	0.01	0.28	0.18	0.11	0.47	0.43	0.34
KY523104.1_AUL77800.1_MF405918.2_QKU33740.1_MG642_PROTEIN_CDS	0.19	0.35	0.26	0.07	0	0.13	0.17	0.01	0.05	0.36	0.22
KY523104.1_AUL77801.1_MF405918.2_QKU33741.1_DHH_FAMILY_PHOSPHOHYDROLASE-LIKE_PROTEIN_CDS	0.39	0.67	0.42	0.34	0.3	0.3	0.37	0.46	0.55	0.31	0.18
KY523104.1_AUL77802.1_MF405918.2_QKU33742.1_UBIQUITIN-CONJUGATING_ENZYME_E2_CDS	0.5	0.58	0.11	0.16	0.66	0.76	0.62	0.39	0.67	0.73	0.54
KY523104.1_AUL77803.1_MF405918.2_QKU33743.1_MG639_PROTEIN_CDS	0.4	0.68	0.41	0.14	0.2	0.74	0.52	0.41	0.49	0.15	0.37
KY523104.1_AUL77804.1_MF405918.2_QKU33744.1_PUTATIVE_ORFAN_CDS	0.14	0.12	0.07	0	0.01	0.04	0.32	0.02	0.03	0.22	0.1
KY523104.1_AUL77805.1_MF405918.2_QKU33745.1_PUTATIVE_ZINC_METALLOPROTEINASE_CDS	0.4	0.63	0.42	0.57	0.28	0.47	0.35	0.37	0.49	0.37	0.36
KY523104.1_AUL77806.1_MF405918.2_QKU33746.1_HYPOTHETICAL_PROTEIN_CDS	0.4	0.6	0.22	0.21	0	0.61	0.44	0.12	0.53	0.47	0.44
KY523104.1_AUL77807.1_MF405918.2_QKU33747.1_HYPOTHETICAL_PROTEIN_CDS	0.23	0.34	0.34	0.07	0.07	0.48	0.22	0.19	0.21	0.44	0.17
KY523104.1_AUL77808.1_MF405918.2_QKU33748.1_HYPOTHETICAL_PROTEIN_CDS	0.29	0.57	0.38	0.13	0.19	0.33	0.48	0.31	0.33	0.29	0.32
KY523104.1_AUL77809.1_MF405918.2_QKU33749.1_PUTATIVE_SERINE_THREONINE-PROTEIN_KINASE_CDS	0.4	0.44	0.25	0.13	0.05	0.26	0.61	0.37	0.61	0.2	0.4
KY523104.1_AUL77810.1_MF405918.2_QKU33750.1_HYPOTHETICAL_PROTEIN_CDS	0.22	0.47	0.37	0.18	0.01	0.28	0.15	0.11	0.47	0.4	0.16
KY523104.1_AUL77811.1_MF405918.2_QKU33751.1_PUTATIVE_ORFAN_CDS	0.53	0.18	0.3	0.15	0.01	0.61	0.51	0.11	0.38	0.33	0.48
KY523104.1_AUL77812.1_MF405918.2_QKU33752.1_HYPOTHETICAL_PROTEIN_CDS	0.14	0.28	0.22	0.02	0	0.5	0.37	0.03	0.04	0.34	0.08
KY523104.1_AUL77813.1_MF405918.2_QKU33753.1_HYPOTHETICAL_PROTEIN_CDS	0.29	0.12	0.31	0.29	0.47	0.31	0.35	0.14	0.08	0.45	0.41
KY523104.1_AUL77814.1_MF405918.2_QKU33754.1_HYPOTHETICAL_PROTEIN_CDS	0.23	0.09	0.14	0.04	0.01	0.14	0.11	0.04	0.04	0.29	0.4
KY523104.1_AUL77815.1_MF405918.2_QKU33755.1_MIMIVIRUS_TRANSLATION_INITIATION_FACTOR_5_CDS	0.91	0.03	0.25	0.28	0	0.31	0.19	0.09	0.39	0.28	0.67
KY523104.1_AUL77816.1_MF405918.2_QKU33756.1_HYPOTHETICAL_PROTEIN_CDS	0.46	0.3	0.31	0.22	0.32	0.3	0.12	0.09	0.37	0.44	0.44
KY523104.1_AUL77817.1_MF405918.2_QKU33757.1_PUTATIVE_DEOXYNUCLEOTIDE_MONOPHOSPHATE_KINASE_CDS	0.4	0.22	0.23	0.06	0.03	0.27	0.59	0.45	0.41	0.14	0.44
KY523104.1_AUL77818.1_MF405918.2_QKU33758.1_PUTATIVE_FTSJ-LIKE_METHYLTRANSFERASE_CDS	0.41	0.48	0.12	0	0.6	0.15	0.72	0.32	0.35	0	0.4
KY523104.1_AUL77819.1_MF405918.2_QKU33759.1_PUTATIVE_ORFAN_CDS	0.4	0.28	0.29	0.05	0.26	0.41	0.63	0.15	0.47	0.44	0.42
KY523104.1_AUL77820.1_MF405918.2_QKU33760.1_HYPOTHETICAL_PROTEIN_CDS	0.4	0.49	0.36	0.13	0.48	0.78	0.57	0.45	0.42	0.57	0.44
KY523104.1_AUL77821.1_MF405918.2_QKU33761.1_PUTATIVE_REPLICATION_FACTOR_C_SMALL_SUBUNIT_CDS	0.37	0.08	0.36	0	0	0.31	0.58	0.22	0.46	0.26	0.14
KY523104.1_AUL77822.1_MF405918.2_QKU33762.1_HYPOTHETICAL_PROTEIN_CDS	0.44	0.47	0.06	0	0.02	0.18	0.7	0.13	0.37	0	0.58
KY523104.1_AUL77823.1_MF405918.2_QKU33763.1_METALLOPHOSPHOESTERASE-LIKE_PROTEIN_CDS	0.32	0.25	0.4	0.39	0.03	0.45	0.12	0.16	0.42	0.4	0.42
KY523104.1_AUL77824.1_MF405918.2_QKU33764.1_PUTATIVE_B-TYPE_LECTIN_PROTEIN_CDS	0.6	0.72	0.32	0.29	0.32	0.54	0.45	0.18	0.42	0.2	0.52
KY523104.1_AUL77825.1_MF405918.2_QKU33765.1_PUTATIVE_ORFAN_CDS	0.5	0.2	0.31	0.28	0.36	0.3	0.37	0.12	0.22	0.45	0.45
KY523104.1_AUL77826.1_MF405918.2_QKU33766.1_HYPOTHETICAL_PROTEIN_CDS	0.19	0.21	0.34	0.05	0.17	0.25	0.27	0.18	0.19	0.44	0.13
KY523104.1_AUL77827.1_MF405918.2_QKU33767.1_PUTATIVE_ORFAN_CDS	0.43	0.68	0.35	0.26	0.32	0.57	0.29	0.21	0.46	0.27	0.45
KY523104.1_AUL77828.1_MF405918.2_QKU33768.1_TRANSPOSASE_CDS	0.17	0.34	0.15	0.03	0	0.14	0.22	0.01	0.01	0.33	0.12
KY523104.1_AUL77829.1_MF405918.2_QKU33769.1_PUTATIVE_ATP-DEPENDENT_RNA_HELICASE_CDS	0.4	0.25	0.19	0	0.01	0.35	0.6	0.74	0.36	0.32	0.41
KY523104.1_AUL77830.1_MF405918.2_QKU33770.1_PUTATIVE_ORFAN_CDS	0.29	0.11	0.33	0.57	0.41	0.34	0.16	0.13	0.27	0.41	0.33
KY523104.1_AUL77831.1_MF405918.2_QKU33771.1_HYPOTHETICAL_PROTEIN_CDS	0.3	0.29	0.36	0.55	0.27	0.34	0.27	0.14	0.15	0.41	0.41
KY523104.1_AUL77832.1_MF405918.2_QKU33772.1_HYPOTHETICAL_PROTEIN_CDS	0.31	0.33	0.38	0.62	0.01	0.32	0.17	0.15	0.5	0.46	0.24
KY523104.1_AUL77833.1_MF405918.2_QKU33773.1_HYPOTHETICAL_PROTEIN_CDS	0.4	0.09	0.85	0.61	0.37	0.65	0.32	0.44	0.48	0.51	0.42
KY523104.1_AUL77834.1_MF405918.2_QKU33774.1_HYPOTHETICAL_PROTEIN_CDS	0.4	0.56	0.34	0.21	0.66	0.4	0.54	0.26	0.58	0.79	0.41

KY523104.1_AUL77835.1_MF405918.2_QKU33775.1_PUTATIVE_ORFAN_CDS	0.32	0.42	0.34	0.13	0	0.26	0.55	0.25	0.38	0.25	0.39
KY523104.1_AUL77836.1_MF405918.2_QKU33776.1_SERINE/THREONINE_PROTEIN_KINASE_CDS	0.38	0.18	0.28	0.22	0.49	0.27	0.54	0.26	0.44	0.35	0.4
KY523104.1_AUL77837.1_MF405918.2_QKU33777.1_HYPOTHETICAL_PROTEIN_CDS	0.43	0.51	0.49	0	0.04	0.12	0.37	0.71	0.3	0.04	0.43
KY523104.1_AUL77838.1_MF405918.2_QKU33778.1_KINESIN-LIKE_PROTEIN_CDS	0.41	0.61	0.07	0	0.05	0.64	0.86	0.17	0.77	0	0.61
KY523104.1_AUL77839.1_MF405918.2_QKU33779.1_PUTATIVE_ORFAN_CDS	0.18	0.13	0.19	0.02	0.2	0.51	0.18	0.04	0.09	0.31	0.06
KY523104.1_AUL77840.1_MF405918.2_QKU33780.1_PUTATIVE_ENDONUCLEASE_4_CDS	0.39	0.65	0.46	0.73	0	0.39	0.23	0.34	0.47	0.32	0.26
KY523104.1_AUL77841.1_MF405918.2_QKU33781.1_PUTATIVE_LOW_COMPLEXITY_PROTEIN_CDS	0.39	0.4	0.34	0.03	0.45	0.25	0.17	0.64	0.54	0.54	0.27
KY523104.1_AUL77842.1_MF405918.2_QKU33782.1_RIBONUCLEASE_H_CDS	0.39	0.69	0.44	0	0.01	0.02	0.02	0.7	0.36	0.02	0.33
KY523104.1_AUL77843.1_MF405918.2_QKU33783.1_PUTATIVE_ORFAN_CDS	0.36	0.01	0.19	0	0	0	0	0.8	0.34	0.02	0.22
KY523104.1_AUL77844.1_MF405918.2_QKU33784.1_POLY(ADP-RIBOSE)_POLYMERASE_CDS	0.47	0.71	0.38	0.33	0.19	0.43	0.44	0.31	0.42	0.1	0.48
KY523104.1_AUL77845.1_MF405918.2_QKU33785.1_PUTATIVE_ENDO/EXCINUCLEASE_AMINO_TERMINAL_DOMAINPR(	0.24	0.21	0.15	0.04	0.01	0.2	0.17	0.02	0.12	0.41	0.29
KY523104.1_AUL77846.1_MF405918.2_QKU33786.1_PUTATIVE_SERINE_THREONINE_PROTEIN_KINASE_CDS	0.67	0.08	0.39	0.12	0.21	0.43	0.28	0.6	0.23	0.18	0.45
KY523104.1_AUL77847.1_MF405918.2_QKU33787.1_PUTATIVE_ORFAN_CDS	0.22	0.27	0.17	0.07	0.01	0.12	0.19	0.03	0.33	0.43	0.19
KY523104.1_AUL77848.1_MF405918.2_QKU33788.1_PROLYL-TRNA_SYNTHETASE_CDS	0.47	0.23	0.2	0.18	0.23	0.2	0.58	0.06	0.41	0.36	0.51
KY523104.1_AUL77849.1_MF405918.2_QKU33789.1_HYPOTHETICAL_PROTEIN_CDS	0.04	0.05	0.1	0	0.06	0.09	0.07	0.01	0.01	0.28	0.05
KY523104.1_AUL77850.1_MF405918.2_QKU33790.1_HYPOTHETICAL_PROTEIN_CDS	0.4	0.62	0.37	0.09	0.14	0.51	0.5	0.54	0.39	0.23	0.37
KY523104.1_AUL77851.1_MF405918.2_QKU33791.1_NAD-DEPENDENT_DNA_LIGASE_CDS	0.36	0.29	0.71	0.25	0.18	0.37	0.44	0.32	0.52	0.44	0.34
KY523104.1_AUL77852.1_MF405918.2_QKU33792.1_COLLAGEN-LIKE_PROTEIN_1_CDS	0.41	0.31	0.26	0.37	0.5	0.6	0.52	0.28	0.44	0.58	0.46
KY523104.1_AUL77853.1_MF405918.2_QKU33793.1_HYPOTHETICAL_PROTEIN_CDS	0.4	0.63	0.3	0.11	0.13	0.72	0.66	0.2	0.55	0.04	0.42
KY523104.1_AUL77854.1_MF405918.2_QKU33794.1_HYPOTHETICAL_PROTEIN_CDS	0.32	0.03	0.42	0.65	0.52	0.52	0.21	0.39	0.4	0.49	0.3
KY523104.1_AUL77855.1_MF405918.2_QKU33795.1_HYPOTHETICAL_PROTEIN_CDS	0.37	0.41	0.38	0.53	0.29	0.38	0.33	0.25	0.42	0.4	0.44
KY523104.1_AUL77856.1_MF405918.2_QKU33796.1_HYPOTHETICAL_PROTEIN_CDS	0.74	0.11	0.15	0.2	0.04	0.2	0.54	0.02	0.4	0.57	0.52
KY523104.1_AUL77857.1_MF405918.2_QKU33797.1_PUTATIVE_ORFAN_CDS	0.22	0.05	0.34	0	0.53	0.29	0.16	0.29	0.06	0.38	0.08
KY523104.1_AUL77858.1_MF405918.2_QKU33798.1_RING_FINGER_PROTEIN_146_CDS	0.76	0.02	0.28	0.33	0.31	0.31	0.12	0.15	0.46	0.38	0.49
KY523104.1_AUL77859.1_MF405918.2_QKU33799.1_PUTATIVE_PROTEIN_PHOSPHATASE_2C_CDS	0.4	0.15	0.27	0.27	0.46	0.35	0.48	0.3	0.53	0.45	0.44
KY523104.1_AUL77860.1_MF405918.2_QKU33800.1_MG600_PROTEIN_CDS	0.28	0.47	0.37	0.13	0.4	0.56	0.14	0.19	0.45	0.55	0.17
KY523104.1_AUL77861.1_MF405918.2_QKU33801.1_MG386_PROTEIN_CDS	0.28	0.22	0.26	0.15	0.15	0.26	0.25	0.31	0.08	0.4	0.41
KY523104.1_AUL77862.1_MF405918.2_QKU33802.1_PUTATIVE_ORFAN_CDS	0.36	0.05	0.37	0.18	0.49	0.43	0.09	0.5	0.48	0.38	0.4
KY523104.1_AUL77863.1_MF405918.2_QKU33803.1_HYPOTHETICAL_PROTEIN_CDS	0.87	0.72	0.48	0.29	0.33	0.44	0.15	0.05	0.45	0.53	0.55
KY523104.1_AUL77864.1_MF405918.2_QKU33804.1_HYPOTHETICAL_PROTEIN_CDS	0.19	0.28	0.17	0.19	0	0.09	0.17	0.01	0.06	0.36	0.2
KY523104.1_AUL77865.1_MF405918.2_QKU33805.1_HYPOTHETICAL_PROTEIN_CDS	0.19	0.12	0.12	0.01	0	0.09	0.17	0.02	0.65	0.38	0.15
KY523104.1_AUL77866.1_MF405918.2_QKU33806.1_PUTATIVE_ORFAN_CDS	0.17	0.14	0.09	0	0.01	0.04	0.36	0.02	0.05	0.28	0.06
KY523104.1_AUL77867.1_MF405918.2_QKU33807.1_MG596_PROTEIN_CDS	0.17	0.55	0.38	0.3	0.01	0.24	0.2	0.07	0.2	0.31	0.11
KY523104.1_AUL77868.1_MF405918.2_QKU33808.1_MG595_PROTEIN_CDS	0.83	0.41	0.34	0.3	0.43	0.25	0.39	0.16	0.49	0.5	0.66
KY523104.1_AUL77869.1_MF405918.2_QKU33809.1_PUTATIVE_ORFAN_CDS	0.27	0.23	0.34	0.16	0.35	0.52	0.3	0.18	0.46	0.51	0.3
KY523104.1_AUL77870.1_MF405918.2_QKU33810.1_RIBONUCLEOTIDE_REDUCTASE_SMALL_SUBUNIT_CDS	0.44	0.4	0.4	0.16	0.23	0.48	0.28	0.46	0.26	0.31	0.43
KY523104.1_AUL77871.1_MF405918.2_QKU33811.1_PUTATIVE_ORFAN_CDS	0.32	0.2	0.38	0.23	0.49	0.34	0.39	0.37	0.29	0.48	0.41
KY523104.1_AUL77872.1_MF405918.2_QKU33812.1_PUTATIVE_RIBONUCLEOSIDE_DI_PHOSPHATE_REDUCTASE_ALPHA	0.41	0.72	0.28	0.45	0.01	0.76	0.46	0.21	0.55	0.4	0.35
KY523104.1_AUL77873.1_MF405918.2_QKU33813.1_PUTATIVE_ORFAN_CDS	0.38	0.31	0.34	0.26	0.53	0.37	0.38	0.34	0.37	0.52	0.43
KY523104.1_AUL77874.1_MF405918.2_QKU33814.1_PUTATIVE_ORFAN_CDS	0.19	0.46	0.31	0.11	0	0.24	0.06	0.04	0.35	0.39	0.22
KY523104.1_AUL77875.1_MF405918.2_QKU33815.1_MG215_PROTEIN_CDS	0.29	0.45	0.37	0.05	0	0.6	0.24	0.23	0.36	0.29	0.28
KY523104.1_AUL77876.1_MF405918.2_QKU33816.1_PUTATIVE_ANKYRIN_REPEAT_PROTEIN_CDS	0.74	0.28	0.47	0.29	0.12	0.5	0.39	0.64	0.26	0.24	0.47

KY523104.1_AUL77877.1_MF405918.2_QKU33817.1_FORMAMIDOPYRIMIDINE-DNA_GLYCOSYLASE_CDS	0.24	0.22	0.31	0.08	0.03	0.45	0.42	0.34	0.07	0.43	0.24
KY523104.1_AUL77878.1_MF405918.2_QKU33818.1_ATP_BINDING_CASSETTE_SUB_FAMILY_F_CDS	0.42	0.55	0.05	0	0.25	0.16	0.71	0.75	0.28	0	0.45
KY523104.1_AUL77879.1_MF405918.2_QKU33819.1_SACCHAROPINE_DEHYDROGENASE_CDS	0.45	0.57	0.96	0.35	0.63	0.45	0.1	0.48	0.28	0.33	0.4
KY523104.1_AUL77880.1_MF405918.2_QKU33820.1_6-PHOSPHATE_N-ACETYLTRANSFERASE_CDS	0.27	0.11	0.2	0.03	0.03	0.18	0.39	0.06	0.06	0.37	0.31
KY523104.1_AUL77881.1_MF405918.2_QKU33821.1_PUTATIVE_ORFAN_CDS	0.54	0.02	0.16	0	0	0.1	0.01	0.02	0.02	0.29	0.53
KY523104.1_AUL77882.1_MF405918.2_QKU33822.1_ANKYRIN_REPEAT_PROTEIN_CDS	0.39	0.51	0.38	0.3	0.34	0.5	0.32	0.51	0.45	0.35	0.4
KY523104.1_AUL77883.1_MF405918.2_QKU33823.1_COLLAGEN-LIKE_PROTEIN_CDS	0.53	0.3	0.25	0.26	0.33	0.37	0.46	0.12	0.44	0.48	0.48
KY523104.1_AUL77884.1_MF405918.2_QKU33824.1_HYPOTHETICAL_PROTEIN_CDS	0.26	0.55	0.39	0.09	0.01	0.37	0.2	0.52	0.35	0.23	0.32
KY523104.1_AUL77885.1_MF405918.2_QKU33825.1_HYPOTHETICAL_PROTEIN_CDS	0.77	0.17	0.11	0.28	0.01	0.27	0.18	0.01	0.48	0.2	0.87
KY523104.1_AUL77886.1_MF405918.2_QKU33826.1_DNA_POLYMERASE_FAMILY_X_CDS	0.4	0.35	0.37	0.07	0.04	0.3	0.58	0.85	0.61	0.39	0.36
KY523104.1_AUL77887.1_MF405918.2_QKU33827.1_PUTATIVE_UBIQUITIN-SPECIFIC_PROTEASE_CDS	0.4	0.23	0.29	0.23	0.47	0.29	0.56	0.24	0.41	0.43	0.46
KY523104.1_AUL77888.1_MF405918.2_QKU33828.1_PUTATIVE_RNA_METHYLASE_CDS	0.21	0.27	0.22	0.05	0.01	0.21	0.47	0.21	0.04	0.36	0.23
KY523104.1_AUL77889.1_MF405918.2_QKU33829.1_SERINE_TRNA-SYNTHETASE_CDS	0.4	0.1	0.25	0.07	0.44	0.24	0.61	0.11	0.39	0.09	0.47
KY523104.1_AUL77890.1_MF405918.2_QKU33830.1_MG583_PROTEIN_CDS	0.31	0.16	0.28	0.43	0.01	0.39	0.16	0.03	0.27	0.45	0.42
KY523104.1_AUL77891.1_MF405918.2_QKU33831.1_HYPOTHETICAL_PROTEIN_CDS	0.49	0.25	0.32	0.37	0.32	0.32	0.31	0.2	0.42	0.44	0.46
KY523104.1_AUL77892.1_MF405918.2_QKU33832.1_PUTATIVE_ORFAN_CDS	0.23	0.04	0.28	0.02	0.06	0.16	0.19	0.12	0.53	0.37	0.16
KY523104.1_AUL77893.1_MF405918.2_QKU33833.1_B-FAMILY_DNA_POLYMERASE_CDS	0.48	0.61	0.13	0.13	0.04	0.7	0.59	0.27	0.55	0.24	0.52
KY523104.1_AUL77894.1_MF405918.2_QKU33834.1_PUTATIVE_ORFAN_CDS	0.11	0.13	0.09	0	0.01	0.05	0.31	0.02	0.06	0.24	0.14
KY523104.1_AUL77895.1_MF405918.2_QKU33835.1_HYPOTHETICAL_PROTEIN_CDS	0.28	0.28	0.37	0.48	0.13	0.49	0.45	0.28	0.48	0.46	0.31
KY523104.1_AUL77896.1_MF405918.2_QKU33836.1_HYPOTHETICAL_PROTEIN_CDS	0.52	0.44	0.39	0.25	0.45	0.35	0.35	0.4	0.28	0.36	0.45
KY523104.1_AUL77897.1_MF405918.2_QKU33837.1_PUTATIVE_METALLOPEPTIDASE_WLM_CDS	0.27	0.14	0.23	0.05	0.51	0.23	0.18	0.46	0.04	0.39	0.29
KY523104.1_AUL77898.1_MF405918.2_QKU33838.1_PUTATIVE_ORFAN_CDS	0.39	0.02	0.34	0.05	0	0.65	0.58	0.4	0.37	0.31	0.36
KY523104.1_AUL77899.1_MF405918.2_QKU33839.1_PUTATIVE_EARLY_TRANSCRIPTION_FACTOR_CDS	0.4	0.81	0.38	0.51	0.58	0.39	0.63	0.8	0.52	0	0.2
KY523104.1_AUL77900.1_MF405918.2_QKU33840.1_PUTATIVE_ORFAN_CDS	0.22	0.19	0.13	0.01	0.01	0.06	0.2	0.01	0.03	0.32	0.34
KY523104.1_AUL77901.1_MF405918.2_QKU33841.1_ABC_TRANSPORTER_CDS	0.61	0.18	0.1	0.05	0.34	0.07	0.64	0.03	0.46	0.37	0.62
KY523104.1_AUL77902.1_MF405918.2_QKU33842.1_HYPOTHETICAL_PROTEIN_CDS	0.2	0.36	0.41	0.13	0.08	0.26	0.2	0.04	0.12	0.43	0.17
KY523104.1_AUL77903.1_MF405918.2_QKU33843.1_HYPOTHETICAL_PROTEIN_CDS	0.4	0.51	0.42	0.98	0.27	0.4	0.29	0.21	0.42	0.18	0.46
KY523104.1_AUL77904.1_MF405918.2_QKU33844.1_N_TRANS_DOMAIN_CONTAINING_PROTEIN_CDS	0.31	0.06	0.23	0.1	0.04	0.19	0.16	0.33	0.11	0.36	0.43
KY523104.1_AUL77905.1_MF405918.2_QKU33845.1_MG574_PROTEIN_CDS	0.26	0.33	0.27	0.31	0.01	0.24	0.11	0.04	0.49	0.47	0.25
KY523104.1_AUL77906.1_MF405918.2_QKU33846.1_PUTATIVE_E3_UBIQUITIN-PROTEIN_LIGASE_AT4G11680-LIKE_CDS	0.88	0.04	0.24	0.31	0.29	0.27	0.17	0.07	0.48	0.32	0.71
KY523104.1_AUL77907.1_MF405918.2_QKU33847.1_HYPOTHETICAL_PROTEIN_CDS	0.84	0.18	0.26	0.35	0	0.33	0.1	0.08	0.46	0.34	0.61
KY523104.1_AUL77908.1_MF405918.2_QKU33848.1_PUTATIVE_ORFAN_CDS	0.14	0.25	0.13	0.01	0	0.14	0.34	0.01	0.02	0.36	0.11
KY523104.1_AUL77909.1_MF405918.2_QKU33849.1_PUTATIVE_ORFAN_CDS	0.9	0.17	0.3	0.04	0.04	0.18	0.59	0.19	0.17	0.45	0.25
KY523104.1_AUL77909.1_MF405918.2_QKU33849.1_PUTATIVE_ORFAN_CDS	0.1	0.05	0.05	0	0	0.02	0.23	0	0.01	0.16	0.09
KY523104.1_AUL77910.1_MF405918.2_QKU33850.1_PUTATIVE_ORFAN_CDS	0.07	0.02	0.04	0	0	0.01	0.13	0	0	0.13	0.04
KY523104.1_AUL77910.1_MF405918.2_QKU33850.1_PUTATIVE_ORFAN_CDS	0.09	0.07	0.07	0	0.01	0.04	0.36	0.02	0.05	0.21	0.14
KY523104.1_AUL77909.1_MF405918.2_QKU33849.1_PUTATIVE_ORFAN_CDS	0.08	0.04	0.06	0	0	0.05	0.5	0.03	0.1	0.14	0.05
KY523104.1_AUL77911.1_MF405918.2_QKU33851.1_RNA-BINDING_PROTEIN_CABEZA_CDS	0.89	0.05	0.25	0.29	0	0.42	0.18	0.07	0.46	0.25	0.75
KY523104.1_AUL77912.1_MF405918.2_QKU33852.1_PUTATIVE_ORFAN_CDS	0.17	0.11	0.33	0.01	0.01	0.21	0.46	0.52	0.19	0.39	0.11
KY523104.1_AUL77913.1_MF405918.2_QKU33853.1_HYPOTHETICAL_PROTEIN_CDS	0.35	0.12	0.29	0.23	0.01	0.39	0.4	0.06	0.37	0.42	0.45
KY523104.1_AUL77914.1_MF405918.2_QKU33854.1_HYPOTHETICAL_PROTEIN_CDS	0.69	0.43	0.32	0.22	0.63	0.38	0.39	0.24	0.34	0.54	0.48
KY523104.1_AUL77915.1_MF405918.2_QKU33855.1_HYPOTHETICAL_PROTEIN_CDS	0.27	0.47	0.32	0.13	0	0.22	0.48	0.19	0.5	0.37	0.24

KY523104.1_AUL77916.1_MF405918.2_QKU33856.1_PUTATIVE_ORFAN_CDS	0.22	0.05	0.13	0.01	0.25	0.11	0.27	0.04	0.23	0.32	0.26
KY523104.1_AUL77917.1_MF405918.2_QKU33857.1_HYPOTHETICAL_PROTEIN_CDS	0.24	0.13	0.22	0.04	0.03	0.17	0.4	0.06	0.21	0.36	0.18
KY523104.1_AUL77918.1_MF405918.2_QKU33858.1_HYPOTHETICAL_PROTEIN_CDS	0.4	0.57	0.4	0.19	0.54	0.39	0.53	0.27	0.39	0.33	0.45
KY523104.1_AUL77918.1_MF405918.2_QKU33858.1_HYPOTHETICAL_PROTEIN_CDS	0.17	0.15	0.21	0.02	0.43	0.41	0.04	0.03	0.31	0.43	0.06
KY523104.1_AUL77919.1_MF405918.2_QKU33859.1_PUTATIVE_ORFAN_CDS	0.17	0.15	0.21	0.02	0.43	0.41	0.04	0.03	0.31	0.43	0.06
KY523104.1_AUL77920.1_MF405918.2_QKU33860.1_HYPOTHETICAL_PROTEIN_CDS	0.56	0.34	0.22	0.53	0.15	0.65	0.19	0.2	0.5	0.43	0.46
KY523104.1_AUL77921.1_MF405918.2_QKU33861.1_HYPOTHETICAL_PROTEIN_CDS	0.42	0.07	0.47	0.18	0.26	0.35	0.57	0.11	0.29	0.29	0.46
KY523104.1_AUL77922.1_MF405918.2_QKU33862.1_TRANSCRIPTION_FACTOR_S-II-RELATED_PROTEIN_CDS	0.78	0.66	0.38	0.27	0.08	0.39	0.35	0.04	0.43	0.45	0.52
KY523104.1_AUL77923.1_MF405918.2_QKU33863.1_HYPOTHETICAL_PROTEIN_CDS	0.3	0.54	0.38	0.19	0.01	0.33	0.19	0.28	0.44	0.34	0.36
KY523104.1_AUL77924.1_MF405918.2_QKU33864.1_HYPOTHETICAL_PROTEIN_CDS	0.31	0.35	0.37	0.51	0.05	0.39	0.33	0.25	0.26	0.4	0.41
KY523104.1_AUL77925.1_MF405918.2_QKU33865.1_HYPOTHETICAL_PROTEIN_CDS	0.59	0.29	0.32	0.18	0.25	0.32	0.48	0.44	0.18	0.4	0.45
KY523104.1_AUL77926.1_MF405918.2_QKU33866.1_HYPOTHETICAL_PROTEIN_CDS	0.58	0.09	0.28	0.3	0.33	0.28	0.15	0.1	0.43	0.42	0.46
KY523104.1_AUL77927.1_MF405918.2_AUL79214.2_HYPOTHETICAL_PROTEIN_CDS	0.52	0.58	0.33	0.34	0.09	0.5	0.27	0.05	0.47	0.38	0.48
KY523104.1_AUL77928.1_MF405918.2_QKU33867.1_MG18_PROTEIN_CDS	0.39	0.59	0.35	0.1	0.07	0.37	0.5	0.27	0.44	0.27	0.41
KY523104.1_AUL77929.1_MF405918.2_QKU33868.1_POLYA_POLYMERASE_CATALITIC_SUBUNIT_CDS	0.77	0.63	0.26	0	0.1	0.17	0.24	0.94	0.35	0	0.49
KY523104.1_AUL77930.1_MF405918.2_QKU33869.1_HYPOTHETICAL_PROTEIN_CDS	0.42	0.49	0.29	0.13	0.46	0.14	0.6	0.92	0.71	0.53	0.38
KY523104.1_AUL77931.1_MF405918.2_QKU33870.1_HYPOTHETICAL_PROTEIN_CDS	0.26	0.16	0.25	0	0	0.32	0.58	0.14	0.37	0.24	0.12
KY523104.1_AUL77932.1_MF405918.2_QKU33871.1_PUTATIVE_RIBONUCLEASE_3_CDS	0.4	0.55	0.32	0.18	0.32	0.31	0.54	0.41	0.51	0.23	0.41
KY523104.1_AUL77933.1_MF405918.2_QKU33872.1_HYPOTHETICAL_PROTEIN_CDS	0.63	0.33	0.2	0.37	0.7	0.19	0.5	0.1	0.49	0.53	0.59
KY523104.1_AUL77934.1_MF405918.2_QKU33873.1_PUTATIVE_ORFAN_CDS	0.25	0.1	0.13	0.02	0.01	0.08	0.11	0.03	0.57	0.36	0.32
KY523104.1_AUL77935.1_MF405918.2_QKU33874.1_HYPOTHETICAL_PROTEIN_CDS	0.15	0.47	0.22	0.06	0	0.17	0.27	0.02	0.07	0.37	0.11
KY523104.1_AUL77936.1_MF405918.2_QKU33875.1_HYPOTHETICAL_PROTEIN_CDS	0.41	0.01	0.33	0	0.09	0.11	0.38	0.73	0.31	0.14	0.44
KY523104.1_AUL77937.1_MF405918.2_QKU33876.1_HYPOTHETICAL_PROTEIN_CDS	0.4	0.37	0.26	0.28	0.19	0.25	0.62	0.21	0.38	0.03	0.46
KY523104.1_AUL77938.1_MF405918.2_QKU33877.1_HYPOTHETICAL_PROTEIN_CDS	0.41	0.64	0.36	0.22	0.48	0.46	0.56	0.38	0.54	0.21	0.44
KY523104.1_AUL77939.1_MF405918.2_QKU33878.1_HYPOTHETICAL_PROTEIN_CDS	0.14	0.02	0.14	0.01	0	0.49	0.24	0.02	0.14	0.3	0.07
KY523104.1_AUL77940.1_MF405918.2_QKU33879.1_HYPOTHETICAL_PROTEIN_CDS	0.12	0.02	0.05	0	0	0.04	0.11	0	0	0.17	0.03
KY523104.1_AUL77941.1_MF405918.2_QKU33880.1_SNF2_FAMILY_HELICASE_CDS	0.4	0.21	0.27	0.26	0.11	0.37	0.57	0.41	0.49	0.33	0.47
KY523104.1_AUL77942.1_MF405918.2_AUL79230.2_PUTATIVE_ORFAN_CDS	0.17	0.11	0.09	0	0	0.04	0.19	0.03	0.05	0.24	0.2
KY523104.1_AUL77943.1_MF405918.2_QKU33881.1_PUTATIVE_ORFAN_CDS	0.43	0	0.37	0	1	0.44	0	0.17	0.13	0.61	0.27
KY523104.1_AUL77944.1_MF405918.2_QKU33882.1_HYPOTHETICAL_PROTEIN_CDS	0.4	0.72	0.39	0.5	0	0.32	0.17	0.14	0.5	0.28	0.29
KY523104.1_AUL77945.1_MF405918.2_QKU33883.1_HYPOTHETICAL_PROTEIN_CDS	0.25	0.34	0.27	0.16	0.37	0.25	0.38	0.09	0.13	0.39	0.35
KY523104.1_AUL77946.1_MF405918.2_QKU33884.1_ASPARTYL-TRNA_SYNTHETASE_CDS	0.37	0.19	0.37	0.1	0.05	0.33	0.33	0.53	0.52	0.47	0.4
KY523104.1_AUL77947.1_MF405918.2_QKU33885.1_YQAJ_VIRAL_RECOMBINASE_FAMILY_PROTEIN_CDS	0.36	0.36	0.31	0.21	0.22	0.48	0.51	0.23	0.51	0.38	0.35
KY523104.1_AUL77948.1_MF405918.2_QKU33886.1_HOMOSPERMIDINE_SYNTHASE_CDS	0.35	0.41	0.46	0.21	0.37	0.45	0.3	0.48	0.39	0.4	0.4
KY523104.1_AUL77949.1_MF405918.2_QKU33887.1_THIOL_PROTEASE_CDS	0.22	0.5	0.29	0.09	0	0.32	0.46	0.22	0.26	0.36	0.22
KY523104.1_AUL77950.1_MF405918.2_QKU33888.1_PUTATIVE_ORFAN_CDS	0.4	0.89	0.2	0.12	0.14	0.04	0.03	0.01	0.39	0	0.03
KY523104.1_AUL77951.1_MF405918.2_QKU33889.1_PUTATIVE_LOW_COMPLEXITY_PROTEIN_CDS	0.4	0.67	0.44	0.25	0.01	0.34	0.44	0.32	0.46	0.14	0.3
KY523104.1_AUL77952.1_MF405918.2_QKU33890.1_HYPOTHETICAL_PROTEIN_CDS	0.4	0.68	0.4	0.35	0.14	0.31	0.37	0.46	0.45	0.12	0.31
KY523104.1_AUL77953.1_MF405918.2_QKU33891.1_PUTATIVE_ORFAN_CDS	0.22	0.3	0.35	0.07	0.34	0.26	0.42	0.5	0.12	0.38	0.23
KY523104.1_AUL77954.1_MF405918.2_QKU33892.1_PUTATIVE_ORFAN_CDS	0.31	0.37	0.42	0.02	0	0.23	0.19	0.53	0.53	0.48	0.13
KY523104.1_AUL77955.1_MF405918.2_QKU33893.1_PUTATIVE_ORFAN_CDS	0.31	0.37	0.33	0.16	0.46	0.32	0.14	0.23	0.27	0.4	0.41
KY523104.1_AUL77956.1_MF405918.2_QKU33894.1_PUTATIVE_ORFAN_CDS	0.9	0.17	0.3	0.04	0.04	0.18	0.59	0.19	0.17	0.45	0.25

KY523104.1_AUL77956.1_MF405918.2_QKU33894.1_PUTATIVE_ORFAN_CDS	0.38	0.61	0.34	0.05	0.01	0.33	0.43	0.58	0.43	0.18	0.33
KY523104.1_AUL77955.1_MF405918.2_QKU33893.1_PUTATIVE_ORFAN_CDS	0.9	0.17	0.3	0.04	0.04	0.18	0.59	0.19	0.17	0.45	0.25
KY523104.1_AUL77957.1_MF405918.2_QKU33895.1_RNA_POLYMERASE_SUBUNIT_N_CDS	0.79	0.31	0.33	0.31	0.34	0.29	0.07	0.1	0.3	0.43	0.5
KY523104.1_AUL77958.1_MF405918.2_QKU33896.1_A32-LIKE_PACKAGING_ATPASE_CDS	0.42	0.8	0.38	0.63	0.03	0.22	0.05	0.12	0.5	0.1	0.18
KY523104.1_AUL77959.1_MF405918.2_QKU33897.1_PUTATIVE_ORFAN_CDS	0.21	0.11	0.22	0.01	0.02	0.19	0.43	0.05	0.07	0.37	0.14
KY523104.1_AUL77960.1_MF405918.2_QKU33898.1_PUTATIVE_DNA_MISMATCH_REPAIR_PROTEIN_MUTS-LIKE_PROTE	0.45	0.45	0.15	0.1	0.74	0.68	0.62	0.21	0.34	0.88	0.48
KY523104.1_AUL77961.1_MF405918.2_QKU33899.1_HYPOTHETICAL_PROTEIN_CDS	0.41	0.71	0.4	0.35	0	0.23	0.16	0.12	0.45	0.15	0.31
KY523104.1_AUL77962.1_MF405918.2_QKU33900.1_HYPOTHETICAL_PROTEIN_CDS	0.01	0	0.06	0	0	0.05	0	0	0.74	0.31	0.01
KY523104.1_AUL77962.1_MF405918.2_QKU33900.1_HYPOTHETICAL_PROTEIN_CDS	0.27	0.33	0.28	0.44	0.37	0.3	0.2	0.08	0.12	0.43	0.33
KY523104.1_AUL77963.1_MF405918.2_QKU33901.1_THIOREDOXIN_DOMAIN-CONTAINING_PROTEIN_CDS	0.31	0.09	0.29	0.2	0.29	0.38	0.31	0.08	0.47	0.47	0.43
KY523104.1_AUL77964.1_MF405918.2_QKU33902.1_PUTATIVE_GLYCOSYLTRANSFERASE_CDS	0.67	0.36	0.65	0.74	0.58	0.76	0.44	0.6	0.48	0.24	0.55
KY523104.1_AUL77965.1_MF405918.2_QKU33903.1_HYPOTHETICAL_PROTEIN_CDS	0.42	0.25	0.33	0.3	0.13	0.35	0.19	0.12	0.46	0.41	0.46
KY523104.1_AUL77964.1_MF405918.2_QKU33902.1_PUTATIVE_GLYCOSYLTRANSFERASE_CDS	0.41	0.04	0.05	0	0	0.06	0.06	0.01	0.01	0.19	0.46
KY523104.1_AUL77966.1_MF405918.2_QKU33904.1_HYPOTHETICAL_PROTEIN_CDS	0.57	0.22	0.27	0.21	0.01	0.35	0.08	0.06	0.49	0.4	0.46
KY523104.1_AUL77967.1_MF405918.2_QKU33905.1_PUTATIVE_BIFUNCTIONAL_SAT/APS_KINASE_2_CDS	0.4	0.5	0.16	0.03	0.1	0.13	0.64	0.28	0.37	0.01	0.39
KY523104.1_AUL77968.1_MF405918.2_QKU33906.1_HYPOTHETICAL_PROTEIN_CDS	0.41	0.7	0.34	0.47	0.28	0.51	0.35	0.22	0.53	0.39	0.45
KY523104.1_AUL77969.1_MF405918.2_QKU33907.1_PUTATIVE_ORFAN_CDS	0.2	0.15	0.12	0.04	0.01	0.1	0.19	0.01	0.32	0.37	0.17
KY523104.1_AUL77970.1_MF405918.2_QKU33908.1_PUTATIVE_NUCLEOTIDYL_TRANSFERASE_CDS	0.43	0.6	0.26	0.22	0.2	0.56	0.52	0.26	0.46	0.33	0.48
KY523104.1_AUL77971.1_MF405918.2_QKU33909.1_HYPOTHETICAL_PROTEIN_CDS	0.72	0.74	0.47	0.19	0.07	0.64	0.31	0.08	0.46	0.43	0.6
KY523104.1_AUL77972.1_MF405918.2_QKU33910.1_PUTATIVE_ORFAN_CDS	0.4	0.23	0.33	0.01	0.52	0.24	0.33	0.25	0.58	0.46	0.36
KY523104.1_AUL77973.1_MF405918.2_QKU33911.1_PUTATIVE_ORFAN_CDS	0.33	0.4	0.35	0.19	0.02	0.38	0.25	0.21	0.45	0.39	0.42
KY523104.1_AUL77974.1_MF405918.2_QKU33912.1_HYPOTHETICAL_PROTEIN_CDS	0.26	0.45	0.39	0.09	0.18	0.42	0.5	0.44	0.31	0.34	0.22
KY523104.1_AUL77975.1_MF405918.2_QKU33913.1_HYPOTHETICAL_PROTEIN_CDS	0.6	0.2	0.3	0.25	0.36	0.29	0.52	0.21	0.46	0.39	0.52
KY523104.1_AUL77976.1_MF405918.2_QKU33914.1_HYPOTHETICAL_PROTEIN_CDS	0.24	0.51	0.4	0.1	0.03	0.3	0.21	0.42	0.18	0.3	0.32
KY523104.1_AUL77977.1_MF405918.2_QKU33915.1_AMINOTRANSFERASE_CDS	0.4	0.29	0.35	0.27	0.42	0.36	0.42	0.49	0.47	0.44	0.45
KY523104.1_AUL77978.1_MF405918.2_QKU33916.1_HYPOTHETICAL_PROTEIN_CDS	0.4	0.31	0.33	0.15	0.38	0.64	0.54	0.52	0.42	0.46	0.43
KY523104.1_AUL77979.1_MF405918.2_QKU33917.1_MULTIPLE_GLYCOSYLTRANSFERASE_DOMAIN_CONTAINING_PROT	0.39	0.3	0.24	0.02	0.02	0.22	0.59	0.32	0.4	0.12	0.23
KY523104.1_AUL77980.1_MF405918.2_QKU33918.1_HYPOTHETICAL_PROTEIN_CDS	0.31	0.34	0.37	0.54	0.11	0.52	0.23	0.26	0.42	0.53	0.35
KY523104.1_AUL77981.1_MF405918.2_QKU33919.1_PUTATIVE_ORFAN_CDS	0.69	0.68	0.38	0.3	0.06	0.53	0.33	0.04	0.48	0.43	0.55
KY523104.1_AUL77982.1_MF405918.2_QKU33920.1_ACETYLTRANSFERASE_CDS	0.26	0.34	0.39	0.05	0.01	0.53	0.15	0.5	0.22	0.27	0.27
KY523104.1_AUL77983.1_MF405918.2_QKU33921.1_4_EPIMERASE_CDS	0.39	0.39	0.24	0.07	0.37	0.17	0.6	0.23	0.41	0.08	0.41
KY523104.1_AUL77984.1_MF405918.2_QKU33922.1_HYPOTHETICAL_PROTEIN_CDS	0.39	0.58	0.31	0.18	0.11	0.25	0.55	0.49	0.59	0.38	0.27
KY523104.1_AUL77985.1_MF405918.2_QKU33923.1_HYPOTHETICAL_PROTEIN_CDS	0.26	0.58	0.43	0.13	0.02	0.34	0.38	0.38	0.34	0.25	0.24
KY523104.1_AUL77986.1_MF405918.2_AUL79276.2_NAD-DEPENDENT_EPIMERASE/DEHYDRATASE_CDS	0.4	0.64	0.4	0.28	0.07	0.41	0.36	0.28	0.43	0.24	0.38
KY523104.1_AUL77987.1_MF405918.2_QKU33924.1_PUTATIVE_GLYCOSYLTRANSFERASE_CDS	0.25	0.58	0.4	0.11	0.04	0.35	0.41	0.34	0.42	0.29	0.23
KY523104.1_AUL77988.1_MF405918.2_QKU33925.1_PUTATIVE_UDP-GLUCOSE_6-DEHYDROGENASE_CDS	0.26	0.27	0.24	0.13	0.02	0.26	0.46	0.07	0.13	0.39	0.37
KY523104.1_AUL77989.1_MF405918.2_QKU33926.1_BIFUNCTIONAL_GLUTAMATE/PROLINE_TRNA-SYNTHETASE_CDS	0.44	0.52	0.38	0.37	0.28	0.4	0.33	0.13	0.43	0.4	0.46
KY523104.1_AUL77990.1_MF405918.2_QKU33927.1_PUTATIVE_ORFAN_CDS	0.09	0.14	0.1	0.01	0	0.08	0.09	0	0.07	0.34	0.07
KY523104.1_AUL77991.1_MF405918.2_QKU33928.1_HISTIDINE_TRNA_SYNTHETASE_CDS	0.42	0.72	0.31	0.19	0.13	0.5	0.56	0.62	0.49	0.02	0.42
KY523104.1_AUL77992.1_MF405918.2_QKU33929.1_HYPOTHETICAL_PROTEIN_CDS	0.17	0.52	0.3	0.1	0	0.23	0.35	0.08	0.26	0.35	0.13
KY523104.1_AUL77993.1_MF405918.2_AUL79282.2_PUTATIVE_ORFAN_CDS	0.51	0.13	0.34	0.22	0.49	0.33	0.37	0.36	0.15	0.44	0.44
KY523104.1_AUL77994.1_MF405918.2_QKU33930.1_PUTATIVE_ORFAN_CDS	0.39	0.54	0.34	0.09	0.2	0.55	0.53	0.32	0.44	0.27	0.42

KY523104.1_AUL77995.1_MF405918.2_QKU33931.1_MG533_PROTEIN_CDS	0.4	0.16	0.33	0.26	0.4	0.31	0.54	0.34	0.4	0.38	0.46
KY523104.1_AUL77996.1_MF405918.2_QKU33932.1_ACETYLPOLYAMINE_AMINOHYDROLASE_CDS	0.47	0.48	0.09	0.04	0	1	0.24	0.07	0.6	0.11	0.53
KY523104.1_AUL77997.1_MF405918.2_QKU33933.1_SYNAPTOSOMAL-ASSOCIATED_PROTEIN_CDS	0.21	0.49	0.33	0.11	0.01	0.26	0.32	0.1	0.21	0.34	0.21
KY523104.1_AUL77998.1_MF405918.2_QKU33934.1_PUTATIVE_ORFAN_CDS	0.22	0.24	0.15	0.06	0.02	0.1	0.24	0.02	0.08	0.37	0.28
KY523104.1_AUL77999.1_MF405918.2_QKU33935.1_PUTATIVE_ATP-DEPENDENT_RNA_HELICASE_CDS	0.41	0.7	0.33	0.05	0.36	0.53	0.62	0.38	0.52	0.01	0.49
KY523104.1_AUL78000.1_MF405918.2_QKU33936.1_HYPOTHETICAL_PROTEIN_CDS	0.4	0.64	0.42	0.23	0.55	0.4	0.33	0.51	0.44	0.25	0.36
KY523104.1_AUL78001.1_MF405918.2_QKU33937.1_PUTATIVE_FAD-LINKED_SULFHYDRYL_OXIDASE_CDS	0.19	0.13	0.23	0.02	0.01	0.06	0.28	0.03	0.09	0.3	0.14
KY523104.1_AUL78002.1_MF405918.2_QKU33938.1_HYPOTHETICAL_PROTEIN_CDS	0.44	0.28	0.34	0.3	0.36	0.43	0.29	0.22	0.45	0.44	0.45
KY523104.1_AUL78003.1_MF405918.2_QKU33939.1_HYPOTHETICAL_PROTEIN_CDS	0.28	0.08	0.32	0.03	0.01	0.24	0.13	0.22	0.5	0.4	0.27
KY523104.1_AUL78002.1_MF405918.2_QKU33938.1_HYPOTHETICAL_PROTEIN_CDS	0.09	0.06	0.04	0	0.1	0.04	0.18	0.04	0.02	0.28	0.36
KY523104.1_AUL78004.1_MF405918.2_QKU33940.1_PUTATIVE_ORFAN_CDS	0.4	0.59	0.41	0.49	0.21	0.53	0.3	0.46	0.51	0.51	0.36
KY523104.1_AUL78005.1_MF405918.2_QKU33941.1_PUTATIVE_ORFAN_CDS	0.29	0.4	0.38	0.2	0.38	0.39	0.3	0.33	0.46	0.44	0.28
KY523104.1_AUL78006.1_MF405918.2_QKU33942.1_PUTATIVE_ORFAN_CDS	0.58	0.27	0.25	0.17	0.05	0.59	0.2	0.11	0.5	0.35	0.46
KY523104.1_AUL78007.1_MF405918.2_QKU33943.1_ANKYRIN_REPEAT-CONTAINING_PROTEIN_CDS	0.67	0.38	0.36	0.86	0.3	0.39	0.46	0.58	0.45	0.63	0.56
KY523104.1_AUL78008.1_MF405918.2_QKU33944.1_PUTATIVE_ORFAN_CDS	0.25	0.2	0.25	0.05	0.51	0.3	0.19	0.07	0.05	0.42	0.27
KY523104.1_AUL78009.1_MF405918.2_QKU33945.1_HYPOTHETICAL_PROTEIN_CDS	0.4	0.16	0.36	0.26	0.58	0.41	0.31	0.39	0.32	0.56	0.44
KY523104.1_AUL78010.1_MF405918.2_QKU33946.1_HYPOTHETICAL_PROTEIN_CDS	0.38	0.01	0.25	0	0.01	0.16	0.3	0.57	0.06	0.28	0.4
KY523104.1_AUL78011.1_MF405918.2_QKU33947.1_GLYCOSYL_TRANSFERASE_CDS	0.46	0.1	0.35	0.21	0.44	0.36	0.36	0.18	0.16	0.42	0.43
KY523104.1_AUL78012.1_MF405918.2_QKU33948.1_HYPOTHETICAL_PROTEIN_CDS	0.29	0.15	0.28	0.12	0	0.54	0.3	0.13	0.23	0.41	0.39
KY523104.1_AUL78013.1_MF405918.2_QKU33949.1_GLYCOSYL_TRANSFERASE_CDS	0.47	0.02	0.25	0.23	0.43	0.39	0.17	0.15	0.47	0.33	0.47
KY523104.1_AUL78014.1_MF405918.2_QKU33950.1_HYPOTHETICAL_PROTEIN_CDS	0.2	0.44	0.36	0.19	0.24	0.33	0.18	0.05	0.3	0.4	0.16
KY523104.1_AUL78015.1_MF405918.2_QKU33951.1_NUDIX_HYDROLASE_CDS	0.48	0.08	0.18	0.13	0.18	0.18	0.59	0.07	0.48	0.33	0.52
KY523104.1_AUL78016.1_MF405918.2_QKU33952.1_DNA-DIRECTED_RNA_POLYMERASE_SUBUNIT_CDS	0.39	0.65	0.43	0.21	0.04	0.29	0.28	0.21	0.42	0.23	0.37
KY523104.1_AUL78017.1_MF405918.2_QKU33953.1_HYPOTHETICAL_PROTEIN_CDS	0.33	0.47	0.3	0.18	0.06	0.37	0.47	0.17	0.46	0.33	0.4
KY523104.1_AUL78018.1_MF405918.2_QKU33954.1_HYPOTHETICAL_PROTEIN_CDS	0.62	0.29	0.34	0.32	0.02	0.5	0.2	0.05	0.46	0.38	0.49
KY523104.1_AUL78019.1_MF405918.2_QKU33955.1_HYPOTHETICAL_PROTEIN_CDS	0.4	0.63	0.33	0.04	0.57	0.51	0.34	0.64	0.49	0.18	0.38
KY523104.1_AUL78020.1_MF405918.2_QKU33956.1_PUTATIVE_ORFAN_CDS	0.4	0.44	0.37	0.58	0.51	0.4	0.22	0.25	0.43	0.45	0.43
KY523104.1_AUL78021.1_MF405918.2_QKU33957.1_NTPASE_CDS	0.41	0.49	0.14	0.02	0.94	0.47	0.69	0.14	0.56	1	0.37
KY523104.1_AUL78022.1_MF405918.2_QKU33958.1_HYPOTHETICAL_PROTEIN_CDS	0.18	0.55	0.38	0.12	0	0.24	0.25	0.07	0.39	0.37	0.13
KY523104.1_AUL78023.1_MF405918.2_QKU33959.1_PUTATIVE_ORFAN_CDS	0.65	0.35	0.38	0.28	0.23	0.32	0.27	0.05	0.33	0.47	0.47
KY523104.1_AUL78024.1_MF405918.2_QKU33960.1_MG516_PROTEIN_CDS	0.24	0.27	0.33	0.12	0.46	0.4	0.32	0.15	0.11	0.43	0.19
KY523104.1_AUL78025.1_MF405918.2_QKU33961.1_HYPOTHETICAL_PROTEIN_CDS	0.27	0.51	0.41	0.15	0.01	0.31	0.24	0.12	0.28	0.36	0.33
KY523104.1_AUL78026.1_MF405918.2_QKU33962.1_PUTATIVE_ALPHA-KETOGLUTARATE-DEPENDENT_DIOXYGENASE_A	0.26	0.33	0.3	0.14	0.33	0.43	0.24	0.09	0.22	0.43	0.35
KY523104.1_AUL78027.1_MF405918.2_QKU33963.1_PUTATIVE_ORFAN_CDS	0.18	0.29	0.16	0.04	0	0.13	0.13	0.01	0.09	0.41	0.2
KY523104.1_AUL78028.1_MF405918.2_QKU33964.1_PUTATIVE_LEUCINE-RICH_REPEAT_PROTEIN_CDS	0.27	0.45	0.36	0.15	0.13	0.31	0.36	0.32	0.46	0.4	0.28
KY523104.1_AUL78029.1_MF405918.2_QKU33965.1_PUTATIVE_ORFAN_CDS	0.12	0.07	0.09	0	0.01	0.04	0.31	0.02	0.44	0.24	0.1
KY523104.1_AUL78030.1_MF405918.2_QKU33966.1_PUTATIVE_PHOSPHOLIPASE_D/NUCLEASE_CDS	0.25	0.19	0.16	0.07	0.1	0.33	0.14	0.02	0.14	0.43	0.23
KY523104.1_AUL78031.1_MF405918.2_QKU33967.1_MRNA_CAPPING_ENZYME_CDS	0.41	0.8	0.24	0.65	0.05	0.75	0.61	0.53	0.48	0	0.57
KY523104.1_AUL78032.1_MF405918.2_QKU33968.1_FTSJ-LIKE_METHYL_TRANSFERASE_CDS	0.25	0.37	0.34	0.16	0.19	0.37	0.37	0.17	0.49	0.45	0.14
KY523104.1_AUL78033.1_MF405918.2_QKU33969.1_HYPOTHETICAL_PROTEIN_CDS	0.2	0.12	0.13	0.01	0	0.36	0.23	0.02	0.09	0.35	0.17
KY523104.1_AUL78034.1_MF405918.2_QKU33970.1_ENDONUCLEASE_OF_THE_XPG_FAMILY_CDS	0.38	0.43	0.3	0.24	0.1	0.47	0.51	0.18	0.45	0.33	0.45
KY523104.1_AUL78035.1_MF405918.2_QKU33971.1_PUTATIVE_ORFAN_CDS	0.38	0.65	0.41	0.22	0.18	0.31	0.31	0.17	0.42	0.21	0.31

KY523104.1_AUL78036.1_MF405918.2_QKU33972.1_HYPOTHETICAL_PROTEIN_CDS	0.29	0.45	0.44	0.21	0.55	0.33	0.23	0.17	0.3	0.46	0.31
KY523104.1_AUL78037.1_MF405918.2_QKU33973.1_PUTATIVE_ORFAN_CDS	0.21	0.25	0.3	0.04	0	0.44	0.57	0.25	0.38	0.33	0.13
KY523104.1_AUL78038.1_MF405918.2_AUL79329.2_HYPOTHETICAL_PROTEIN_CDS	0.4	0.32	0.34	0.01	0.39	0.44	0.09	0.2	0.56	0.43	0.32
KY523104.1_AUL78039.1_MF405918.2_QKU33974.1_PUTATIVE_ORFAN_CDS	0.4	0.66	0.39	0.33	0	0.4	0.24	0.27	0.48	0.36	0.37
KY523104.1_AUL78040.1_MF405918.2_QKU33975.1_UBIQUITIN_DOMAIN-CONTAINING_PROTEIN_CDS	0.65	0.13	0.22	0.37	0.03	0.34	0.34	0.05	0.46	0.4	0.57
KY523104.1_AUL78041.1_MF405918.2_QKU33976.1_HNH_ENDONUCLEASE_CDS	0.36	0.5	0.36	0.19	0.25	0.39	0.44	0.18	0.45	0.36	0.42
KY523104.1_AUL78042.1_MF405918.2_QKU33977.1_PUTATIVE_ORFAN_CDS	0.23	0.39	0.36	0.13	0.61	0.35	0.25	0.27	0.1	0.45	0.29
KY523104.1_AUL78043.1_MF405918.2_QKU33978.1_PUTATIVE_ORFAN_CDS	0.2	0.28	0.31	0.1	0	0.2	0.26	0.08	0.3	0.44	0.13
KY523104.1_AUL78044.1_MF405918.2_QKU33979.1_PUTATIVE_ENDONUCLEASE/EXONUCLEASE/PHOSPHATASE_CDS	0.3	0.14	0.31	0.08	0.41	0.3	0.57	0.19	0.36	0.24	0.26
KY523104.1_AUL78045.1_MF405918.2_QKU33980.1_HYPOTHETICAL_PROTEIN_CDS	0.66	0.52	0.25	0.05	0.69	0.95	0.21	0.31	0.52	0.92	0.47
KY523104.1_AUL78046.1_MF405918.2_QKU33981.1_HYPOTHETICAL_PROTEIN_CDS	0.54	0.53	0.33	0.25	0.09	0.41	0.49	0.82	0.55	0.09	0.48
KY523104.1_AUL78047.1_MF405918.2_QKU33982.1_PUTATIVE_HEAT_SHOCK_70_KDA_PROTEIN_CDS	0.4	0.52	0.38	0.19	0.41	0.53	0.48	0.41	0.49	0.44	0.39
KY523104.1_AUL78048.1_MF405918.2_QKU33983.1_PUTATIVE_ORFAN_CDS	0.05	0.03	0.03	0	0.01	0.04	0.39	0.02	0.05	0.15	0.19
KY523104.1_AUL78048.1_MF405918.2_QKU33983.1_PUTATIVE_ORFAN_CDS	0.18	0.21	0.13	0.01	0.35	0.09	0.24	0.03	0.05	0.28	0.22
KY523104.1_AUL78047.1_MF405918.2_QKU33982.1_PUTATIVE_HEAT_SHOCK_70_KDA_PROTEIN_CDS	0.11	0.08	0.08	0	0.04	0.05	0.12	0.02	0.02	0.18	0.14
KY523104.1_AUL78049.1_MF405918.2_QKU33984.1_PUTATIVE_HEAT_SHOCK_70_KDA_PROTEIN_CDS	0.39	0.08	0.36	0.08	0	0.5	0.6	0.12	0.56	0.6	0.28
KY523104.1_AUL78050.1_MF405918.2_QKU33985.1_MG499_PROTEIN_CDS	0.44	0.34	0.22	0.05	0.29	0.23	0.61	0.35	0.38	0.13	0.49
KY523104.1_AUL78051.1_MF405918.2_QKU33986.1_HYPOTHETICAL_PROTEIN_CDS	0.11	0.03	0.12	0	0	0.05	0.01	0	0.73	0.38	0.03
KY523104.1_AUL78052.1_MF405918.2_QKU33987.1_PUTATIVE_SET_DOMAIN-CONTAINING_PROTEIN_CDS	0.41	0.48	0.24	0.05	0.57	0.18	0.62	0.23	0.35	0.06	0.46
KY523104.1_AUL78053.1_MF405918.2_QKU33988.1_PUTATIVE_ORFAN_CDS	0.31	0.61	0.45	0.22	0.02	0.33	0.31	0.24	0.48	0.33	0.2
KY523104.1_AUL78054.1_MF405918.2_QKU33989.1_REPLICATION_FACTOR_C_SMALL_SUBUNIT_CDS	0.39	0.27	0.22	0.07	0	0.24	0.59	0.19	0.41	0.11	0.39
KY523104.1_AUL78055.1_MF405918.2_QKU33990.1_HYPOTHETICAL_PROTEIN_CDS	0.22	0.08	0.3	0.06	0.01	0.51	0.24	0.42	0.48	0.4	0.11
KY523104.1_AUL78056.1_MF405918.2_QKU33991.1_MG497_PROTEIN_CDS	0.47	0.44	0.29	0.31	0.47	0.37	0.44	0.32	0.47	0.46	0.47
KY523104.1_AUL78057.1_MF405918.2_QKU33992.1_HYPOTHETICAL_PROTEIN_CDS	0.76	0.6	0.37	0.27	0.42	0.38	0.39	0.06	0.43	0.56	0.53
KY523104.1_AUL78058.1_MF405918.2_QKU33993.1_PUTATIVE_ORFAN_CDS	0.3	0.07	0.18	0.04	0.26	0.19	0.09	0.05	0.12	0.35	0.44
KY523104.1_AUL78059.1_MF405918.2_QKU33994.1_HYPOTHETICAL_PROTEIN_CDS	0.41	0.69	0.39	0.4	0.42	0.44	0.38	0.58	0.43	0.1	0.43
KY523104.1_AUL78060.1_MF405918.2_QKU33995.1_HYPOTHETICAL_PROTEIN_CDS	0.38	0.65	0.41	0.26	0.01	0.3	0.33	0.19	0.43	0.18	0.22
KY523104.1_AUL78061.1_MF405918.2_QKU33996.1_HYPOTHETICAL_PROTEIN_CDS	0.4	0.65	0.4	0.26	0.13	0.4	0.37	0.31	0.43	0.22	0.38
KY523104.1_AUL78062.1_MF405918.2_QKU33997.1_HYPOTHETICAL_PROTEIN_CDS	0.4	0.29	0.31	0.24	0.1	0.37	0.22	0.33	0.48	0.39	0.44
KY523104.1_AUL78063.1_MF405918.2_QKU33998.1_SERINE_THREONINE-PROTEIN_KINASE_CDS	0.37	0.36	0.36	0.59	0.31	0.34	0.23	0.25	0.53	0.57	0.31
KY523104.1_AUL78064.1_MF405918.2_QKU33999.1_PUTATIVE_ORFAN_CDS	0.24	0.24	0.15	0.04	0.3	0.13	0.15	0.02	0.08	0.39	0.24
KY523104.1_AUL78065.1_MF405918.2_QKU34000.1_PUTATIVE_ORFAN_CDS	0.4	0.07	0.17	0	0	0.36	0.59	0.08	0.37	0.12	0.41
KY523104.1_AUL78066.1_MF405918.2_QKU34001.1_HYPOTHETICAL_PROTEIN_CDS	0.59	0.19	0.31	0.2	0.04	0.27	0.31	0.17	0.07	0.39	0.44
KY523104.1_AUL78067.1_MF405918.2_QKU34002.1_HYPOTHETICAL_PROTEIN_CDS	0.4	0.64	0.35	0.37	0.46	0.4	0.44	0.29	0.54	0.34	0.43
KY523104.1_AUL78068.1_MF405918.2_QKU34003.1_HYPOTHETICAL_PROTEIN_CDS	0.38	0.5	0.34	0.29	0.24	0.51	0.37	0.18	0.47	0.44	0.42
KY523104.1_AUL78069.1_MF405918.2_QKU34004.1_ALPHA/BETA_HYDROLASE_FAMILY_PROTEIN_CDS	0.27	0.18	0.22	0.12	0.05	0.25	0.37	0.07	0.06	0.4	0.38
KY523104.1_AUL78070.1_MF405918.2_QKU34005.1_ALPHA/BETA_HYDROLASE_FAMILY_PROTEIN_CDS	0.51	0.52	0.37	0.18	0.17	0.35	0.14	0.06	0.3	0.39	0.44
KY523104.1_AUL78071.1_MF405918.2_QKU34006.1_PUTATIVE_RNA_METHYLTRANSFERASE_CDS	0.23	0.19	0.17	0.33	0	0.64	0.16	0.06	0.51	0.27	0.17
KY523104.1_AUL78072.1_MF405918.2_QKU34007.1_2OG-FE(II)_OXYGENASE_CDS	0.28	0.32	0.25	0.04	0	0.32	0.58	0.22	0.28	0.23	0.23
KY523104.1_AUL78073.1_MF405918.2_QKU34008.1_HYPOTHETICAL_PROTEIN_CDS	0.62	0.28	0.33	0.32	0.05	0.34	0.34	0.1	0.43	0.42	0.47
KY523104.1_AUL78074.1_MF405918.2_QKU34009.1_MGC52527_PROTEIN_CDS	0.4	0.59	0.41	0.1	0.21	0.38	0.56	0.56	0.36	0.23	0.36
KY523104.1_AUL78075.1_MF405918.2_QKU34010.1_RNA_METHYLTRANSFERASE_CDS	0.3	0.56	0.4	0.24	0.04	0.34	0.26	0.16	0.51	0.49	0.11

KY523104.1_AUL78076.1_MF405918.2_QKU34011.1_PUTATIVE_ORFAN_CDS	0.1	0.09	0.25	0.01	0.02	0.13	0.22	0.06	0.5	0.28	0.28
KY523104.1_AUL78077.1_MF405918.2_QKU34012.1_YALI0E31691P_CDS	0.26	0.05	0.27	0.06	0.41	0.45	0.24	0.11	0.26	0.42	0.25
KY523104.1_AUL78078.1_MF405918.2_QKU34013.1_PUTATIVE_PAPAIN-LIKE_CYSTEINE_PEPTIDASE_CDS	0.22	0.2	0.2	0.04	0.02	0.12	0.25	0.04	0.26	0.44	0.16
KY523104.1_AUL78079.1_MF405918.2_QKU34014.1_HYPOTHETICAL_PROTEIN_CDS	0.25	0.57	0.47	0.13	0.38	0.42	0.37	0.32	0.37	0.28	0.22
KY523104.1_AUL78080.1_MF405918.2_QKU34015.1_HYPOTHETICAL_PROTEIN_CDS	0.24	0.32	0.23	0.1	0.43	0.18	0.21	0.06	0.05	0.39	0.24
KY523104.1_AUL78081.1_MF405918.2_QKU34016.1_HYPOTHETICAL_PROTEIN_CDS	0.45	0.13	0.28	0.42	0.05	0.32	0.18	0.09	0.46	0.44	0.47
KY523104.1_AUL78082.1_MF405918.2_QKU34017.1_MAJOR_CORE_PROTEIN_CDS	0.4	0.35	0.36	0	0.19	0.55	0.4	0.38	0.63	0.58	0.31
KY523104.1_AUL78083.1_MF405918.2_QKU34018.1_PUTATIVE_ORFAN_CDS	0.28	0.06	0.22	0.42	0.03	0.29	0.16	0.14	0.1	0.42	0.4
KY523104.1_AUL78084.1_MF405918.2_QKU34019.1_PUTATIVE_ORFAN_CDS	0.14	0.35	0.15	0.04	0	0.16	0.17	0.01	0.12	0.41	0.08
KY523104.1_AUL78085.1_MF405918.2_QKU34020.1_MG482_PROTEIN_CDS	0.43	0.55	0.28	0.28	0.16	0.47	0.55	0.3	0.5	0.32	0.48
KY523104.1_AUL78086.1_MF405918.2_AUL79377.2_PUTATIVE_ORFAN_CDS	0.4	0.79	0.24	0.15	0.18	0.05	0.14	0.01	0.4	0	0.04
KY523104.1_AUL78087.1_MF405918.2_QKU34021.1_HYPOTHETICAL_PROTEIN_CDS	0.27	0.18	0.26	0.11	0	0.26	0.56	0.14	0.39	0.29	0.36
KY523104.1_AUL78088.1_MF405918.2_QKU34022.1_PUTATIVE_ORFAN_CDS	0.27	0.44	0.31	0.17	0.02	0.29	0.41	0.2	0.26	0.39	0.35
KY523104.1_AUL78089.1_MF405918.2_QKU34023.1_HYPOTHETICAL_PROTEIN_CDS	0.24	0.25	0.22	0.08	0.26	0.26	0.18	0.05	0.22	0.45	0.19
KY523104.1_AUL78090.1_MF405918.2_QKU34024.1_PUTATIVE_ORFAN_CDS	0.41	0.53	0.42	0.15	0.19	0.4	0.11	0.18	0.18	0.35	0.43
KY523104.1_AUL78091.1_MF405918.2_QKU34025.1_HYPOTHETICAL_PROTEIN_CDS	0.25	0.62	0.4	0.14	0.15	0.32	0.41	0.2	0.38	0.22	0.14
KY523104.1_AUL78092.1_MF405918.2_QKU34026.1_HYPOTHETICAL_PROTEIN_CDS	0.61	0.69	0.46	0.23	0.09	0.46	0.25	0.22	0.45	0.35	0.5
KY523104.1_AUL78093.1_MF405918.2_QKU34027.1_HYPOTHETICAL_PROTEIN_CDS	0.56	0.27	0.47	0.39	0.23	0.39	0.36	0.3	0.4	0.41	0.47
KY523104.1_AUL78094.1_MF405918.2_QKU34028.1_MG473_PROTEIN_CDS	0.3	0.1	0.18	0	0	0.34	0.58	0.09	0.37	0.18	0.14
KY523104.1_AUL78095.1_MF405918.2_QKU34029.1_HYPOTHETICAL_PROTEIN_CDS	0.3	0.49	0.46	0.15	0.59	0.39	0.11	0.43	0.32	0.41	0.24
KY523104.1_AUL78096.1_MF405918.2_QKU34030.1_HYPOTHETICAL_PROTEIN_CDS	0.4	0.21	0.38	0.7	0.49	0.39	0.37	0.51	0.42	0.35	0.45
KY523104.1_AUL78097.1_MF405918.2_QKU34031.1_CATALASE_HPII_CDS	0.4	0.03	0.41	0	0.99	0.98	0.58	0.11	0.15	0.92	0.25
KY523104.1_AUL78098.1_MF405918.2_QKU34032.1_ENDONUCLEASE_VIII-LIKE_PROTEIN_CDS	0.25	0.1	0.37	0.13	0.39	0.58	0.44	0.43	0.24	0.37	0.12
KY523104.1_AUL78099.1_MF405918.2_QKU34033.1_HYPOTHETICAL_PROTEIN_CDS	0.22	0.35	0.32	0.03	0	0.27	0.55	0.5	0.38	0.39	0.18
KY523104.1_AUL78100.1_MF405918.2_QKU34034.1_HYPOTHETICAL_PROTEIN_CDS	0.77	0.36	0.34	0.29	0.43	0.32	0.1	0.12	0.46	0.46	0.47
KY523104.1_AUL78101.1_MF405918.2_QKU34035.1_HYPOTHETICAL_PROTEIN_CDS	0.4	0.7	0.3	0.3	0.01	0.59	0.47	0.45	0.53	0.34	0.42
KY523104.1_AUL78102.1_MF405918.2_QKU34036.1_HYPOTHETICAL_PROTEIN_CDS	0.3	0.57	0.43	0.19	0.02	0.4	0.38	0.48	0.45	0.31	0.27
KY523104.1_AUL78103.1_MF405918.2_QKU34037.1_PUTATIVE_PEPTIDASE_INHIBITOR_15-LIKE_ISOFORM_X2_CDS	0.19	0.09	0.21	0.03	0.18	0.13	0.15	0.34	0.48	0.37	0.19
KY523104.1_AUL78104.1_MF405918.2_QKU34038.1_PUTATIVE_ORFAN_CDS	0.57	0.09	0.3	0.12	0.48	0.29	0.57	0.46	0.3	0.32	0.48
KY523104.1_AUL78105.1_MF405918.2_QKU34039.1_HYPOTHETICAL_PROTEIN_CDS	0.54	0.56	0.16	0.08	0.4	0.65	0.59	0.13	0.36	0.26	0.52
KY523104.1_AUL78106.1_MF405918.2_QKU34040.1_PUTATIVE_ORFAN_CDS	0.28	0.14	0.22	0.07	0.44	0.28	0.21	0.09	0.08	0.4	0.34
KY523104.1_AUL78107.1_MF405918.2_QKU34041.1_HYPOTHETICAL_PROTEIN_CDS	0.09	0.02	0.13	0	0	0.05	0.18	0.52	0.04	0.25	0.16
KY523104.1_AUL78108.1_MF405918.2_QKU34042.1_HYPOTHETICAL_PROTEIN_CDS	0.25	0.38	0.26	0.1	0.01	0.33	0.15	0.04	0.08	0.38	0.35
KY523104.1_AUL78109.1_MF405918.2_QKU34043.1_DNA_TOPOISOMERASE_1B_CDS	0.29	0.47	0.36	0.21	0	0.32	0.39	0.26	0.49	0.41	0.27
KY523104.1_AUL78110.1_MF405918.2_QKU34044.1_UDP-N-ACETYLGLUCOSAMINE_PYROPHOSPHORYLASE_CDS	0.36	0.58	0.47	0.25	0.38	0.43	0.35	0.36	0.4	0.3	0.35
KY523104.1_AUL78111.1_MF405918.2_QKU34045.1_CYCLOPROPANE_FATTY_ACYL_PHOSPHOLIPID_SYNTHASE_CDS	0.4	0.19	0.33	0.06	0.62	0.42	0.52	0.41	0.37	0.61	0.35
KY523104.1_AUL78112.1_MF405918.2_QKU34046.1_HYPOTHETICAL_PROTEIN_CDS	0.18	0.15	0.32	0.05	0.29	0.35	0.44	0.08	0.18	0.41	0.08
KY523104.1_AUL78113.1_MF405918.2_QKU34047.1_HYPOTHETICAL_PROTEIN_CDS	0.17	0.34	0.16	0.05	0.02	0.17	0.32	0.03	0.05	0.37	0.13
KY523104.1_AUL78114.1_MF405918.2_QKU34048.1_PUTATIVE_ORFAN_CDS	0.22	0.42	0.41	0.1	0	0.52	0.46	0.34	0.27	0.4	0.17
KY523104.1_AUL78115.1_MF405918.2_QKU34049.1_DESATURASE_CDS	0.81	0.19	0.31	0.25	0.06	0.59	0.24	0.07	0.46	0.29	0.56
KY523104.1_AUL78116.1_MF405918.2_QKU34050.1_HYPOTHETICAL_PROTEIN_CDS	0.34	0.07	0.22	0.07	0.28	0.18	0.07	0.14	0.31	0.38	0.42
KY523104.1_AUL78117.1_MF405918.2_QKU34051.1_PUTATIVE_DUAL_SPECIFICITY_PROTEIN_PHOSPHATASE_14-LIKE_C	0.23	0.18	0.26	0.02	0.03	0.19	0.48	0.31	0.03	0.35	0.2

KY523104.1_AUL78118.1_MF405918.2_QKU34052.1_HYPOTHETICAL_PROTEIN_CDS	0.4	0.28	0.24	0.21	0.62	0.32	0.28	0.39	0.55	0.69	0.43
KY523104.1_AUL78119.1_MF405918.2_QKU34053.1_EXTRACELLULAR_LIGAND-BINDING_RECEPTOR_CDS	0.38	0.31	0.35	0.24	0.52	0.42	0.33	0.44	0.49	0.59	0.37
KY523104.1_AUL78120.1_MF405918.2_QKU34054.1_PUTATIVE_ORFAN_CDS	0.12	0.11	0.11	0	0	0.24	0.18	0.01	0.07	0.27	0.09
KY523104.1_AUL78121.1_MF405918.2_QKU34055.1_LEUCINE_RICH_REPEAT_PROTEIN_CDS	0.56	0.14	0.13	0.02	0.21	0.19	0.62	0.03	0.39	0.04	0.53
KY523104.1_AUL78122.1_MF405918.2_QKU34056.1_HYPOTHETICAL_PROTEIN_CDS	0.33	0.25	0.32	0.36	0.42	0.39	0.19	0.13	0.36	0.44	0.42
KY523104.1_AUL78123.1_MF405918.2_QKU34057.1_HYPOTHETICAL_PROTEIN_CDS	0.56	0.61	0.15	0.29	0.09	0.61	0.56	0.06	0.5	0.32	0.54
KY523104.1_AUL78124.1_MF405918.2_QKU34058.1_PUTATIVE_ORFAN_CDS	0.21	0.16	0.23	0.06	0	0.5	0.21	0.12	0.22	0.36	0.1
KY523104.1_AUL78125.1_MF405918.2_QKU34059.1_HYPOTHETICAL_PROTEIN_CDS	0.63	0.36	0.23	0.53	0.56	0.49	0.47	0.26	0.43	0.62	0.51
KY523104.1_AUL78126.1_MF405918.2_QKU34060.1_ERV1_/_ALR_FAMILY_PROTEIN_CDS	0.28	0.43	0.29	0.13	0.29	0.27	0.16	0.1	0.05	0.37	0.38
KY523104.1_AUL78127.1_MF405918.2_QKU34061.1_HYPOTHETICAL_PROTEIN_CDS	0.24	0.15	0.27	0.04	0.01	0.29	0.53	0.15	0.12	0.31	0.22
KY523104.1_AUL78128.1_MF405918.2_QKU34062.1_HYPOTHETICAL_PROTEIN_CDS	0.21	0.06	0.38	0.01	0.01	0.42	0.52	0.57	0.47	0.44	0.11
KY523104.1_AUL78129.1_MF405918.2_QKU34063.1_PUTATIVE_ORFAN_CDS	0.27	0.55	0.43	0.15	0.34	0.4	0.36	0.27	0.31	0.33	0.29
KY523104.1_AUL78130.1_MF405918.2_QKU34064.1_PUTATIVE_ORFAN_CDS	0.45	0.14	0.35	0.02	0.01	0.19	0.01	0.55	0.16	0.21	0.42
KY523104.1_AUL78131.1_MF405918.2_QKU34065.1_HYPOTHETICAL_PROTEIN_CDS	0.57	0.44	0.35	0.26	0.62	0.34	0.31	0.41	0.39	0.6	0.45
KY523104.1_AUL78132.1_MF405918.2_QKU34066.1_TRNA_NUCLEOTIDYLTRANSFERASE/POLY(A)_POLYMERASE_CDS	0.38	0.39	0.33	0.22	0.35	0.34	0.32	0.29	0.38	0.38	0.43
KY523104.1_AUL78133.1_MF405918.2_QKU34067.1_PUTATIVE_ORFAN_CDS	0.21	0.09	0.35	0.02	0.69	0.3	0.36	0.24	0.31	0.43	0.05
KY523104.1_AUL78134.1_MF405918.2_QKU34068.1_GLYOXALASE_CDS	0.17	0.4	0.36	0.12	0	0.35	0.18	0.03	0.21	0.4	0.12
KY523104.1_AUL78135.1_MF405918.2_QKU34069.1_HYPOTHETICAL_PROTEIN_CDS	0.4	0.27	0.53	0.24	0.15	0.49	0.4	0.61	0.47	0.37	0.43
KY523104.1_AUL78136.1_MF405918.2_QKU34070.1_PUTATIVE_ORFAN_CDS	0.39	0.28	0.42	0.25	0.11	0.21	0.61	0.2	0.39	0.06	0.36
KY523104.1_AUL78137.1_MF405918.2_QKU34071.1_PUTATIVE_ORFAN_CDS	0.47	0.08	0.4	0	0.65	0.32	0.03	0.31	0.08	0.45	0.37
KY523104.1_AUL78138.1_MF405918.2_QKU34072.1_POLYKETIDE_CYCLASE_CDS	0.24	0.16	0.29	0.04	0.59	0.35	0.31	0.16	0.04	0.38	0.16
KY523104.1_AUL78139.1_MF405918.2_QKU34073.1_HYPOTHETICAL_PROTEIN_CDS	0.41	0.09	0.22	0.25	0.63	0.42	0.31	0.46	0.58	0.65	0.43
KY523104.1_AUL78140.1_MF405918.2_QKU34074.1_PUTATIVE_ORFAN_CDS	0.19	0.2	0.21	0.02	0	0.11	0.27	0.04	0.21	0.41	0.13
KY523104.1_AUL78141.1_MF405918.2_QKU34075.1_PUTATIVE_ORFAN_CDS	0.14	0.21	0.14	0.01	0	0.07	0.28	0.01	0.05	0.37	0.07
KY523104.1_AUL78142.1_MF405918.2_QKU34076.1_PUTATIVE_ORFAN_CDS	0.14	0.1	0.21	0	0.02	0.17	0.56	0.2	0.06	0.32	0.05
KY523104.1_AUL78143.1_MF405918.2_QKU34077.1_HYPOTHETICAL_PROTEIN_CDS	0.31	0.52	0.38	0.16	0.26	0.48	0.31	0.26	0.51	0.56	0.14
KY523104.1_AUL78144.1_MF405918.2_QKU34078.1_PUTATIVE_ORFAN_CDS	0.17	0.22	0.12	0.02	0	0.06	0.13	0.01	0.07	0.32	0.14
KY523104.1_AUL78145.1_MF405918.2_AUL79435.2_HYPOTHETICAL_PROTEIN_CDS	0.83	0.07	0.34	0.26	0.05	0.38	0.29	0.5	0.46	0.26	0.62
KY523104.1_AUL78146.1_MF405918.2_QKU34079.1_HYPOTHETICAL_PROTEIN_CDS	0.73	0.07	0.28	0.39	0.04	0.38	0.13	0.12	0.46	0.33	0.58
KY523104.1_AUL78147.1_MF405918.2_QKU34080.1_CAPSID_PROTEIN_1_CDS	0.34	0.44	0.36	0.1	0.01	0.4	0.41	0.55	0.44	0.3	0.37
KY523104.1_AUL78148.1_MF405918.2_QKU34081.1_HYPOTHETICAL_PROTEIN_CDS	0.69	0.2	0.41	0.34	0.36	0.37	0.24	0.11	0.44	0.43	0.49
KY523104.1_AUL78149.1_MF405918.2_QKU34082.1_PUTATIVE_ORFAN_CDS	0.4	0.01	0.28	0	0	0.17	0.01	0.12	0.55	0.2	0.45
KY523104.1_AUL78150.1_MF405918.2_QKU34083.1_HYPOTHETICAL_PROTEIN_CDS	0.3	0.35	0.57	0.23	0.57	0.47	0.32	0.46	0.22	0.54	0.28
KY523104.1_AUL78151.1_MF405918.2_QKU34084.1_MG462_PROTEIN_CDS	0.85	0.21	0.32	0.36	0.29	0.31	0.26	0.19	0.46	0.4	0.51
KY523104.1_AUL78152.1_MF405918.2_QKU34085.1_PUTATIVE_TRANSCRIPTION_FACTOR_CDS	0.27	0.1	0.19	0.03	0.39	0.16	0.26	0.08	0.07	0.39	0.27
KY523104.1_AUL78153.1_MF405918.2_QKU34086.1_EXONUCLEASE_CDS	0.34	0.22	0.36	0.3	0.33	0.38	0.14	0.51	0.24	0.34	0.43
KY523104.1_AUL78154.1_MF405918.2_QKU34087.1_PUTATIVE_HD_PHOSPHOHYDROLASE_CDS	0.38	0.4	0.35	0.31	0.46	0.37	0.23	0.17	0.34	0.41	0.44
KY523104.1_AUL78155.1_MF405918.2_QKU34088.1_HYPOTHETICAL_PROTEIN_CDS	0.23	0.38	0.33	0.12	0.42	0.32	0.17	0.35	0.23	0.39	0.21
KY523104.1_AUL78156.1_MF405918.2_QKU34089.1_PUTATIVE_ORFAN_CDS	0.53	0.76	0.29	0.45	0.05	0.55	0.27	0.09	0.51	0.25	0.59
KY523104.1_AUL78157.1_MF405918.2_QKU34090.1_HYPOTHETICAL_PROTEIN_CDS	0.34	0.27	0.36	0.09	0.11	0.29	0.37	0.56	0.31	0.3	0.4
KY523104.1_AUL78158.1_MF405918.2_QKU34091.1_HYPOTHETICAL_PROTEIN_CDS	0.62	0.43	0.34	0.21	0.61	0.41	0.45	0.46	0.34	0.53	0.47
KY523104.1_AUL78159.1_MF405918.2_QKU34092.1_PUTATIVE_ORFAN_CDS	0.3	0.23	0.25	0.13	0.02	0.28	0.55	0.19	0.21	0.29	0.42

KY523104.1_AUL78160.1_MF405918.2_QKU34093.1_PUTATIVE_ORFAN_CDS	0.02	0	0.04	0	0	0.05	0.01	0	0	0.13	0
KY523104.1_AUL78161.1_MF405918.2_QKU34094.1_MDLB_-_ABC-TYPE_MULTIDRUG_TRANSPORT_SYSTEM_CDS	0.28	0.21	0.35	0.1	0.53	0.42	0.52	0.3	0.33	0.41	0.24
KY523104.1_AUL78162.1_MF405918.2_QKU34095.1_HYPOTHETICAL_PROTEIN_CDS	0.84	0.01	0.27	0.34	0	0.38	0.12	0.06	0.45	0.3	0.59
KY523104.1_AUL78163.1_MF405918.2_QKU34096.1_REPEAT_PROTEIN_CDS	0.32	0.64	0.4	0.19	0.38	0.34	0.24	0.3	0.39	0.19	0.22
KY523104.1_AUL78164.1_MF405918.2_QKU34097.1_HYPOTHETICAL_PROTEIN_CDS	0.17	0.26	0.24	0.04	0	0.22	0.38	0.14	0.07	0.42	0.15
KY523104.1_AUL78165.1_MF405918.2_QKU34098.1_PUTATIVE_ORFAN_CDS	0.07	0.07	0.07	0	0.03	0.05	0.4	0.04	0.13	0.19	0.09
KY523104.1_AUL78166.1_MF405918.2_QKU34099.1_SERINE/THREONINE_PROTEIN_KINASE_CDS	0.38	0.11	0.29	0.04	0.58	0.35	0.58	0.3	0.3	0.22	0.26
KY523104.1_AUL78167.1_MF405918.2_QKU34100.1_HISTIDINE_KINASE_CDS	0.68	0.26	0.37	0.3	0.42	0.58	0.25	0.29	0.44	0.37	0.47
KY523104.1_AUL78168.1_MF405918.2_QKU34101.1_HISTIDINE_PROTEIN_KINASE_SLN1_CDS	0.23	0.14	0.38	0.01	0.72	0.32	0.29	0.26	0.21	0.47	0.11
KY523104.1_AUL78167.1_MF405918.2_QKU34100.1_HISTIDINE_KINASE_CDS	0.97	0.13	0.15	0	0	0.06	0.19	0.01	0.01	0.31	0.39
KY523104.1_AUL78169.1_MF405918.2_QKU34102.1_PAS_DOMAIN-CONTAINING_TWO-COMPONENT_SYSTEM_SENSOR	0.41	0.69	0.39	0.2	0.3	0.31	0.52	0.35	0.39	0.03	0.33
KY523104.1_AUL78170.1_MF405918.2_QKU34103.1_HYPOTHETICAL_PROTEIN_CDS	0.4	0.67	0.41	0.28	0.2	0.26	0.27	0.16	0.42	0.12	0.2
KY523104.1_AUL78171.1_MF405918.2_QKU34104.1_A32-LIKE_PACKAGING_ATPASE_CDS	0.21	0.41	0.26	0.12	0	0.26	0.36	0.13	0.16	0.39	0.23
KY523104.1_AUL78172.1_MF405918.2_QKU34105.1_PUTATIVE_ORFAN_CDS	0.37	0.71	0.43	0.15	0.01	0.22	0.05	0.12	0.41	0.21	0.28
KY523104.1_AUL78173.1_MF405918.2_QKU34106.1_HYPOTHETICAL_PROTEIN_CDS	0.41	0.69	0.46	0.08	0.06	0.32	0.55	0.54	0.46	0.01	0.21
KY523104.1_AUL78174.1_MF405918.2_QKU34107.1_HYPOTHETICAL_PROTEIN_CDS	0.22	0.18	0.29	0.05	0.61	0.3	0.37	0.12	0.04	0.4	0.14
KY523104.1_AUL78175.1_MF405918.2_AUL79466.2_PUTATIVE_ORFAN_CDS	0.14	0.17	0.09	0	0.01	0.08	0.32	0.01	0.04	0.25	0.12
KY523104.1_AUL78176.1_MF405918.2_QKU34108.1_PUTATIVE_TARTRATE-RESISTANT_ACID_PHOSPHATASE_TYPE_5_C	0.31	0.17	0.35	0.16	0.38	0.42	0.19	0.27	0.41	0.41	0.42
KY523104.1_AUL78177.1_MF405918.2_QKU34109.1_PUTATIVE_ORFAN_CDS	0.19	0.5	0.41	0.1	0	0.35	0.21	0.07	0.29	0.36	0.16
KY523104.1_AUL78178.1_MF405918.2_QKU34110.1_CAPSID_PROTEIN_CDS	0.45	0.25	0.25	0.31	0.31	0.72	0.47	0.38	0.44	0.51	0.45
KY523104.1_AUL78179.1_MF405918.2_QKU34111.1_REPEAT_PROTEIN_CDS	0.39	0.52	0.21	0	0	0.17	0.55	0.69	0.24	0.05	0.36
KY523104.1_AUL78180.1_MF405918.2_QKU34112.1_PUTATIVE_ORFAN_CDS	0.21	0.33	0.27	0.05	0	0.21	0.18	0.18	0.53	0.49	0.1
KY523104.1_AUL78181.1_MF405918.2_QKU34113.1_RNA_2_-_TPT1_FAMILY_PROTEIN_CDS	0.27	0.15	0.22	0.06	0.44	0.29	0.16	0.07	0.15	0.44	0.26
KY523104.1_AUL78182.1_MF405918.2_QKU34114.1_PUTATIVE_ORFAN_CDS	0.16	0.03	0.22	0	0	0.37	0.63	0.1	0.23	0.26	0.03
KY523104.1_AUL78183.1_MF405918.2_QKU34115.1_HYPOTHETICAL_PROTEIN_CDS	0.44	0.58	0.04	0.07	0	0.87	0.67	0.09	0.67	0.49	0.53
KY523104.1_AUL78184.1_MF405918.2_QKU34116.1_PUTATIVE_ORFAN_CDS	0.35	0.28	0.41	0.21	0.57	0.33	0.35	0.23	0.19	0.49	0.42
KY523104.1_AUL78185.1_MF405918.2_AUL79482.2_PUTATIVE_ORFAN_CDS	0.15	0.45	0.29	0.31	0.01	0.24	0.33	0.05	0.13	0.36	0.14
KY523104.1_AUL78186.1_MF405918.2_AUL79483.2_PUTATIVE_ORFAN_CDS	0.25	0.4	0.24	0.11	0.24	0.22	0.23	0.07	0.05	0.36	0.3
KY523104.1_AUL78187.1_MF405918.2_QKU34117.1_MG448_PROTEIN_CDS	0.59	0.28	0.32	0.3	0.14	0.43	0.39	0.07	0.42	0.45	0.49
KY523104.1_AUL78188.1_MF405918.2_QKU34118.1_PUTATIVE_ORFAN_CDS	0.28	0.04	0.14	0.03	0	0.1	0.04	0.02	0.05	0.35	0.49
KY523104.1_AUL78189.1_MF405918.2_QKU34119.1_HYPOTHETICAL_PROTEIN_CDS	0.28	0.21	0.27	0.18	0.36	0.28	0.3	0.1	0.19	0.42	0.4
KY523104.1_AUL78190.1_MF405918.2_QKU34120.1_SWARMING_MOTILITY_PROTEIN_YBIA_CDS	0.23	0.26	0.33	0.08	0	0.53	0.41	0.3	0.1	0.45	0.2
KY523104.1_AUL78191.1_MF405918.2_QKU34121.1_HYPOTHETICAL_PROTEIN_CDS	0.4	0.66	0.49	0.26	0.58	0.5	0.55	0.23	0.55	0.31	0.38
KY523104.1_AUL78192.1_MF405918.2_QKU34122.1_THIOREDOXIN_DOMAIN-CONTAINING_PROTEIN_CDS	0.85	0.55	0.35	0.28	0.08	0.44	0.15	0.04	0.44	0.41	0.55
KY523104.1_AUL78193.1_MF405918.2_QKU34123.1_ADP-RIBOSYLGLYCOHYDROLASE_CDS	0.34	0.33	0.36	0.21	0.41	0.42	0.4	0.33	0.51	0.5	0.34
KY523104.1_AUL78194.1_MF405918.2_QKU34124.1_PUTATIVE_J_DOMAIN-CONTAINING_PROTEIN_CDS	0.41	0.63	0.22	0	0.07	0.49	0.6	0.75	0.29	0.03	0.36
KY523104.1_AUL78195.1_MF405918.2_QKU34125.1_PUTATIVE_ORFAN_CDS	0.66	0.14	0.28	0.24	0.02	0.26	0.26	0.09	0.16	0.4	0.46
KY523104.1_AUL78196.1_MF405918.2_AUL79492.2_PUTATIVE_PATATIN-LIKE_PHOSPHOLIPASE_CDS	0.78	0.2	0.26	0.2	0.51	0.22	0.55	0.4	0.28	0.38	0.53
KY523104.1_AUL78197.1_MF405918.2_QKU34126.1_MG441_PROTEIN_CDS	0.38	0.05	0.22	0.13	0.01	0.36	0.19	0.04	0.19	0.41	0.44
KY523104.1_AUL78198.1_MF405918.2_QKU34127.1_HYPOTHETICAL_PROTEIN_CDS	0.25	0.44	0.32	0.16	0.01	0.27	0.22	0.08	0.39	0.4	0.28
KY523104.1_AUL78199.1_MF405918.2_QKU34128.1_HYPOTHETICAL_PROTEIN_CDS	0.41	0.54	0.11	0	0.43	0.19	0.71	0.36	0.39	0	0.42
KY523104.1_AUL78200.1_MF405918.2_QKU34129.1_PUTATIVE_ORFAN_CDS	0.27	0.52	0.29	0.09	0	0.25	0.56	0.36	0.4	0.23	0.22

KY523104.1_AUL78201.1_MF405918.2_QKU34130.1_PUTATIVE_VIRAL_TRANSCRIPTION_FACTOR_CDS	0.56	0.3	0.28	0.13	0.01	0.63	0.51	0.11	0.38	0.4	0.48
KY523104.1_AUL78202.1_MF405918.2_QKU34131.1_NUDIX_HYDROLASE_CDS	0.19	0.1	0.17	0.01	0.01	0.13	0.34	0.03	0.11	0.36	0.18
KY523104.1_AUL78203.1_MF405918.2_QKU34132.1_CELL_DIVISION_CYCLE_123_PROTEIN_CDS	0.82	0.25	0.3	0.36	0.02	0.3	0.05	0.07	0.45	0.39	0.53
KY523104.1_AUL78204.1_MF405918.2_QKU34133.1_HYPOTHETICAL_PROTEIN_CDS	0.35	0.16	0.33	0.11	0.01	0.32	0.54	0.07	0.39	0.53	0.41
KY523104.1_AUL78205.1_MF405918.2_QKU34134.1_MG437_PROTEIN_CDS	0.76	0.33	0.17	0.28	0.07	0.21	0.48	0.03	0.46	0.41	0.57
KY523104.1_AUL78206.1_MF405918.2_QKU34135.1_PUTATIVE_ORFAN_CDS	0.41	0.56	0.32	0.27	0.05	0.41	0.48	0.41	0.5	0.36	0.47
KY523104.1_AUL78207.1_MF405918.2_QKU34136.1_TATA-BOX-BINDING_PROTEIN-LIKE_PROTEIN_CDS	0.35	0.47	0.4	0.58	0.32	0.44	0.28	0.22	0.43	0.4	0.4
KY523104.1_AUL78208.1_MF405918.2_QKU34137.1_PUTATIVE_ORFAN_CDS	0.4	0.67	0.48	0.52	0.56	0.35	0.32	0.31	0.41	0.17	0.31
KY523104.1_AUL78209.1_MF405918.2_QKU34138.1_HYPOTHETICAL_PROTEIN_CDS	0.4	0.32	0.36	0.2	0.6	0.32	0.32	0.38	0.35	0.61	0.43
KY523104.1_AUL78209.1_MF405918.2_QKU34138.1_HYPOTHETICAL_PROTEIN_CDS	0.03	0	0.07	0	0	0.04	0.17	0.01	0.01	0.19	0.01
KY523104.1_AUL78210.1_MF405918.2_QKU34139.1_PUTATIVE_ORFAN_CDS	0.03	0	0.07	0	0	0.04	0.17	0.01	0.01	0.19	0.01
KY523104.1_AUL78211.1_MF405918.2_QKU34140.1_PUTATIVE_PORE_COAT_ASSEMBLY_FACTOR_CDS	0.4	0.72	0.32	0.21	0.16	0.43	0.57	0.47	0.42	0	0.4
KY523104.1_AUL78212.1_MF405918.2_QKU34141.1_HYPOTHETICAL_PROTEIN_CDS	0.19	0.35	0.34	0.04	0.05	0.24	0.13	0.5	0.17	0.24	0.22
KY523104.1_AUL78213.1_MF405918.2_QKU34142.1_HYPOTHETICAL_PROTEIN_CDS	0.19	0.2	0.12	0.02	0.01	0.06	0.2	0.02	0.09	0.29	0.21
KY523104.1_AUL78214.1_MF405918.2_QKU34143.1_HYPOTHETICAL_PROTEIN_CDS	0.25	0.3	0.23	0.14	0.02	0.26	0.42	0.1	0.12	0.39	0.34
KY523104.1_AUL78215.1_MF405918.2_QKU34144.1_PUTATIVE_ORFAN_CDS	0.89	0.09	0.25	0.32	0	0.29	0.33	0.06	0.38	0.37	0.54
KY523104.1_AUL78216.1_MF405918.2_QKU34145.1_MIMIVIRUS_TRANSLATION_INITIATION_FACTOR_4A_CDS	0.4	0.58	0.43	0.71	0.57	0.36	0.41	0.26	0.45	0.5	0.39
KY523104.1_AUL78217.1_MF405918.2_QKU34146.1_PHOSPHATIDYLSERINE_DECARBOXYLASE_CDS	0.4	0.67	0.42	0.34	0.4	0.37	0.41	0.4	0.42	0.15	0.37
KY523104.1_AUL78218.1_MF405918.2_QKU34147.1_HYPOTHETICAL_PROTEIN_CDS	0.57	0.08	0.26	0.21	0.02	0.34	0.47	0.08	0.15	0.45	0.47
KY523104.1_AUL78219.1_MF405918.2_QKU34148.1_HYPOTHETICAL_PROTEIN_CDS	0.35	0.41	0.47	0.1	0.61	0.35	0.06	0.31	0.29	0.6	0.35
KY523104.1_AUL78220.1_MF405918.2_QKU34149.1_PUTATIVE_UBIQUITIN-CONJUGATING_ENZYME_E2_CDS	0.78	0.01	0.44	0.15	0.46	0.24	0.11	0.64	0.24	0.19	0.46
KY523104.1_AUL78221.1_MF405918.2_QKU34150.1_PUTATIVE_ORFAN_CDS	0.61	0.05	0.33	0.24	0.56	0.28	0.08	0.26	0.38	0.46	0.45
KY523104.1_AUL78222.1_MF405918.2_QKU34151.1_HYPOTHETICAL_PROTEIN_CDS	0.19	0.03	0.25	0.04	0.33	0.52	0.17	0.07	0.48	0.4	0.08
KY523104.1_AUL78223.1_MF405918.2_QKU34152.1_PUTATIVE_ORFAN_CDS	0.38	0.44	0.41	0.7	0.44	0.39	0.23	0.35	0.41	0.38	0.41
KY523104.1_AUL78224.1_MF405918.2_QKU34153.1_HYPOTHETICAL_PROTEIN_CDS	0.05	0.11	0.07	0	0	0.05	0.16	0.01	0.01	0.2	0.01
KY523104.1_AUL78225.1_MF405918.2_QKU34154.1_HYPOTHETICAL_PROTEIN_CDS	0.21	0.31	0.22	0.06	0.01	0.15	0.13	0.11	0.07	0.33	0.3
KY523104.1_AUL78226.1_MF405918.2_QKU34155.1_MG424_PROTEIN_CDS	0.25	0.15	0.23	0	0	0.34	0.58	0.32	0.27	0.22	0.17
KY523104.1_AUL78227.1_MF405918.2_QKU34156.1_MEMBER_RAS_ONCOGENE_FAMILY_RAB32_CDS	0.2	0.04	0.3	0	0.48	0.32	0.58	0.16	0.27	0.26	0.09
KY523104.1_AUL78228.1_MF405918.2_QKU34157.1_PUTATIVE_ORFAN_CDS	0.28	0.46	0.44	0.63	0.02	0.4	0.42	0.3	0.35	0.35	0.25
KY523104.1_AUL78229.1_MF405918.2_QKU34158.1_PUTATIVE_ORFAN_CDS	0.3	0.18	0.31	0.36	0.23	0.37	0.33	0.09	0.3	0.44	0.42
KY523104.1_AUL78230.1_MF405918.2_QKU34159.1_PUTATIVE_ORFAN_CDS	0.19	0.11	0.39	0.01	0.66	0.55	0.14	0.17	0.1	0.38	0.05
KY523104.1_AUL78231.1_MF405918.2_QKU34160.1_THIOREDOXIN_REDUCTASE_CDS	0.4	0.35	0.3	0.03	0.14	0.15	0.44	0.15	0.6	0.46	0.43
KY523104.1_AUL78232.1_MF405918.2_QKU34161.1_HYPOTHETICAL_PROTEIN_CDS	0.42	0.78	0.38	0.18	0.01	0.18	0.05	0.06	0.42	0.07	0.14
KY523104.1_AUL78233.1_MF405918.2_QKU34162.1_MIMIVIRUS_TRANSLATION_FACTOR_SUI1-LIKE_2_CDS	0.19	0.31	0.16	0.05	0.01	0.12	0.37	0.05	0.09	0.39	0.15
KY523104.1_AUL78234.1_MF405918.2_QKU34163.1_HYPOTHETICAL_PROTEIN_CDS	0.29	0.29	0.38	0.03	0	0.52	0.36	0.29	0.54	0.61	0.13
KY523104.1_AUL78235.1_MF405918.2_QKU34164.1_HYPOTHETICAL_PROTEIN_CDS	0.18	0.27	0.14	0.36	0.01	0.1	0.37	0.03	0.03	0.32	0.15
KY523104.1_AUL78236.1_MF405918.2_QKU34165.1_PUTATIVE_ORFAN_CDS	0.69	0.12	0.26	0.38	0.21	0.32	0.35	0.07	0.45	0.41	0.52
KY523104.1_AUL78237.1_MF405918.2_QKU34166.1_HYPOTHETICAL_PROTEIN_CDS	0.56	0.29	0.3	0.23	0.45	0.62	0.3	0.22	0.43	0.49	0.45
KY523104.1_AUL78238.1_MF405918.2_QKU34167.1_MG419_PROTEIN_CDS	0.23	0.46	0.33	0.13	0.01	0.26	0.42	0.32	0.45	0.39	0.17
KY523104.1_AUL78239.1_MF405918.2_QKU34168.1_MG417_PROTEIN_CDS	0.33	0.19	0.35	0.33	0.45	0.32	0.48	0.16	0.21	0.45	0.43
KY523104.1_AUL78240.1_MF405918.2_QKU34169.1_HYPOTHETICAL_PROTEIN_CDS	0.26	0.33	0.25	0.12	0.17	0.35	0.38	0.1	0.07	0.42	0.32
KY523104.1_AUL78241.1_MF405918.2_QKU34170.1_PUTATIVE_ORFAN_CDS	0.51	0.19	0.3	0.34	0.26	0.35	0.35	0.1	0.44	0.45	0.47

KY523104.1_AUL78242.1_MF405918.2_QKU34171.1_MG415_PROTEIN_CDS	0.4	0.67	0.45	0.2	0.02	0.38	0.47	0.35	0.43	0.08	0.2
KY523104.1_AUL78243.1_MF405918.2_QKU34172.1_PUTATIVE_ORFAN_CDS	0.19	0.32	0.16	0.04	0.01	0.2	0.42	0.07	0.04	0.36	0.15
KY523104.1_AUL78244.1_MF405918.2_QKU34173.1_DNA_DIRECTED_RNA_POLYMERASE_SUBUNIT_CDS	0.6	0.11	0.3	0.31	0.02	0.42	0.32	0.07	0.42	0.41	0.47
KY523104.1_AUL78245.1_MF405918.2_QKU34174.1_HYPOTHETICAL_PROTEIN_CDS	0.31	0.52	0.23	0.06	0	0.32	0.57	0.31	0.33	0.21	0.35
KY523104.1_AUL78246.1_MF405918.2_QKU34175.1_HYPOTHETICAL_PROTEIN_CDS	0.47	0.68	0.17	0.04	0.09	0.97	0.61	0.44	0.43	0.08	0.53
KY523104.1_AUL78247.1_MF405918.2_QKU34176.1_HYPOTHETICAL_PROTEIN_CDS	0.16	0.39	0.29	0.05	0	0.38	0.47	0.4	0.18	0.38	0.15
KY523104.1_AUL78248.1_MF405918.2_QKU34177.1_HYPOTHETICAL_PROTEIN_CDS	0.4	0.72	0.48	0.52	0.66	0.37	0.47	0.27	0.4	0.04	0.24
KY523104.1_AUL78249.1_MF405918.2_QKU34178.1_GLUTAMINE-DEPENDENT_ASPARAGINE_SYNTHETASE_CDS	0.4	0.66	0.36	0.3	0.16	0.48	0.32	0.13	0.49	0.36	0.41
KY523104.1_AUL78250.1_MF405918.2_QKU34179.1_PUTATIVE_AAA_FAMILY_ATPASE_CDS	0.41	0.49	0.15	0.01	0.02	0.3	0.68	0.3	0.38	0.01	0.49
KY523104.1_AUL78251.1_MF405918.2_QKU34180.1_PUTATIVE_VIRION-ASSOCIATED_MEMBRANE_PROTEIN_CDS	0.35	0.33	0.42	0.8	0.52	0.4	0.29	0.31	0.36	0.48	0.38
KY523104.1_AUL78252.1_MF405918.2_QKU34181.1_HYPOTHETICAL_PROTEIN_CDS	0.3	0.35	0.33	0.18	0.37	0.33	0.46	0.27	0.33	0.38	0.38
KY523104.1_AUL78253.1_MF405918.2_QKU34182.1_PUTATIVE_ORFAN_CDS	0.09	0	0.34	0	0.71	0.42	0.11	0.1	0.07	0.4	0.01
KY523104.1_AUL78254.1_MF405918.2_QKU34183.1_HYPOTHETICAL_PROTEIN_CDS	0.18	0.34	0.15	0.05	0	0.09	0.24	0.01	0.07	0.37	0.12
KY523104.1_AUL78255.1_MF405918.2_QKU34184.1_REPEAT_PROTEIN_CDS	0.26	0.38	0.26	0.15	0.01	0.25	0.3	0.07	0.3	0.43	0.32
KY523104.1_AUL78256.1_MF405918.2_QKU34185.1_PUTATIVE_ORFAN_CDS	0.06	0.01	0.18	0	0.09	0.34	0.01	0.02	0.03	0.29	0.03
KY523104.1_AUL78257.1_MF405918.2_QKU34186.1_HYPOTHETICAL_PROTEIN_CDS	0.29	0.07	0.19	0.07	0.01	0.47	0.17	0.1	0.08	0.34	0.41
KY523104.1_AUL78258.1_MF405918.2_QKU34187.1_CATHEPSIN_B_-_CYSTEIN_PROTEASE_CDS	0.4	0.63	0.26	0.12	0.29	0.24	0.25	0.68	0.58	0.58	0.34
KY523104.1_AUL78259.1_MF405918.2_QKU34188.1_BIFUNCTIONAL_AAA_FAMILY_ATPASE_CHAPERONE/TRANSLOCASI	0.4	0.36	0.34	0.3	0.11	0.48	0.58	0.39	0.47	0.28	0.43
KY523104.1_AUL78260.1_MF405918.2_QKU34189.1_PUTATIVE_REPLICATION_FACTOR_C_SMALL_SUBUNIT_CDS	0.27	0.45	0.24	0.07	0	0.27	0.57	0.3	0.4	0.19	0.28
KY523104.1_AUL78261.1_MF405918.2_QKU34190.1_PUTATIVE_ORFAN_CDS	0.03	0.02	0.05	0	0	0.03	0.29	0.01	0.05	0.18	0.02
KY523104.1_AUL78262.1_MF405918.2_QKU34191.1_PUTATIVE_ORFAN_CDS	0.21	0.19	0.3	0.13	0	0.3	0.38	0.1	0.43	0.42	0.1
KY523104.1_AUL78263.1_MF405918.2_QKU34192.1_PUTATIVE_ORFAN_CDS	0.2	0.31	0.25	0.06	0.01	0.38	0.16	0.03	0.12	0.42	0.18
KY523104.1_AUL78264.1_MF405918.2_QKU34193.1_PUTATIVE_ORFAN_CDS	0.26	0.37	0.36	0.12	0	0.68	0.5	0.09	0.42	0.44	0.07
KY523104.1_AUL78265.1_MF405918.2_QKU34194.1_PUTATIVE_SYNTAXIN-7-LIKE_CDS	0.16	0.26	0.13	0.02	0	0.08	0.11	0	0.09	0.39	0.14
KY523104.1_AUL78266.1_MF405918.2_QKU34195.1_NUCLEOSIDE_TRIPHOSPHATE_PYROPHOSPHOHYDROLASE_CDS	0.4	0.44	0.47	0.03	0.68	0.33	0.35	0.51	0.29	0.66	0.36
KY523104.1_AUL78267.1_MF405918.2_QKU34196.1_2OG-FE(II)_OXYGENASE_CDS	0.25	0.21	0.53	0.1	0.26	0.19	0.14	0.16	0.04	0.41	0.29
KY523104.1_AUL78268.1_MF405918.2_QKU34197.1_MG403_PROTEIN_CDS	0.43	0.54	0.18	0.21	0.17	0.85	0.59	0.4	0.49	0.55	0.49
KY523104.1_AUL78269.1_MF405918.2_QKU34198.1_ARYLSULFATASE_CDS	0.56	0.39	0.39	0.34	0.37	0.38	0.2	0.14	0.46	0.44	0.48
KY523104.1_AUL78270.1_MF405918.2_QKU34199.1_HYPOTHETICAL_PROTEIN_CDS	0.4	0.71	0.44	0.86	0.04	0.32	0.51	0.43	0.45	0.03	0.28
KY523104.1_AUL78271.1_MF405918.2_QKU34200.1_PEPTIDASE_INHIBITOR_I9_CDS	0.34	0.56	0.46	0.25	0.27	0.45	0.43	0.31	0.45	0.33	0.35
KY523104.1_AUL78272.1_MF405918.2_QKU34201.1_PUTATIVE_ORFAN_CDS	0.72	0.24	0.26	0.17	0.08	0.24	0.08	0.04	0.21	0.41	0.45
KY523104.1_AUL78273.1_MF405918.2_QKU34202.1_PUTATIVE_ORFAN_CDS	0.33	0.4	0.33	0.08	0	0.57	0.3	0.29	0.41	0.52	0.39
KY523104.1_AUL78274.1_MF405918.2_QKU34203.1_PUTATIVE_ORFAN_CDS	0.64	0.66	0.37	0.34	0.08	0.47	0.31	0.13	0.47	0.4	0.52
KY523104.1_AUL78275.1_MF405918.2_QKU34204.1_PUTATIVE_OXOGLUTARATE/MALATE_CARRIER_PROTEIN_CDS	0.28	0.29	0.29	0.2	0.39	0.31	0.37	0.15	0.4	0.43	0.37
KY523104.1_AUL78276.1_MF405918.2_QKU34205.1_HYPOTHETICAL_PROTEIN_CDS	0.4	0.69	0.38	0.39	0.26	0.55	0.29	0.22	0.47	0.31	0.43
KY523104.1_AUL78277.1_MF405918.2_QKU34206.1_PUTATIVE_ORFAN_CDS	0.96	0.02	0.21	0.04	0	0.16	0.01	0.05	0.02	0.32	0.51
KY523104.1_AUL78278.1_MF405918.2_QKU34207.1_PUTATIVE_ANKYRIN_REPEAT_PROTEIN_CDS	0.42	0.62	0.22	0.03	0.34	0.55	0.69	0.34	0.68	0.06	0.52
KY523104.1_AUL78279.1_MF405918.2_QKU34208.1_PUTATIVE_ORFAN_CDS	0.29	0.19	0.26	0	0	0.14	0.53	0.65	0.23	0.2	0.26
KY523104.1_AUL78280.1_MF405918.2_QKU34209.1_HYPOTHETICAL_PROTEIN_CDS	0.66	0.05	0.22	0.07	0.05	0.25	0.04	0.04	0.04	0.36	0.46
KY523104.1_AUL78281.1_MF405918.2_QKU34210.1_PUTATIVE_PAN_DOMAIN-CONTAINING_PROTEIN_CDS	0.42	0.36	0.3	0.05	0.02	0.29	0.21	0.05	0.25	0.64	0.39
KY523104.1_AUL78282.1_MF405918.2_QKU34211.1_HYPOTHETICAL_PROTEIN_CDS	0.79	0.69	0.5	0.29	0.25	0.51	0.17	0.05	0.45	0.46	0.56
KY523104.1_AUL78283.1_MF405918.2_QKU34212.1_MG394_PROTEIN_CDS	0.34	0.24	0.33	0.23	0.1	0.32	0.23	0.34	0.27	0.39	0.43

KY523104.1_AUL78284.1_MF405918.2_QKU34213.1_PUTATIVE_ORFAN_CDS	0.26	0.2	0.32	0.1	0.54	0.31	0.28	0.21	0.27	0.47	0.31
KY523104.1_AUL78285.1_MF405918.2_QKU34214.1_PUTATIVE_ORFAN_CDS	0.13	0	0.2	0	0	0.06	0.07	0.01	0.73	0.37	0.07
KY523104.1_AUL78286.1_MF405918.2_QKU34215.1_HYPOTHETICAL_PROTEIN_CDS	0.26	0.13	0.17	0.03	0.08	0.12	0.23	0.03	0.28	0.38	0.29
KY523104.1_AUL78287.1_MF405918.2_QKU34216.1_HYPOTHETICAL_PROTEIN_CDS	0.4	0.76	0.24	0.57	0.03	0.68	0.57	0.3	0.48	0	0.44
KY523104.1_AUL78288.1_MF405918.2_QKU34217.1_HYPOTHETICAL_PROTEIN_CDS	0.15	0.4	0.28	0.06	0	0.41	0.23	0.03	0.09	0.39	0.13
KY523104.1_AUL78289.1_MF405918.2_QKU34218.1_HYPOTHETICAL_PROTEIN_CDS	0.11	0.43	0.13	0.01	0	0.06	0	0	0	0.3	0.06
KY523104.1_AUL78290.1_MF405918.2_QKU34219.1_HYPOTHETICAL_PROTEIN_CDS	0.39	0.26	0.32	0.25	0.35	0.37	0.32	0.27	0.49	0.46	0.44
KY523104.1_AUL78291.1_MF405918.2_QKU34220.1_HYPOTHETICAL_PROTEIN_CDS	0.21	0.32	0.3	0.02	0	0.25	0.58	0.5	0.28	0.28	0.18
KY523104.1_AUL78292.1_MF405918.2_QKU34221.1_HYPOTHETICAL_PROTEIN_CDS	0.28	0.19	0.39	0.06	0.64	0.38	0.42	0.32	0.23	0.47	0.24
KY523104.1_AUL78293.1_MF405918.2_QKU34222.1_PUTATIVE_DNA_POLYMERASE_SLIDING_CLAMP_CDS	0.35	0.5	0.37	0.13	0.29	0.32	0.54	0.35	0.45	0.23	0.37
KY523104.1_AUL78294.1_MF405918.2_QKU34223.1_HYPOTHETICAL_PROTEIN_CDS	0.81	0.02	0.28	0.32	0	0.3	0.16	0.13	0.44	0.35	0.53
KY523104.1_AUL78295.1_MF405918.2_QKU34224.1_HYPOTHETICAL_PROTEIN_CDS	0.31	0.6	0.41	0.13	0.04	0.39	0.39	0.27	0.39	0.27	0.33
KY523104.1_AUL78296.1_MF405918.2_QKU34225.1_MIMIVIRUS_TRANSLATION_INITIATION_FACTOR_4E,_2_CDS	0.23	0.53	0.38	0.12	0.01	0.36	0.37	0.21	0.36	0.34	0.22
KY523104.1_AUL78297.1_MF405918.2_QKU34226.1_BIFUNCTIONAL_DIHYDROFOLATE_REDUCTASE-THYMIDYLATESYNT	0.4	0.07	0.36	0	0.11	0.14	0.38	0.71	0.59	0.18	0.35
KY523104.1_AUL78298.1_MF405918.2_QKU34227.1_PUTATIVE_REPLICATION_FACTOR_C_SMALL_SUBUNIT_CDS	0.4	0.5	0.17	0.14	0	0.28	0.61	0.3	0.37	0.03	0.37
KY523104.1_AUL78299.1_MF405918.2_QKU34228.1_HYPOTHETICAL_PROTEIN_CDS	0.17	0.52	0.28	0.08	0	0.25	0.41	0.11	0.27	0.36	0.12
KY523104.1_AUL78300.1_MF405918.2_QKU34229.1_HYPOTHETICAL_PROTEIN_CDS	0.13	0.08	0.11	0	0.02	0.07	0.7	0.05	0.06	0.31	0.03
KY523104.1_AUL78300.1_MF405918.2_QKU34229.1_HYPOTHETICAL_PROTEIN_CDS	0.29	0.33	0.39	0.53	0	0.55	0.43	0.35	0.4	0.48	0.29
KY523104.1_AUL78299.1_MF405918.2_QKU34228.1_HYPOTHETICAL_PROTEIN_CDS	0.17	0.02	0.07	0	0	0.04	0.11	0.01	0.02	0.15	0.59
KY523104.1_AUL78301.1_MF405918.2_QKU34230.1_HYPOTHETICAL_PROTEIN_CDS	0.11	0.02	0.07	0	0	0.05	0.21	0.01	0.17	0.21	0.1
KY523104.1_AUL78302.1_MF405918.2_QKU34231.1_DNA-DIRECTED_RNA_POLYMERASE_SUBUNIT_1_CDS	0.61	0.49	0.03	0.01	0.01	0.03	0.83	0.02	0.45	0	0.78
KY523104.1_AUL78303.1_MF405918.2_QKU34232.1_HYPOTHETICAL_PROTEIN_CDS	0.65	0.14	0.21	0.19	0.4	0.17	0.56	0.26	0.39	0.41	0.53
KY523104.1_AUL78304.1_MF405918.2_AUL79600.2_HYPOTHETICAL_PROTEIN_CDS	0.32	0.34	0.32	0.18	0.16	0.33	0.19	0.11	0.25	0.4	0.43
KY523104.1_AUL78305.1_MF405918.2_QKU34233.1_HYPOTHETICAL_PROTEIN_CDS	0.39	0.56	0.23	0.05	0.03	0.3	0.58	0.38	0.4	0.13	0.38
KY523104.1_AUL78306.1_MF405918.2_AUL79603.2_HYPOTHETICAL_PROTEIN_CDS	0.39	0.62	0.44	0.22	0.31	0.42	0.37	0.42	0.47	0.27	0.36
KY523104.1_AUL78307.1_MF405918.2_QKU34234.1_HYPOTHETICAL_PROTEIN_CDS	0.28	0.27	0.38	0.17	0.28	0.53	0.43	0.44	0.46	0.5	0.27
KY523104.1_AUL78308.1_MF405918.2_QKU34235.1_MG368_PROTEIN_CDS	0.4	0.05	0.39	0	0.68	0.5	0.28	0.33	0.21	0.47	0.26
KY523104.1_AUL78309.1_MF405918.2_QKU34236.1_SWIB/MDM2_DOMAIN-CONTAINING_PROTEIN_CDS	0.42	0.74	0.4	0.23	0	0.21	0.09	0.05	0.43	0.07	0.15
KY523104.1_AUL78310.1_MF405918.2_QKU34237.1_PUTATIVE_FLAP_ENDONUCLEASE_1-LIKE_CDS	0.38	0.48	0.32	0.12	0	0.35	0.4	0.33	0.56	0.52	0.3
KY523104.1_AUL78311.1_MF405918.2_QKU34238.1_BIFUNCTIONAL_AAA_FAMILY_ATPASE_CHAPERONE_TRANSLOCAS	0.41	0.41	0.12	0	0.02	0.42	0.7	0.88	0.23	0.02	0.3
KY523104.1_AUL78312.1_MF405918.2_QKU34239.1_HYPOTHETICAL_PROTEIN_CDS	0.42	0.24	0.18	0.04	0	0.16	0.05	0.03	0.02	0.36	0.39
KY523104.1_AUL78313.1_MF405918.2_QKU34240.1_HYPOTHETICAL_PROTEIN_CDS	0.25	0.15	0.32	0.11	0.01	0.32	0.35	0.12	0.38	0.45	0.24
KY523104.1_AUL78314.1_MF405918.2_QKU34241.1_PUTATIVE_WD_REPEAT-CONTAINING_PROTEIN_CDS	0.81	0.43	0.27	0.32	0.43	0.25	0.45	0.04	0.46	0.52	0.59
KY523104.1_AUL78314.1_MF405918.2_QKU34241.1_PUTATIVE_WD_REPEAT-CONTAINING_PROTEIN_CDS	0.61	0.44	0.41	0.32	0.12	0.33	0.29	0.2	0.21	0.42	0.47
KY523104.1_AUL78315.1_MF405918.2_QKU34242.1_PUTATIVE_ORFAN_CDS	0.61	0.44	0.41	0.32	0.12	0.33	0.29	0.2	0.21	0.42	0.47
KY523104.1_AUL78316.1_MF405918.2_QKU34243.1_HYPOTHETICAL_PROTEIN_CDS	0.37	0.32	0.33	0.16	0.22	0.36	0.56	0.46	0.53	0.3	0.32
KY523104.1_AUL78317.1_MF405918.2_QKU34244.1_MG362_PROTEIN_CDS	0.4	0.36	0.3	0.12	0.22	0.36	0.57	0.38	0.39	0.27	0.47
KY523104.1_AUL78318.1_MF405918.2_QKU34245.1_HYPOTHETICAL_PROTEIN_CDS	0.22	0.2	0.34	0.08	0	0.48	0.39	0.2	0.47	0.46	0.09
KY523104.1_AUL78319.1_MF405918.2_QKU34246.1_HYPOTHETICAL_PROTEIN_CDS	0.38	0.47	0.37	0.29	0.46	0.33	0.24	0.25	0.51	0.52	0.34
KY523104.1_AUL78320.1_MF405918.2_QKU34247.1_PUTATIVE_ORFAN_CDS	0.16	0.09	0.09	0	0.02	0.05	0.31	0.02	0.09	0.29	0.2
KY523104.1_AUL78321.1_MF405918.2_QKU34248.1_HYPOTHETICAL_PROTEIN_CDS	0.21	0.44	0.44	0.12	0	0.21	0.05	0.02	0.13	0.39	0.21
KY523104.1_AUL78322.1_MF405918.2_QKU34249.1_HYPOTHETICAL_PROTEIN_CDS	0.35	0.4	0.3	0.21	0.03	0.33	0.2	0.1	0.5	0.45	0.4

KY523104.1_AUL78323.1_MF405918.2_QKU34250.1_HYPOTHETICAL_PROTEIN_CDS	0.26	0.16	0.27	0.13	0.47	0.64	0.09	0.28	0.49	0.49	0.16
KY523104.1_AUL78324.1_MF405918.2_QKU34251.1_DNAJ-LIKE_PROTEIN_CDS	0.38	0.33	0.28	0.14	0.25	0.6	0.43	0.22	0.53	0.55	0.38
KY523104.1_AUL78325.1_MF405918.2_QKU34252.1_ISOLEUCYL-TRNA_SYNTHETASE_CDS	0.44	0.61	0.31	0.19	0.38	0.46	0.47	0.73	0.56	0.2	0.45
KY523104.1_AUL78326.1_MF405918.2_QKU34253.1_PUTATIVE_ORFAN_CDS	0.43	0.49	0.34	0.35	0.19	0.36	0.37	0.16	0.32	0.38	0.45
KY523104.1_AUL78327.1_MF405918.2_QKU34254.1_VESICLE-FUSION_ATPASE_CDS	0.42	0.42	0.21	0.11	0.05	0.32	0.59	0.26	0.52	0.28	0.5
KY523104.1_AUL78327.1_MF405918.2_QKU34254.1_VESICLE-FUSION_ATPASE_CDS	0.24	0.19	0.29	0.12	0.11	0.38	0.43	0.06	0.11	0.44	0.32
KY523104.1_AUL78328.1_MF405918.2_QKU34255.1_HYPOTHETICAL_PROTEIN_CDS	0.24	0.19	0.29	0.12	0.11	0.38	0.43	0.06	0.11	0.44	0.32
KY523104.1_AUL78329.1_MF405918.2_QKU34256.1_PEPTIDASE_INHIBITOR_I9_CDS	0.4	0.67	0.47	0.17	0.03	0.45	0.49	0.5	0.47	0.22	0.43
KY523104.1_AUL78330.1_MF405918.2_QKU34257.1_PEPTIDASE_INHIBITOR_I9_CDS	0.39	0.65	0.42	0.08	0	0.45	0.52	0.47	0.5	0.2	0.3
KY523104.1_AUL78331.1_MF405918.2_QKU34258.1_PUTATIVE_ORFAN_CDS	0.78	0.27	0.3	0.35	0.02	0.31	0.03	0.07	0.47	0.42	0.5
KY523104.1_AUL78332.1_MF405918.2_QKU34259.1_TRNA_GUANYLYLTRANSFERASE_CDS	0.4	0.41	0.36	0.22	0.55	0.34	0.22	0.27	0.26	0.43	0.43
KY523104.1_AUL78333.1_MF405918.2_QKU34260.1_PUTATIVE_ORFAN_CDS	0.39	0.18	0.45	0.13	0.18	0.39	0.59	0.49	0.56	0.28	0.3
KY523104.1_AUL78333.1_MF405918.2_QKU34260.1_PUTATIVE_ORFAN_CDS	0.19	0.05	0.25	0.01	0.27	0.2	0.25	0.52	0.06	0.31	0.25
KY523104.1_AUL78334.1_MF405918.2_QKU34261.1_PUTATIVE_ORFAN_CDS	0.19	0.05	0.25	0.01	0.27	0.2	0.25	0.52	0.06	0.31	0.25
KY523104.1_AUL78335.1_MF405918.2_QKU34262.1_RAB_FAMILY_SMALL_GTPASE_CDS	0.4	0	0	0	0	0.07	0.9	0	0.03	0	0.01
KY523104.1_AUL78336.1_MF405918.2_QKU34263.1_MG356_PROTEIN_CDS	0.4	0.14	0.24	0.09	0.33	0.26	0.58	0.13	0.39	0.13	0.47
KY523104.1_AUL78337.1_MF405918.2_QKU34264.1_HYPOTHETICAL_PROTEIN_CDS	0.4	0.56	0.36	0.32	0.1	0.42	0.31	0.13	0.45	0.39	0.45
KY523104.1_AUL78338.1_MF405918.2_QKU34265.1_PUTATIVE_ORFAN_CDS	0.04	0.01	0.08	0	0	0.09	0.01	0	0.03	0.26	0.03
KY523104.1_AUL78339.1_MF405918.2_QKU34266.1_HYPOTHETICAL_PROTEIN_CDS	0.24	0.24	0.28	0.11	0.01	0.45	0.31	0.06	0.23	0.47	0.2
KY523104.1_AUL78340.1_MF405918.2_QKU34267.1_HYPOTHETICAL_PROTEIN_CDS	0.28	0.11	0.24	0.17	0.18	0.25	0.27	0.13	0.18	0.41	0.37
KY523104.1_AUL78341.1_MF405918.2_QKU34268.1_HEAT_SHOCK_PROTEIN_70-LIKE_PROTEIN_CDS	0.4	0.5	0.26	0.14	0.1	0.68	0.62	0.43	0.53	0.31	0.47
KY523104.1_AUL78342.1_MF405918.2_QKU34269.1_PUTATIVE_ORFAN_CDS	0.18	0.57	0.37	0.09	0.01	0.24	0.28	0.08	0.18	0.3	0.12
KY523104.1_AUL78343.1_MF405918.2_QKU34270.1_DUAL_SPECIFICITY_PHOSPHATASE_CDS	0.23	0.32	0.23	0.07	0.01	0.15	0.18	0.12	0.18	0.42	0.24
KY523104.1_AUL78344.1_MF405918.2_QKU34271.1_PUTATIVE_ORFAN_CDS	0.79	0.1	0.27	0.28	0	0.24	0	0.07	0.3	0.37	0.51
KY523104.1_AUL78345.1_MF405918.2_QKU34272.1_PUTATIVE_ORFAN_CDS	0.83	0.03	0.34	0.23	0	0.28	0.07	0.09	0.17	0.34	0.51
KY523104.1_AUL78346.1_MF405918.2_QKU34273.1_PUTATIVE_ORFAN_CDS	0.32	0.58	0.38	0.26	0.01	0.33	0.26	0.18	0.47	0.36	0.34
KY523104.1_AUL78347.1_MF405918.2_QKU34274.1_HYPOTHETICAL_PROTEIN_CDS	0.43	0.36	0.23	0.06	0	0.26	0.68	0.26	0.73	0.21	0.5
KY523104.1_AUL78348.1_MF405918.2_QKU34275.1_HYPOTHETICAL_PROTEIN_CDS	0.29	0.2	0.33	0.16	0.39	0.3	0.33	0.41	0.47	0.46	0.36
KY523104.1_AUL78349.1_MF405918.2_QKU34276.1_LON_PROTEASE-LIKE_PROTEIN_CDS	0.44	0.69	0.27	0.84	0.12	0.51	0.58	0.26	0.53	0.08	0.52
KY523104.1_AUL78350.1_MF405918.2_QKU34277.1_HYPOTHETICAL_PROTEIN_CDS	0.09	0.03	0.1	0	0.01	0.05	0.21	0.02	0.69	0.27	0.09
KY523104.1_AUL78351.1_MF405918.2_QKU34278.1_DNA_POLYMERASE_III_SUBUNIT_EPSILON_CDS	0.61	0	0.91	0.21	0.36	0.47	0.16	0.56	0.28	0.22	0.43
KY523104.1_AUL78352.1_MF405918.2_QKU34279.1_PUTATIVE_TRANSCRIPTION_INITIATION_FACTOR_IIB-LIKE_PROTEI	0.52	0.71	0.34	0.56	0.38	0.54	0.41	0.2	0.43	0.21	0.51
KY523104.1_AUL78353.1_MF405918.2_QKU34280.1_PUTATIVE_ORFAN_CDS	0.44	0.27	0.32	0.3	0.22	0.34	0.19	0.08	0.46	0.45	0.46
KY523104.1_AUL78354.1_MF405918.2_QKU34281.1_HYPOTHETICAL_PROTEIN_CDS	0.39	0.65	0.4	0.08	0.01	0.29	0.56	0.37	0.46	0.12	0.3
KY523104.1_AUL78355.1_MF405918.2_QKU34282.1_HYPOTHETICAL_PROTEIN_CDS	0.3	0.29	0.27	0.18	0.4	0.28	0.21	0.13	0.19	0.42	0.42
KY523104.1_AUL78356.1_MF405918.2_QKU34283.1_PUTATIVE_URACIL-DNA_GLYCOSYLASE_CDS	0.25	0.58	0.45	0.2	0.01	0.36	0.29	0.16	0.44	0.29	0.17
KY523104.1_AUL78357.1_MF405918.2_QKU34284.1_HYPOTHETICAL_PROTEIN_CDS	0.37	0.44	0.46	0.57	0.12	0.39	0.1	0.58	0.43	0.19	0.38
KY523104.1_AUL78358.1_MF405918.2_QKU34285.1_PUTATIVE_ORFAN_CDS	0.34	0.37	0.38	0.29	0.47	0.35	0.33	0.3	0.37	0.46	0.4
KY523104.1_AUL78359.1_MF405918.2_QKU34286.1_HYPOTHETICAL_PROTEIN_CDS	0.4	0.52	0.35	0.17	0.07	0.36	0.41	0.27	0.28	0.36	0.43
KY523104.1_AUL78360.1_MF405918.2_QKU34287.1_PUTATIVE_ORFAN_CDS	0.4	0.71	0.58	0.43	0.74	0.28	0.09	0.31	0.36	0.46	0.31
KY523104.1_AUL78361.1_MF405918.2_QKU34288.1_INTEIN-CONTAINING_DNA-DIRECTED_RNA_POLYMERASE_SUBUNI	0.33	0.39	0.34	0.18	0.31	0.33	0.49	0.3	0.45	0.36	0.4
KY523104.1_AUL78362.1_MF405918.2_QKU34289.1_INTEIN-CONTAINING_DNA-DIRECTED_RNA_POLYMERASE_SUBUNI	0.54	0.29	0.28	0.26	0.07	0.37	0.43	0.12	0.45	0.43	0.48

KY523104.1_AUL78363.1_MF405918.2_QKU34290.1_PUTATIVE_HNH_ENDONUCLEASE_CDS	0.26	0.38	0.3	0.14	0.02	0.27	0.3	0.31	0.31	0.4	0.32
KY523104.1_AUL78364.1_MF405918.2_QKU34291.1_PUTATIVE_ORFAN_CDS	0.32	0.02	0.17	0.03	0.01	0.13	0.12	0.02	0.13	0.4	0.42
KY523104.1_AUL78365.1_MF405918.2_QKU34292.1_PUTATIVE_ORFAN_CDS	0.55	0.34	0.33	0.22	0.46	0.32	0.13	0.14	0.38	0.44	0.45
KY523104.1_AUL78366.1_MF405918.2_QKU34293.1_HYPOTHETICAL_PROTEIN_CDS	0.3	0.07	0.2	0.06	0.05	0.16	0.27	0.06	0.09	0.4	0.42
KY523104.1_AUL78367.1_MF405918.2_QKU34294.1_HYPOTHETICAL_PROTEIN_CDS	0.4	0.78	0.4	0.47	0	0.29	0.02	0.13	0.49	0.25	0.26
KY523104.1_AUL78368.1_MF405918.2_QKU34295.1_DNA-DIRECTED_RNA_POLYMERASE_SUBUNIT_6_CDS	0.32	0.53	0.41	0.52	0.04	0.39	0.33	0.37	0.39	0.34	0.37
KY523104.1_AUL78369.1_MF405918.2_QKU34296.1_HYPOTHETICAL_PROTEIN_CDS	0.22	0.48	0.42	0.52	0.01	0.3	0.27	0.28	0.24	0.33	0.2
KY523104.1_AUL78370.1_MF405918.2_QKU34297.1_PUTATIVE_ORFAN_CDS	0.22	0.02	0.73	0.02	1	0.33	0.01	0.18	0.14	0.56	0.06
KY523104.1_AUL78371.1_MF405918.2_QKU34298.1_HYPOTHETICAL_PROTEIN_CDS	0.39	0.63	0.42	0.23	0.03	0.38	0.3	0.36	0.41	0.24	0.4
KY523104.1_AUL78372.1_MF405918.2_QKU34299.1_PUTATIVE_ORFAN_CDS	0.02	0.01	0.13	0.01	0	0.66	0.01	0.01	0.08	0.19	0.01
KY523104.1_AUL78373.1_MF405918.2_QKU34300.1_MG247_PROTEIN_CDS	0.26	0.23	0.19	0.09	0.01	0.23	0.2	0.06	0.07	0.4	0.34
KY523104.1_AUL78374.1_MF405918.2_QKU34301.1_PUTATIVE_ORFAN_CDS	0.2	0.28	0.22	0.04	0.02	0.17	0.45	0.36	0.03	0.35	0.23
KY523104.1_AUL78375.1_MF405918.2_QKU34302.1_CHEMOTAXIS_PROTEIN_CDS	0.17	0.41	0.29	0.05	0	0.19	0.13	0.18	0.08	0.32	0.24
KY523104.1_AUL78376.1_MF405918.2_QKU34303.1_ZN-DEPENDENT_PEPTIDASE_CDS	0.38	0.46	0.38	0.25	0.04	0.47	0.22	0.09	0.47	0.41	0.43
KY523104.1_AUL78377.1_MF405918.2_QKU34304.1_PUTATIVE_SERINE/THREONINE-PROTEIN_KINASE_CDS	0.39	0.02	0.34	0.01	0	0.36	0.58	0.28	0.42	0.25	0.33
KY523104.1_AUL78378.1_MF405918.2_QKU34305.1_PUTATIVE_NEITHER_INACTIVATION_NOR_AFTERPOTENTIALPROTE	0.4	0.49	0.3	0.29	0	0.58	0.59	0.26	0.47	0.22	0.44
KY523104.1_AUL78379.1_MF405918.2_QKU34306.1_HYPOTHETICAL_PROTEIN_CDS	0.2	0.07	0.23	0	0	0.3	0.58	0.09	0.08	0.28	0.04
KY523104.1_AUL78380.1_MF405918.2_QKU34307.1_PUTATIVE_PROCOLLAGEN-LYSINE_CDS	0.51	0.68	0.27	0.27	0.49	0.63	0.48	0.17	0.46	0.27	0.5
KY523104.1_AUL78381.1_MF405918.2_QKU34308.1_PUTATIVE_ORFAN_CDS	0.39	0.15	0.33	0.02	0.21	0.31	0.59	0.29	0.37	0.15	0.22
KY523104.1_AUL78382.1_MF405918.2_QKU34309.1_AMINO_OXIDASE_FAMILY_PROTEIN_CDS	0.36	0.34	0.28	0.22	0.26	0.3	0.53	0.22	0.5	0.32	0.4
KY523104.1_AUL78383.1_MF405918.2_QKU34310.1_PUTATIVE_DNA_TOPOISOMERASE_2_ISOFORM_X2_CDS	0.56	0.22	0.26	0.15	0.71	0.45	0.36	0.33	0.46	0.73	0.46
KY523104.1_AUL78384.1_MF405918.2_QKU34311.1_PUTATIVE_ATP-CITRATE_SYNTHASE_ISOFORM_X3_CDS	0.43	0.68	0.22	0.53	0.09	0.73	0.5	0.23	0.58	0.56	0.48
KY523104.1_AUL78385.1_MF405918.2_QKU34312.1_HYPOTHETICAL_PROTEIN_CDS	0.41	0.66	0.4	0.27	0.17	0.41	0.29	0.29	0.44	0.25	0.46
KY523104.1_AUL78386.1_MF405918.2_QKU34313.1_PUTATIVE_ORFAN_CDS	0.56	0.46	0.38	0.28	0.06	0.37	0.29	0.08	0.41	0.43	0.47
KY523104.1_AUL78387.1_MF405918.2_QKU34314.1_TOPOISOMERASE_I_CDS	0.4	0.61	0.34	0.13	0.3	0.51	0.55	0.77	0.52	0.09	0.38
KY523104.1_AUL78388.1_MF405918.2_QKU34315.1_HYPOTHETICAL_PROTEIN_CDS	0.23	0.3	0.23	0.07	0	0.36	0.16	0.04	0.16	0.42	0.23
KY523104.1_AUL78389.1_MF405918.2_QKU34316.1_HYPOTHETICAL_PROTEIN_CDS	0.49	0.68	0.42	0.5	0.06	0.39	0.18	0.25	0.45	0.26	0.48
KY523104.1_AUL78390.1_MF405918.2_QKU34317.1_PUTATIVE_ORFAN_CDS	0.21	0.08	0.11	0.01	0.07	0.06	0.31	0.03	0.11	0.31	0.2
KY523104.1_AUL78391.1_MF405918.2_QKU34318.1_F10-LIKE_KINASE_CDS	0.28	0.07	0.25	0.02	0.58	0.22	0.09	0.1	0.11	0.4	0.28
KY523104.1_AUL78392.1_MF405918.2_QKU34319.1_HYPOTHETICAL_PROTEIN_CDS	0.27	0.4	0.43	0.52	0.04	0.36	0.44	0.22	0.28	0.38	0.27
KY523104.1_AUL78393.1_MF405918.2_QKU34320.1_HYPOTHETICAL_PROTEIN_CDS	0.4	0.34	0.09	0	0	0.23	0.62	0.17	0.35	0.04	0.28
KY523104.1_AUL78394.1_MF405918.2_AUL79690.2_REPEAT_PROTEIN_CDS	0.39	0.52	0.3	0.04	0.01	0.19	0.47	0.36	0.58	0.5	0.23
KY523104.1_AUL78395.1_MF405918.2_QKU34321.1_HYPOTHETICAL_PROTEIN_CDS	0.32	0.49	0.39	0.2	0.05	0.43	0.41	0.18	0.39	0.36	0.41
KY523104.1_AUL78396.1_MF405918.2_QKU34322.1_PUTATIVE_ORFAN_CDS	0.4	0.22	0.32	0.11	0.58	0.52	0.5	0.4	0.37	0.58	0.38
KY523104.1_AUL78397.1_MF405918.2_QKU34323.1_PUTATIVE_ORFAN_CDS	0.29	0.57	0.44	0.24	0.25	0.33	0.24	0.21	0.46	0.36	0.2
KY523104.1_AUL78398.1_MF405918.2_QKU34324.1_HYPOTHETICAL_PROTEIN_CDS	0.41	0.47	0.32	0.83	0.26	0.41	0.41	0.13	0.45	0.35	0.48
KY523104.1_AUL78399.1_MF405918.2_QKU34325.1_REPEAT_PROTEIN_CDS	0.28	0.33	0.34	0.12	0	0.53	0.42	0.27	0.27	0.45	0.4
KY523104.1_AUL78400.1_MF405918.2_QKU34326.1_PUTATIVE_FIBRIL_ASSOCIATED_PROTEIN_CDS	0.57	0.37	0.38	0.3	0.27	0.38	0.27	0.1	0.44	0.45	0.47
KY523104.1_AUL78401.1_MF405918.2_QKU34327.1_PUTATIVE_ORFAN_CDS	0.43	0.65	0.42	0.24	0.48	0.43	0.22	0.38	0.4	0.28	0.44
KY523104.1_AUL78402.1_MF405918.2_QKU34328.1_RAB_FAMILY_GTPASE_CDS	0.4	0	0.04	0	0	0.2	0.79	0	0.1	0	0.05
KY523104.1_AUL78403.1_MF405918.2_QKU34329.1_PUTATIVE_MAJOR_CAPSID_PROTEIN_CDS	0.26	0.05	0.38	0.12	0.09	0.43	0.55	0.12	0.36	0.48	0.23
KY523104.1_AUL78404.1_MF405918.2_QKU34330.1_PUTATIVE_ORFAN_CDS	0.4	0.76	0.22	0.12	0.03	0.05	0.26	0.01	0.39	0	0.04

KY523104.1_AUL78405.1_MF405918.2_QKU34331.1_PUTATIVE_NEUROCAN_CORE_PROTEIN_CDS	0.74	0.39	0.35	0.25	0.51	0.4	0.18	0.22	0.35	0.41	0.46
KY523104.1_AUL78406.1_MF405918.2_QKU34332.1_UDP-GLUCOSE_4-EPIMERASE_GALE_CDS	0.34	0.19	0.97	0.25	0.43	0.43	0.48	0.26	0.17	0.43	0.32
KY523104.1_AUL78407.1_MF405918.2_QKU34333.1_PUTATIVE_ORFAN_CDS	0.1	0.06	0.07	0	0	0.04	0.34	0.01	0.07	0.17	0.17
KY523104.1_AUL78408.1_MF405918.2_QKU34334.1_PUTATIVE_BRO-N_DOMAIN-CONTAINING_PROTEIN_CDS	0.31	0.61	0.44	0.16	0	0.39	0.32	0.44	0.43	0.23	0.25
KY523104.1_AUL78409.1_MF405918.2_QKU34335.1_PUTATIVE_ORFAN_CDS	0.2	0.06	0.28	0.03	0.44	0.44	0.14	0.05	0.07	0.4	0.16
KY523104.1_AUL78410.1_MF405918.2_QKU34336.1_HYPOTHETICAL_PROTEIN_CDS	0.4	0.25	0.27	0.16	0.29	0.75	0.54	0.4	0.43	0.45	0.45
KY523104.1_AUL78411.1_MF405918.2_QKU34337.1_T5ORF172_DOMAIN-CONTAINING_PROTEIN_CDS	0.66	0.14	0.37	0.39	0.39	0.3	0.24	0.1	0.45	0.45	0.48
KY523104.1_AUL78412.1_MF405918.2_QKU34338.1_MG211_PROTEIN_CDS	0.6	0.17	0.2	0.08	0.01	0.17	0.02	0.01	0.05	0.38	0.45
KY523104.1_AUL78413.1_MF405918.2_QKU34339.1_HYPOTHETICAL_PROTEIN_CDS	0.45	0.63	0.4	0.22	0.11	0.4	0.27	0.17	0.43	0.35	0.46
KY523104.1_AUL78414.1_MF405918.2_AUL79706.2_HYPOTHETICAL_PROTEIN_CDS	0.26	0.11	0.2	0.06	0.01	0.25	0.29	0.03	0.07	0.4	0.32
KY523104.1_AUL78415.1_MF405918.2_QKU34340.1_HYPOTHETICAL_PROTEIN_CDS	0.28	0.17	0.2	0.07	0.05	0.21	0.16	0.07	0.11	0.36	0.42
KY523104.1_AUL78416.1_MF405918.2_QKU34341.1_PUTATIVE_ORFAN_CDS	0	0	0.03	0	0	0.02	0	0	0	0.12	0
KY523104.1_AUL78417.1_MF405918.2_QKU34342.1_ESTA_FAMILY_SERINE_HYDROLASE_CDS	0.26	0.43	0.39	0.1	0.01	0.51	0.39	0.47	0.41	0.43	0.24
KY523104.1_AUL78418.1_MF405918.2_QKU34343.1_ENDOTYPE_6-AMINOHEXANOAT-OLIGOMER_HYDROLASE_CDS	0.41	0.28	0.35	0.2	0.41	0.51	0.31	0.34	0.33	0.44	0.43
KY523104.1_AUL78419.1_MF405918.2_QKU34344.1_HYPOTHETICAL_PROTEIN_CDS	0.38	0.19	0.36	0.06	0.59	0.35	0.27	0.4	0.35	0.52	0.4
KY523104.1_AUL78420.1_MF405918.2_QKU34345.1_PUTATIVE_ORFAN_CDS	0.26	0.3	0.2	0.13	0.02	0.25	0.15	0.03	0.18	0.38	0.36
KY523104.1_AUL78421.1_MF405918.2_AUL79713.2_PUTATIVE_ORFAN_CDS	0.24	0.14	0.15	0.02	0.21	0.1	0.21	0.05	0.06	0.28	0.43
KY523104.1_AUL78422.1_MF405918.2_AUL79714.2_HYPOTHETICAL_PROTEIN_CDS	0.4	0.18	0.23	0.02	0.11	0.41	0.6	0.22	0.39	0.15	0.28
KY523104.1_AUL78423.1_MF405918.2_AUL79715.2_PUTATIVE_ORFAN_CDS	0.26	0.47	0.44	0.17	0.64	0.33	0.26	0.26	0.25	0.46	0.23
KY523104.1_AUL78424.1_MF405918.2_AUL79716.2_PHOSPHATASES_II_CDS	0.3	0.07	0.17	0.01	0.03	0.11	0.14	0.05	0.11	0.3	0.53
KY523104.1_AUL78425.1_MF405918.2_QKU34346.1_MG15_PROTEIN_CDS	0.89	0.44	0.37	0.27	0.32	0.44	0.25	0.05	0.46	0.42	0.57
KY523104.1_AUL78426.1_MF405918.2_QKU34347.1_HYPOTHETICAL_PROTEIN_CDS	0.5	0.18	0.33	0.11	0.73	0.37	0.32	0.33	0.37	0.63	0.44
KY523104.1_AUL78427.1_MF405918.2_QKU34348.1_HYPOTHETICAL_PROTEIN_CDS	0.26	0.13	0.2	0.06	0.01	0.27	0.15	0.02	0.22	0.44	0.31
KY523104.1_AUL78428.1_MF405918.2_QKU34349.1_HYPOTHETICAL_PROTEIN_CDS	0.72	0.05	0.28	0.23	0	0.29	0.24	0.14	0.19	0.34	0.5
KY523104.1_AUL78429.1_MF405918.2_QKU34350.1_HYPOTHETICAL_PROTEIN_CDS	0.39	0.35	0.29	0.03	0.01	0.28	0.59	0.5	0.44	0.15	0.31
KY523104.1_AUL78430.1_MF405918.2_QKU34351.1_PUTATIVE_ORFAN_CDS	0.12	0.07	0.04	0	0	0.03	0.22	0	0	0.18	0.1
KY523104.1_AUL78431.1_MF405918.2_QKU34352.1_HYPOTHETICAL_PROTEIN_CDS	0.34	0.53	0.26	0.07	0.01	0.21	0.57	0.34	0.44	0.14	0.33
KY523104.1_AUL78432.1_MF405918.2_QKU34353.1_HYPOTHETICAL_PROTEIN_CDS	0.4	0.13	0.28	0.15	0.26	0.29	0.57	0.43	0.44	0.33	0.47
KY523104.1_AUL78432.1_MF405918.2_QKU34353.1_HYPOTHETICAL_PROTEIN_CDS	0.27	0.05	0.34	0	0.51	0.31	0.04	0.22	0.11	0.45	0.11
KY523104.1_AUL78433.1_MF405918.2_QKU34354.1_PUTATIVE_ORFAN_CDS	0.27	0.05	0.34	0	0.51	0.31	0.04	0.22	0.11	0.45	0.11
KY523104.1_AUL78434.1_MF405918.2_QKU34355.1_HYPOTHETICAL_PROTEIN_CDS	0.18	0.58	0.41	0.13	0	0.34	0.34	0.14	0.39	0.29	0.11
KY523104.1_AUL78435.1_MF405918.2_QKU34356.1_PUTATIVE_ORFAN_CDS	0.29	0.11	0.16	0.03	0.03	0.11	0.13	0.04	0.17	0.35	0.44
KY523104.1_AUL78436.1_MF405918.2_QKU34357.1_PUTATIVE_ORFAN_CDS	0.37	0.27	0.3	0.19	0.46	0.31	0.53	0.38	0.44	0.37	0.38
KY523104.1_AUL78437.1_MF405918.2_QKU34358.1_HYPOTHETICAL_PROTEIN_CDS	0.49	0.45	0.36	0.22	0.45	0.37	0.15	0.14	0.4	0.39	0.45
KY523104.1_AUL78438.1_MF405918.2_QKU34359.1_PUTATIVE_ORFAN_CDS	0.66	0.35	0.34	0.33	0.03	0.35	0.2	0.05	0.46	0.41	0.49
KY523104.1_AUL78439.1_MF405918.2_QKU34360.1_ANKYRIN_REPEAT_PROTEIN_CDS	0.46	0.55	0.31	0.32	0.02	0.36	0.3	0.23	0.5	0.37	0.48
KY523104.1_AUL78440.1_MF405918.2_QKU34361.1_PUTATIVE_ORFAN_CDS	0.23	0.2	0.21	0.05	0.33	0.4	0.18	0.05	0.2	0.44	0.12
KY523104.1_AUL78441.1_MF405918.2_QKU34362.1_PUTATIVE_ORFAN_CDS	0.33	0.57	0.32	0.17	0	0.35	0.14	0.14	0.53	0.6	0.13
KY523104.1_AUL78442.1_MF405918.2_QKU34363.1_PUTATIVE_ORFAN_CDS	0.74	0.53	0.19	0.13	0.24	0.24	0.53	0.01	0.42	0.46	0.55
KY523104.1_AUL78443.1_MF405918.2_QKU34364.1_HYPOTHETICAL_PROTEIN_CDS	0.12	0.16	0.12	0.03	0.01	0.07	0.19	0.03	0.11	0.29	0.19
KY523104.1_AUL78444.1_MF405918.2_QKU34365.1_HYPOTHETICAL_PROTEIN_CDS	0.27	0.21	0.27	0.11	0	0.51	0.29	0.03	0.12	0.43	0.4
KY523104.1_AUL78445.1_MF405918.2_QKU34366.1_HYPOTHETICAL_PROTEIN_CDS	0.09	0.03	0.11	0	0	0.07	0.2	0.02	0.27	0.22	0.15

KY523104.1_AUL78446.1_MF405918.2_QKU34367.1_HYPOTHETICAL_PROTEIN_CDS	0.18	0.24	0.23	0.04	0	0.49	0.3	0.03	0.1	0.44	0.11
KY523104.1_AUL78447.1_MF405918.2_QKU34368.1_HYPOTHETICAL_PROTEIN_CDS	0.17	0.05	0.16	0.02	0	0.5	0.07	0.03	0.12	0.27	0.16
KY523104.1_AUL78448.1_MF405918.2_QKU34369.1_PUTATIVE_ORFAN_CDS	0.54	0.28	0.36	0.12	0.01	0.47	0.11	0.12	0.1	0.37	0.43
KY523104.1_AUL78449.1_MF405918.2_QKU34370.1_PUTATIVE_ORFAN_CDS	0.04	0.03	0.02	0	0	0.02	0.2	0	0.01	0.14	0.15
KY523104.1_AUL78450.1_MF405918.2_QKU34371.1_PUTATIVE_ORFAN_CDS	0.65	0.19	0.23	0.14	0.04	0.21	0.04	0.04	0.05	0.39	0.45
KY523104.1_AUL78449.1_MF405918.2_QKU34370.1_PUTATIVE_ORFAN_CDS	0.06	0.09	0.01	0	0	0.01	0.05	0	0	0.18	0.32
KY523104.1_AUL78451.1_MF405918.2_QKU34372.1_PUTATIVE_PHAGE_STRUCTURAL_PROTEIN_CDS	0.19	0.01	0.34	0.64	0	0.58	0.49	0.13	0.25	0.47	0.06
KY523104.1_AUL78452.1_MF405918.2_QKU34373.1_PUTATIVE_ORFAN_CDS	0.16	0.01	0.08	0	0	0.06	0.05	0.01	0.01	0.2	0.14
KY523104.1_AUL78453.1_MF405918.2_QKU34374.1_PUTATIVE_ORFAN_CDS	0.03	0.02	0.07	0	0	0.04	0.01	0.03	0.01	0.21	0.09
KY523104.1_AUL78454.1_MF405918.2_QKU34375.1_PUTATIVE_ORFAN_CDS	0.28	0.37	0.36	0.25	0	0.24	0.54	0.25	0.43	0.26	0.34
KY523104.1_AUL78455.1_MF405918.2_QKU34376.1_PUTATIVE_ORFAN_CDS	0.21	0.1	0.14	0.02	0.04	0.08	0.26	0.03	0.5	0.33	0.26
KY523104.1_AUL78456.1_MF405918.2_QKU34377.1_PUTATIVE_ORFAN_CDS	0.29	0.14	0.21	0.58	0.01	0.18	0.12	0.05	0.1	0.35	0.3
KY523104.1_AUL78457.1_MF405918.2_QKU34378.1_PUTATIVE_ORFAN_CDS	0.15	0.11	0.15	0	0.02	0.1	0.6	0.06	0.06	0.3	0.04
KY523104.1_AUL78458.1_MF405918.2_QKU34379.1_PUTATIVE_ORFAN_CDS	0.27	0.57	0.43	0.31	0	0.32	0.2	0.15	0.45	0.33	0.25
KY523104.1_AUL78459.1_MF405918.2_QKU34380.1_FAMILY_25_PROTEIN_CDS	0.25	0.23	0.2	0.05	0.45	0.18	0.13	0.11	0.12	0.4	0.22
KY523104.1_AUL78460.1_MF405918.2_QKU34381.1_HYPOTHETICAL_PROTEIN_CDS	0.2	0.36	0.25	0.07	0	0.19	0.2	0.01	0.04	0.37	0.26
KY523104.1_AUL78461.1_MF405918.2_QKU34382.1_PUTATIVE_ORFAN_CDS	0.12	0.09	0.09	0	0.01	0.04	0.28	0.02	0.76	0.26	0.1
KY523104.1_AUL78462.1_MF405918.2_AUL79791.1_MG15_PROTEIN_CDS	0.67	0.42	0.42	0.36	0.36	0.38	0.23	0.24	0.45	0.43	0.49
KY523104.1_AUL78463.1_MF405918.2_QKU34383.1_PUTATIVE_ORFAN_CDS	0.63	0.21	0.33	0.24	0.24	0.26	0.16	0.17	0.2	0.4	0.46
KY523104.1_AUL78464.1_MF405918.2_QKU34384.1_HYPOTHETICAL_PROTEIN_CDS	0.15	0.15	0.32	0	0.17	0.15	0.18	0.55	0.22	0.25	0.22
KY523104.1_AUL78465.1_MF405918.2_QKU34385.1_PUTATIVE_ORFAN_CDS	0.16	0.14	0.16	0	0	0.15	0.6	0.07	0.02	0.28	0.03
KY523104.1_AUL78466.1_MF405918.2_AUL79745.2_HYPOTHETICAL_PROTEIN_CDS	0.3	0.42	0.34	0.15	0.41	0.27	0.21	0.27	0.51	0.5	0.17
KY523104.1_AUL78467.1_MF405918.2_QKU34386.1_HYPOTHETICAL_PROTEIN_CDS	0.32	0.59	0.42	0.12	0.01	0.4	0.44	0.25	0.42	0.27	0.31
KY523104.1_AUL78468.1_MF405918.2_QKU34387.1_HYPOTHETICAL_PROTEIN_CDS	0.4	0.65	0.46	0.07	0	0.53	0.54	0.37	0.48	0.12	0.32
KY523104.1_AUL78468.1_MF405918.2_QKU34387.1_HYPOTHETICAL_PROTEIN_CDS	0.24	0.1	0.19	0.21	0.05	0.12	0.22	0.37	0.12	0.3	0.32
KY523104.1_AUL78469.1_MF405918.2_QKU34388.1_PUTATIVE_ORFAN_CDS	0.24	0.1	0.19	0.21	0.05	0.12	0.22	0.37	0.12	0.3	0.32
KY523104.1_AUL78470.1_MF405918.2_QKU34389.1_COLLAGEN_ALPHA-1(XXVII)_CHAIN_FLAGS_PRECURSOR_CDS	0.4	0.47	0.27	0.23	0.59	0.39	0.57	0.34	0.66	0.72	0.36
KY523104.1_AUL78471.1_MF405918.2_QKU34390.1_HYPOTHETICAL_PROTEIN_CDS	0.76	0.7	0.46	0.29	0.34	0.45	0.17	0.16	0.46	0.46	0.53
KY523104.1_AUL78472.1_MF405918.2_QKU34391.1_PUTATIVE_ORFAN_CDS	0.19	0.14	0.15	0.01	0.04	0.07	0.4	0.07	0.08	0.32	0.12
KY523104.1_AUL78473.1_MF405918.2_QKU34392.1_PUTATIVE_ORFAN_CDS	0.36	0.35	0.37	0.15	0.56	0.31	0.13	0.31	0.46	0.6	0.33
KY523104.1_AUL78474.1_MF405918.2_QKU34393.1_PUTATIVE_ORFAN_CDS	0.21	0.48	0.38	0.13	0.05	0.29	0.26	0.16	0.25	0.35	0.22
KY523104.1_AUL78475.1_MF405918.2_QKU34394.1_HYPOTHETICAL_PROTEIN_CDS	0.33	0.12	0.33	0.01	0	0.68	0.56	0.6	0.32	0.33	0.13
KY523104.1_AUL78476.1_MF405918.2_QKU34395.1_HYPOTHETICAL_PROTEIN_CDS	0.52	0.29	0.35	0.26	0.02	0.55	0.23	0.08	0.47	0.36	0.47
KY523104.1_AUL78477.1_MF405918.2_QKU34396.1_PUTATIVE_ORFAN_CDS	0.51	0.21	0.39	0.54	0.45	0.35	0.1	0.33	0.19	0.42	0.45
KY523104.1_AUL78478.1_MF405918.2_QKU34397.1_PUTATIVE_ORFAN_CDS	0	0	0.03	0	0	0.03	0	0	0	0.14	0
KY523104.1_AUL78479.1_MF405918.2_QKU34398.1_HYPOTHETICAL_PROTEIN_CDS	0.24	0.18	0.23	0.06	0.4	0.25	0.18	0.39	0.03	0.4	0.28
KY523104.1_AUL78480.1_MF405918.2_QKU34399.1_HYPOTHETICAL_PROTEIN_CDS	0.28	0.33	0.22	0.01	0	0.25	0.6	0.18	0.34	0.19	0.19
KY523104.1_AUL78481.1_MF405918.2_QKU34400.1_HYPOTHETICAL_PROTEIN_CDS	0.4	0.43	0.27	0.07	0.29	0.25	0.59	0.37	0.4	0.11	0.42
KY523104.1_AUL78482.1_MF405918.2_QKU34401.1_PUTATIVE_E3_UBIQUITIN-PROTEIN_LIGASE_XBAT35-LIKE_CDS	0.96	0.04	0.22	0.29	0	0.31	0.15	0.06	0.44	0.29	0.71
KY523104.1_AUL78483.1_MF405918.2_QKU34402.1_PUTATIVE_ORFAN_CDS	0.48	0.33	0.37	0.29	0.6	0.34	0.26	0.44	0.35	0.56	0.44
KY523104.1_AUL78484.1_MF405918.2_QKU34403.1_PUTATIVE_ORFAN_CDS	0.8	0.29	0.39	0.33	0.65	0.43	0.26	0.25	0.36	0.54	0.5
KY523104.1_AUL78485.1_MF405918.2_QKU34404.1_PUTATIVE_ORFAN_CDS	0.47	0.39	0.39	0.14	0.63	0.31	0.13	0.37	0.22	0.54	0.43

KY523104.1_AUL78486.1_MF405918.2_QKU34405.1_PUTATIVE_SNF1-RELATED_PROTEIN_KINASE_CATALYTIC_SUBUNIT	0.33	0.32	0.36	0.27	0.22	0.32	0.55	0.47	0.44	0.33	0.31
KY523104.1_AUL78487.1_MF405918.2_QKU34406.1_HYPOTHETICAL_PROTEIN_CDS	0.33	0.33	0.32	0.17	0.1	0.34	0.56	0.26	0.43	0.23	0.41
KY523104.1_AUL78488.1_MF405918.2_QKU34407.1_PUTATIVE_ORFAN_CDS	0.09	0.06	0.05	0	0.08	0.04	0.18	0.03	0.03	0.29	0.24
KY523104.1_AUL78488.1_MF405918.2_QKU34407.1_PUTATIVE_ORFAN_CDS	0.25	0.03	0.31	0.48	0.03	0.39	0.46	0.08	0.2	0.41	0.38
KY523104.1_AUL78487.1_MF405918.2_QKU34406.1_HYPOTHETICAL_PROTEIN_CDS	0.06	0.01	0.09	0	0	0.15	0.05	0.02	0.02	0.14	0.49
KY523104.1_AUL78489.1_MF405918.2_QKU34408.1_HYPOTHETICAL_PROTEIN_CDS	0.61	0.42	0.31	0.24	0.06	0.33	0.41	0.1	0.35	0.41	0.47
KY523104.1_AUL78490.1_MF405918.2_QKU34409.1_PUTATIVE_ORFAN_CDS	0.21	0.2	0.22	0.04	0	0.49	0.26	0.1	0.07	0.37	0.16
KY523104.1_AUL78491.1_MF405918.2_QKU34410.1_PUTATIVE_ORFAN_CDS	0.33	0.64	0.4	0.2	0	0.27	0.18	0.13	0.43	0.22	0.16
KY523104.1_AUL78492.1_MF405918.2_QKU34411.1_HYPOTHETICAL_PROTEIN_CDS	0.06	0.07	0.1	0	0.01	0.06	0.25	0.02	0.09	0.21	0.06
KY523104.1_AUL78493.1_MF405918.2_QKU34412.1_PUTATIVE_ORFAN_CDS	0.18	0.05	0.09	0	0.01	0.07	0.23	0.02	0.03	0.24	0.34
KY523104.1_AUL78494.1_MF405918.2_QKU34413.1_PUTATIVE_ORFAN_CDS	0.58	0.05	0.21	0.12	0.01	0.19	0.11	0.07	0.15	0.36	0.46
KY523104.1_AUL78495.1_MF405918.2_QKU34414.1_PUTATIVE_ORFAN_CDS	0.12	0.13	0.12	0	0.01	0.06	0.14	0.01	0.05	0.26	0.16
KY523104.1_AUL78496.1_MF405918.2_AUL79777.2_PUTATIVE_ORFAN_CDS	0.61	0.27	0.24	0.18	0.09	0.48	0.5	0.05	0.42	0.45	0.49
KY523104.1_AUL78497.1_MF405918.2_QKU34415.1_PUTATIVE_ORFAN_CDS	0.3	0.14	0.16	0.02	0.02	0.09	0.16	0.03	0.04	0.28	0.51
KY523104.1_AUL78498.1_MF405918.2_QKU34416.1_PUTATIVE_ORFAN_CDS	0.34	0.23	0.42	0.16	0.54	0.33	0.29	0.4	0.51	0.52	0.28
KY523104.1_AUL78499.1_MF405918.2_QKU34417.1_PUTATIVE_ORFAN_CDS	0.4	0.14	0.39	0.17	0.55	0.35	0.15	0.54	0.24	0.34	0.43
KY523104.1_AUL78500.1_MF405918.2_QKU34418.1_HYPOTHETICAL_PROTEIN_CDS	0.2	0.27	0.29	0.1	0.33	0.23	0.37	0.1	0.28	0.4	0.14
KY523104.1_AUL78501.1_MF405918.2_QKU34419.1_PUTATIVE_VIRION-ASSOCIATED_MEMBRANE_PROTEIN_CDS	0.4	0.41	0.42	0.26	0.06	0.32	0.5	0.3	0.41	0.39	0.46
KY523104.1_AUL78502.1_MF405918.2_QKU34420.1_PUTATIVE_ORFAN_CDS	0.79	0.06	0.05	0.48	0.13	0.09	0.24	0	0.56	0.42	0.72
KY523104.1_AUL78503.1_MF405918.2_QKU34421.1_CU-ZN_SUPEROXIDE_DISMUTASE_CDS	0.4	0.76	0.3	0.98	0.24	0.15	0.56	0.72	0.39	0	0.34
KY523104.1_AUL78504.1_MF405918.2_QKU34422.1_PUTATIVE_ORFAN_CDS	0.27	0.57	0.42	0.15	0.36	0.45	0.14	0.2	0.42	0.33	0.24
KY523104.1_AUL78505.1_MF405918.2_AUL79784.2_HYPOTHETICAL_PROTEIN_CDS	0.27	0.16	0.27	0.07	0.38	0.27	0.18	0.31	0.33	0.4	0.29
KY523104.1_AUL78506.1_MF405918.2_QKU34423.1_DNA_POLYMERASE_FAMILY_X_CDS	0.14	0.08	0.08	0	0.07	0.05	0.26	0.02	0.04	0.25	0.13
KY523104.1_AUL78507.1_MF405918.2_QKU34424.1_PUTATIVE_DNA_POLYMERASE_FAMILY_X_CDS	0.11	0.07	0.05	0	0	0.03	0.3	0.01	0.01	0.19	0.07
KY523104.1_AUL78507.1_MF405918.2_QKU34424.1_PUTATIVE_DNA_POLYMERASE_FAMILY_X_CDS	0.36	0.38	0.35	0.01	0.01	0.25	0.51	0.7	0.54	0.39	0.24
KY523104.1_AUL78508.1_MF405918.2_QKU34425.1_MG586_PROTEIN_CDS	0.26	0.42	0.35	0.11	0	0.21	0.53	0.28	0.53	0.36	0.12
KY523104.1_AUL78509.1_MF405918.2_QKU34426.1_PUTATIVE_DNA_POLYMERASE_FAMILY_X_CDS	0.14	0.13	0.07	0	0	0.04	0.1	0	0.08	0.28	0.08
KY523104.1_AUL78510.1_MF405918.2_QKU34427.1_PUTATIVE_ORFAN_CDS	0.2	0.35	0.19	0.08	0.01	0.15	0.42	0.09	0.12	0.39	0.15
KY523104.1_AUL78511.1_MF405918.2_QKU34428.1_PUTATIVE_ORFAN_CDS	0.39	0.58	0.39	0.26	0.03	0.38	0.23	0.13	0.46	0.39	0.45
KY523104.1_AUL78512.1_MF405918.2_QKU34429.1_UDP-N-ACETYLGLUCOSAMINE_2-EPIMERASE_CDS	0.23	0.08	0.34	0.01	0.01	0.22	0.47	0.36	0.51	0.38	0.08
KY523104.1_AUL78513.1_MF405918.2_QKU34430.1_PUTATIVE_ACYL-COA_OXIDASE_CDS	0.4	0.29	0.31	0.53	0.23	0.45	0.44	0.49	0.48	0.43	0.46
KY523104.1_AUL78514.1_MF405918.2_QKU34431.1_PUTATIVE_ORFAN_CDS	0.08	0.05	0.07	0	0	0.04	0.29	0.02	0.06	0.19	0.11
KY523104.1_AUL78515.1_MF405918.2_QKU34432.1_PUTATIVE_ORFAN_CDS	0.31	0.57	0.46	0.24	0.63	0.33	0.15	0.26	0.27	0.4	0.28
KY523104.1_AUL78516.1_MF405918.2_QKU34433.1_MG640_PROTEIN_CDS	0.21	0.24	0.46	0.08	0.45	0.18	0.25	0.06	0.02	0.46	0.17
KY523104.1_AUL78517.1_MF405918.2_QKU34434.1_HYPOTHETICAL_PROTEIN_CDS	0.41	0.33	0.35	0.29	0.55	0.36	0.15	0.33	0.24	0.4	0.44
KY523104.1_AUL78518.1_MF405918.2_QKU34435.1_PUTATIVE_ORFAN_CDS	0.04	0.04	0.06	0	0	0.04	0.02	0	0	0.19	0
KY523104.1_AUL78519.1_MF405918.2_QKU34436.1_PUTATIVE_ORFAN_CDS	0.07	0.07	0.04	0	0	0.03	0.26	0.01	0.01	0.15	0.1
KY523104.1_AUL78520.1_MF405918.2_QKU34437.1_HYPOTHETICAL_PROTEIN_CDS	0.25	0.27	0.32	0.33	0.18	0.31	0.23	0.36	0.38	0.45	0.21
KY523104.1_AUL78521.1_MF405918.2_QKU34438.1_CHRD_DOMAIN_PROTEIN_CDS	0.14	0.01	0.27	0	0	0.1	0.42	0.61	0.2	0.22	0.2
KY523104.1_AUL78522.1_MF405918.2_QKU34439.1_PUTATIVE_ORFAN_CDS	0.3	0.37	0.28	0.15	0.08	0.3	0.18	0.07	0.11	0.39	0.42
KY523104.1_AUL78523.1_MF405918.2_QKU34440.1_PUTATIVE_ORFAN_CDS	0.08	0.03	0.21	0.01	0	0.14	0.17	0.02	0.62	0.38	0.09
KY523104.1_AUL78524.1_MF405918.2_QKU34441.1_PUTATIVE_ORFAN_CDS	0.26	0.08	0.22	0.11	0.03	0.29	0.31	0.28	0.09	0.39	0.35

KY523104.1_AUL78525.1_MF405918.2_QKU34442.1_PUTATIVE_ORFAN_CDS	0.58	0.37	0.36	0.25	0.2	0.37	0.38	0.12	0.28	0.43	0.47
KY523104.1_AUL78526.1_MF405918.2_QKU34443.1_PUTATIVE_ORFAN_CDS	0.18	0.22	0.18	0.02	0.03	0.11	0.36	0.04	0.09	0.37	0.13
KY523104.1_AUL78527.1_MF405918.2_QKU34444.1_PUTATIVE_ORFAN_CDS	0.23	0.04	0.11	0.01	0	0.32	0.06	0.01	0.11	0.38	0.29
KY523104.1_AUL78528.1_MF405918.2_QKU34445.1_PUTATIVE_ORFAN_CDS	0.06	0.02	0.09	0	0.05	0.07	0.45	0.02	0.06	0.31	0.01
KY523104.1_AUL78529.1_MF405918.2_QKU34446.1_HYPOTHETICAL_PROTEIN_CDS	0.4	0.69	0.24	0	0.04	0.24	0.68	0.35	0.39	0	0.25
KY523104.1_AUL78530.1_MF405918.2_QKU34447.1_PUTATIVE_ORFAN_CDS	0.31	0.56	0.39	0.06	0.01	0.4	0.41	0.51	0.43	0.31	0.23
KY523104.1_AUL78531.1_MF405918.2_QKU34448.1_HYPOTHETICAL_PROTEIN_CDS	0.4	0.33	0.29	0.17	0.44	0.34	0.56	0.57	0.43	0.35	0.44
KY523104.1_AUL78532.1_MF405918.2_AUL79807.2_HYPOTHETICAL_PROTEIN_CDS	0.13	0.07	0.12	0	0	0.23	0.14	0.01	0.1	0.3	0.11
KY523104.1_AUL78533.1_MF405918.2_QKU34449.1_RIBOSOMAL-PROTEIN-ALANINE_N-ACETYLTRANSFERASE_CDS	0.32	0.59	0.4	0.12	0.3	0.35	0.38	0.31	0.34	0.27	0.34
KY523104.1_AUL78534.1_MF405918.2_QKU34450.1_PUTATIVE_ORFAN_CDS	0.27	0.21	0.23	0.09	0.01	0.26	0.11	0.04	0.35	0.41	0.32
KY523104.1_AUL78535.1_MF405918.2_QKU34451.1_HYPOTHETICAL_PROTEIN_CDS	0.45	0.07	0.28	0.32	0.03	0.28	0.32	0.14	0.46	0.46	0.47
KY523104.1_AUL78536.1_MF405918.2_QKU34452.1_PUTATIVE_ORFAN_CDS	0.26	0.03	0.23	0.01	0.02	0.15	0.16	0.53	0.08	0.3	0.36
KY523104.1_AUL78537.1_MF405918.2_QKU34453.1_PUTATIVE_TRANSPOSASE_CDS	0.62	0.3	0.66	0.35	0.23	0.43	0.16	0.08	0.41	0.39	0.46
KY523104.1_AUL78538.1_MF405918.2_QKU34454.1_PUTATIVE_RESOLVASE_CDS	0.67	0.21	0.29	0.27	0.04	0.29	0.42	0.07	0.17	0.43	0.47
KY523104.1_AUL78539.1_MF405918.2_QKU34455.1_HYPOTHETICAL_PROTEIN_CDS	1	0.24	0.13	0.23	0.01	0.31	0.22	0.01	0.47	0.23	0.85
KY523104.1_AUL78540.1_MF405918.2_QKU34456.1_PUTATIVE_ORFAN_CDS	0.27	0.27	0.28	0.13	0.47	0.31	0.21	0.15	0.17	0.44	0.31
KY523104.1_AUL78541.1_MF405918.2_QKU34457.1_PUTATIVE_ORFAN_CDS	0.58	0.01	0.13	0	0	0.08	0	0.01	0.01	0.25	0.59
KY523104.1_AUL78542.1_MF405918.2_QKU34458.1_HYPOTHETICAL_PROTEIN_CDS	0.36	0.28	0.29	0.2	0.09	0.3	0.29	0.26	0.51	0.45	0.41
KY523104.1_AUL78543.1_MF405918.2_QKU34459.1_BIFUNCTIONAL_AAA_FAMILY_ATPASE_CHAPERONE/TRANSLOCASI	0.56	0.64	0.28	0.17	0.62	0.48	0.54	0.09	0.34	0.4	0.51
KY523104.1_AUL78544.1_MF405918.2_QKU34460.1_PUTATIVE_ORFAN_CDS	0.18	0.1	0.13	0.01	0	0.17	0.16	0.01	0.25	0.41	0.15
KY523104.1_AUL78545.1_MF405918.2_QKU34461.1_HYPOTHETICAL_PROTEIN_CDS	0.5	0.25	0.32	0.23	0.09	0.32	0.41	0.26	0.16	0.42	0.45
KY523104.1_AUL78546.1_MF405918.2_QKU34462.1_HYPOTHETICAL_PROTEIN_CDS	0.16	0.02	0.24	0	0.52	0.16	0.1	0.06	0.59	0.4	0.03
KY523104.1_AUL78547.1_MF405918.2_QKU34463.1_GLYCOSYL_TRANSFERASE_CDS	0.34	0.41	0.53	0.97	0	0.98	0.29	0.16	0.42	0.42	0.24
KY523104.1_AUL78548.1_MF405918.2_AUL79821.2_PUTATIVE_ORFAN_CDS	0.18	0.5	0.41	0.13	0.01	0.37	0.26	0.43	0.2	0.31	0.24
KY523104.1_AUL78549.1_MF405918.2_QKU34464.1_PUTATIVE_ORFAN_CDS	0.38	0.31	0.29	0.25	0.49	0.37	0.26	0.2	0.46	0.51	0.43
KY523104.1_AUL78550.1_MF405918.2_QKU34465.1_HYPOTHETICAL_PROTEIN_CDS	0.35	0.5	0.41	0.17	0.03	0.43	0.28	0.49	0.37	0.29	0.42
KY523104.1_AUL78551.1_MF405918.2_QKU34466.1_PUTATIVE_ORFAN_CDS	0.39	0.64	0.39	0.28	0.11	0.32	0.27	0.22	0.43	0.26	0.39
KY523104.1_AUL78552.1_MF405918.2_QKU34467.1_HYPOTHETICAL_PROTEIN_CDS	0.29	0.09	0.19	0.04	0.05	0.22	0.24	0.11	0.07	0.39	0.39
KY523104.1_AUL78553.1_MF405918.2_QKU34468.1_PUTATIVE_ORFAN_CDS	0.13	0.19	0.15	0.01	0	0.07	0.03	0.1	0.03	0.26	0.28
KY523104.1_AUL78554.1_MF405918.2_QKU34469.1_HYPOXANTHINE_PHOSPHORIBOSYLTRANSFERASE_CDS	0.66	0.18	0.52	0.36	0.43	0.35	0.41	0.13	0.39	0.43	0.48
KY523104.1_AUL78555.1_MF405918.2_QKU34470.1_HYPOTHETICAL_PROTEIN_CDS	0.4	0.54	0.29	0.01	0	0.41	0.64	0.26	0.42	0.02	0.45
KY523104.1_AUL78556.1_MF405918.2_QKU34471.1_PROTEIN-TYROSINE_PHOSPHATASE_1_CDS	0.33	0.61	0.49	0.12	0.03	0.46	0.48	0.48	0.36	0.22	0.23
KY523104.1_AUL78557.1_MF405918.2_QKU34472.1_MG784_PROTEIN_CDS	0.48	0.26	0.27	0.21	0.08	0.29	0.4	0.05	0.13	0.43	0.44
KY523104.1_AUL78558.1_MF405918.2_QKU34473.1_PUTATIVE_ORFAN_CDS	0.24	0.39	0.34	0.13	0.46	0.31	0.43	0.37	0.25	0.41	0.32
KY523104.1_AUL78559.1_MF405918.2_QKU34474.1_HYPOTHETICAL_PROTEIN_CDS	0.1	0.08	0.17	0	0	0.06	0.12	0.44	0.09	0.25	0.23
KY523104.1_AUL78560.1_MF405918.2_QKU34475.1_HYPOTHETICAL_PROTEIN_CDS	0.12	0.3	0.13	0.02	0	0.06	0.21	0.01	0.02	0.33	0.09
KY523104.1_AUL78561.1_MF405918.2_QKU34476.1_HYPOTHETICAL_PROTEIN_CDS	0.26	0.29	0.49	0.11	0.07	0.31	0.19	0.5	0.4	0.44	0.25
KY523104.1_AUL78562.1_MF405918.2_QKU34477.1_HYPOTHETICAL_PROTEIN_CDS	0.4	0.78	0.61	0.86	0.89	0.21	0.55	0.13	0.31	0.01	0.2
KY523104.1_AUL78563.1_MF405918.2_QKU34478.1_MG321_PROTEIN_CDS	0.22	0.41	0.23	0.12	0.01	0.21	0.35	0.07	0.17	0.4	0.25
KY523104.1_AUL78564.1_MF405918.2_QKU34479.1_HYPOTHETICAL_PROTEIN_CDS	0.26	0.52	0.42	0.11	0	0.45	0.3	0.32	0.36	0.36	0.25
KY523104.1_AUL78565.1_MF405918.2_QKU34480.1_HYPOTHETICAL_PROTEIN_CDS	0.16	0.53	0.34	0.1	0	0.23	0.2	0.03	0.25	0.36	0.11
KY523104.1_AUL78566.1_MF405918.2_AUL79841.2_PHOSPHATIDATE_CYTIDYLYLTRANSFERASE_CDS	0.07	0.05	0.11	0	0	0.05	0.16	0.01	0.54	0.29	0.05

KY523104.1_AUL78567.1_MF405918.2_QKU34481.1_HYPOTHETICAL_PROTEIN_CDS	0.66	0.26	0.26	0.27	0.1	0.27	0.46	0.08	0.3	0.47	0.48
KY523104.1_AUL78568.1_MF405918.2_QKU34482.1_PUTATIVE_ORFAN_CDS	0.73	0.29	0.29	0.3	0.07	0.33	0.44	0.08	0.4	0.41	0.49
KY523104.1_AUL78569.1_MF405918.2_QKU34483.1_PUTATIVE_ORFAN_CDS	0.84	0.18	0.38	0.26	0.09	0.39	0.31	0.55	0.46	0.26	0.58
KY523104.1_AUL78570.1_MF405918.2_QKU34484.1_PUTATIVE_ORFAN_CDS	0.14	0.05	0.39	0.02	0	0.15	0.27	0.02	0.12	0.41	0.06
KY523104.1_AUL78571.1_MF405918.2_QKU34485.1_PUTATIVE_ORFAN_CDS	0.09	0.08	0.07	0	0	0.04	0.56	0.02	0.04	0.29	0.02
KY523104.1_AUL78572.1_MF405918.2_QKU34486.1_PUTATIVE_ORFAN_CDS	0.16	0.31	0.11	0.01	0	0.05	0.32	0	0.01	0.29	0.08
KY523104.1_AUL78573.1_MF405918.2_QKU34487.1_UV-DAMAGE_ENDONUCLEASE_CDS	0.41	0.69	0.35	0.2	0.03	0.47	0.51	0.36	0.44	0.12	0.42
KY523104.1_AUL78574.1_MF405918.2_QKU34488.1_PUTATIVE_ORFAN_CDS	0.32	0.69	0.41	0.12	0.02	0.23	0.04	0.15	0.38	0.19	0.19
KY523104.1_AUL78575.1_MF405918.2_QKU34489.1_HYPOTHETICAL_PROTEIN_CDS	0.33	0.21	0.37	0.5	0.24	0.54	0.5	0.32	0.48	0.47	0.41
KY523104.1_AUL78576.1_MF405918.2_QKU34490.1_PUTATIVE_UDP-N-ACETYLGLUCOSAMINE_PYROPHOSPHORYLASE_	0.27	0.05	0.27	0.22	0.04	0.51	0.27	0.15	0.45	0.39	0.37
KY523104.1_AUL78577.1_MF405918.2_QKU34491.1_HYPOTHETICAL_PROTEIN_CDS	0.4	0.62	0.47	0.92	0.51	0.49	0.46	0.36	0.42	0.38	0.41
KY523104.1_AUL78577.1_MF405918.2_QKU34491.1_HYPOTHETICAL_PROTEIN_CDS	0.25	0.08	0.27	0.14	0.05	0.51	0.38	0.1	0.34	0.46	0.18
KY523104.1_AUL78578.1_MF405918.2_QKU34492.1_PUTATIVE_ORFAN_CDS	0.25	0.08	0.27	0.14	0.05	0.51	0.38	0.1	0.34	0.46	0.18
KY523104.1_AUL78579.1_MF405918.2_QKU34493.1_PUTATIVE_ORFAN_CDS	0.15	0.54	0.33	0.09	0	0.18	0.26	0.04	0.12	0.35	0.09
KY523104.1_AUL78580.1_MF405918.2_QKU34494.1_PUTATIVE_ORFAN_CDS	0.25	0.26	0.24	0.07	0	0.46	0.12	0.04	0.23	0.43	0.27
KY523104.1_AUL78581.1_MF405918.2_QKU34495.1_PUTATIVE_ANKYRIN_REPEAT_PROTEIN_CDS	0.5	0.19	0.39	0.29	0.07	0.31	0.38	0.07	0.24	0.44	0.47
KY523104.1_AUL78582.1_MF405918.2_QKU34496.1_PUTATIVE_ORFAN_CDS	0.53	0.1	0.24	0.21	0.01	0.23	0.25	0.08	0.07	0.36	0.47
KY523104.1_AUL78583.1_MF405918.2_QKU34497.1_PUTATIVE_LIPOCALIN_CDS	0.17	0.49	0.24	0.1	0.06	0.22	0.45	0.14	0.12	0.35	0.12
KY523104.1_AUL78584.1_MF405918.2_QKU34498.1_MEMBRANE_PROTEIN_CDS	0.32	0.32	0.36	0.18	0.2	0.31	0.09	0.17	0.5	0.43	0.37
KY523104.1_AUL78585.1_MF405918.2_QKU34499.1_CYSTEINE_DESULFURASE_SUFS_FAMILY_CDS	0.37	0.61	0.45	0.17	0.14	0.37	0.48	0.34	0.45	0.25	0.33
KY523104.1_AUL78586.1_MF405918.2_QKU34500.1_PUTATIVE_ORFAN_CDS	0.32	0.26	0.31	0.21	0.2	0.45	0.48	0.23	0.49	0.41	0.4
KY523104.1_AUL78587.1_MF405918.2_QKU34501.1_TLR_6FP_PROTEIN_CDS	0.07	0.02	0.13	0	0.25	0.09	0	0.01	0.01	0.38	0.04
KY523104.1_AUL78588.1_MF405918.2_QKU34502.1_HYPOTHETICAL_PROTEIN_CDS	0.71	0.21	0.38	0.34	0.55	0.35	0.22	0.22	0.42	0.46	0.47
KY523104.1_AUL78589.1_MF405918.2_QKU34503.1_HYPOTHETICAL_PROTEIN_CDS	0.39	0.67	0.42	0.38	0.11	0.34	0.2	0.19	0.47	0.24	0.29
KY523104.1_AUL78590.1_MF405918.2_QKU34504.1_HYPOTHETICAL_PROTEIN_CDS	0.46	0.3	0.32	0.27	0.52	0.41	0.42	0.23	0.36	0.52	0.45
KY523104.1_AUL78591.1_MF405918.2_AUL79864.2_HYPOTHETICAL_PROTEIN_CDS	0.39	0.57	0.33	0.29	0.08	0.35	0.23	0.18	0.51	0.5	0.39
KY523104.1_AUL78592.1_MF405918.2_AUL79865.2_HYPOTHETICAL_PROTEIN_CDS	0.3	0.18	0.27	0.15	0	0.61	0.42	0.15	0.42	0.41	0.37
KY523104.1_AUL78593.1_MF405918.2_QKU34505.1_HYPOTHETICAL_PROTEIN_CDS	0.39	0.4	0.31	0.28	0.42	0.39	0.25	0.17	0.48	0.44	0.43
KY523104.1_AUL78594.1_MF405918.2_QKU34506.1_PUTATIVE_ORFAN_CDS	0.3	0.27	0.39	0.42	0.26	0.59	0.55	0.29	0.39	0.34	0.28
KY523104.1_AUL78595.1_MF405918.2_QKU34507.1_GLYCOSYL_TRANSFERASE_CDS	0.22	0.3	0.29	0.08	0.17	0.26	0.55	0.35	0.33	0.25	0.2
KY523104.1_AUL78596.1_MF405918.2_QKU34508.1_PUTATIVE_ORFAN_CDS	0.22	0.33	0.18	0.05	0.08	0.09	0.23	0.03	0.02	0.4	0.18
KY523104.1_AUL78597.1_MF405918.2_QKU34509.1_PUTATIVE_ORFAN_CDS	0.61	0.02	0.23	0.27	0.01	0.27	0.03	0.07	0.46	0.4	0.49
KY523104.1_AUL78598.1_MF405918.2_QKU34510.1_PUTATIVE_ORFAN_CDS	0.39	0.33	0.32	0.15	0.1	0.3	0.29	0.35	0.06	0.36	0.42
KY523104.1_AUL78599.1_MF405918.2_QKU34511.1_HYPOTHETICAL_PROTEIN_CDS	0.4	0.68	0.45	0.34	0.03	0.35	0.41	0.43	0.49	0.19	0.32
KY523104.1_AUL78600.1_MF405918.2_QKU34512.1_HYPOTHETICAL_PROTEIN_CDS	0.21	0.3	0.18	0.05	0.39	0.16	0.12	0.04	0.04	0.38	0.24
KY523104.1_AUL78601.1_MF405918.2_QKU34513.1_HYPOTHETICAL_PROTEIN_CDS	0.3	0.07	0.4	0.1	0.06	0.44	0.31	0.36	0.47	0.52	0.35
KY523104.1_AUL78600.1_MF405918.2_QKU34512.1_HYPOTHETICAL_PROTEIN_CDS	0.05	0.02	0.02	0	0	0.01	0.09	0	0	0.14	0.33
KY523104.1_AUL78602.1_MF405918.2_QKU34514.1_HYPOTHETICAL_PROTEIN_CDS	0.4	0.44	0.29	0.16	0.39	0.29	0.58	0.37	0.48	0.24	0.45
KY523104.1_AUL78603.1_MF405918.2_QKU34515.1_PUTATIVE_ORFAN_CDS	0.42	0.04	0.38	0	0.51	0.44	0	0.25	0.05	0.4	0.11
KY523104.1_AUL78604.1_MF405918.2_QKU34516.1_PUTATIVE_ORFAN_CDS	0.14	0.03	0.13	0.01	0.02	0.06	0.28	0.02	0.38	0.33	0.19
KY523104.1_AUL78605.1_MF405918.2_AUL79879.2_CATHEPSIN_LLIKE_CYSTEINE_PROTEASE_CDS	0.43	0.47	0.32	0.26	0.08	0.39	0.5	0.17	0.36	0.37	0.47
KY523104.1_AUL78606.1_MF405918.2_QKU34517.1_PUTATIVE_ORFAN_CDS	0.22	0.3	0.37	0.08	0.25	0.3	0.3	0.32	0.09	0.42	0.19

KY523104.1_AUL78607.1_MF405918.2_QKU34518.1_2OG-FE(II)_OXYGENASE_CDS	0.41	0.27	0.4	0.53	0.41	0.37	0.1	0.2	0.27	0.44	0.45
KY523104.1_AUL78608.1_MF405918.2_QKU34519.1_PUTATIVE_METHYLATED-DNA-PROTEIN-CYSTEINE_METHYLTRANSF	0.19	0.34	0.31	0.11	0	0.48	0.05	0.02	0.25	0.44	0.13
KY523104.1_AUL78609.1_MF405918.2_QKU34520.1_PUTATIVE_GUANOSINE-3'_5'-BIS(DIPHOSPHATE)_3'-PYROPHOSPH	0.26	0.2	0.28	0.07	0	0.35	0.57	0.22	0.38	0.29	0.26
KY523104.1_AUL78610.1_MF405918.2_QKU34521.1_PUTATIVE_ORFAN_CDS	0.19	0.48	0.4	0.04	0	0.56	0.27	0.34	0.38	0.4	0.12
KY523104.1_AUL78611.1_MF405918.2_QKU34522.1_PUTATIVE_ORFAN_CDS	0.14	0.08	0.1	0	0.01	0.05	0.18	0.03	0.14	0.24	0.28
KY523104.1_AUL78612.1_MF405918.2_QKU34523.1_PUTATIVE_ORFAN_CDS	0.13	0.3	0.23	0	0	0.12	0.48	0.54	0.1	0.2	0.2
KY523104.1_AUL78613.1_MF405918.2_QKU34524.1_PUTATIVE_ORFAN_CDS	0.66	0.38	0.38	0.3	0.56	0.36	0.4	0.49	0.29	0.42	0.46
KY523104.1_AUL78614.1_MF405918.2_QKU34525.1_PUTATIVE_ORFAN_CDS	0.8	0.01	0.23	0.16	0	0.2	0.15	0.04	0.13	0.36	0.48
KY523104.1_AUL78615.1_MF405918.2_QKU34526.1_HYPOTHETICAL_PROTEIN_CDS	0.19	0.4	0.22	0.07	0.01	0.17	0.5	0.1	0.03	0.36	0.18
KY523104.1_AUL78616.1_MF405918.2_QKU34527.1_PUTATIVE_ORFAN_CDS	0.66	0.03	0.2	0.37	0.04	0.19	0.19	0.11	0.47	0.42	0.54
KY523104.1_AUL78617.1_MF405918.2_QKU34528.1_HYPOTHETICAL_PROTEIN_CDS	0.3	0.07	0.34	0.09	0.54	0.33	0.33	0.2	0.13	0.45	0.35
KY523104.1_AUL78618.1_MF405918.2_QKU34529.1_HYPOTHETICAL_PROTEIN_CDS	0.81	0.75	0.53	0.21	0.14	0.48	0.16	0.23	0.44	0.27	0.56
KY523104.1_AUL78619.1_MF405918.2_QKU34530.1_THREONYL-TRNA_SYNTHETASE_CDS	0.73	0.35	0.15	0.18	0.43	0.12	0.56	0.03	0.4	0.5	0.58
KY523104.1_AUL78620.1_MF405918.2_QKU34531.1_HYPOTHETICAL_PROTEIN_CDS	0.16	0.52	0.34	0.08	0	0.24	0.33	0.23	0.21	0.33	0.13
KY523104.1_AUL78621.1_MF405918.2_QKU34532.1_UBIQUITIN-CONJUGATING_ENZYME_CDS	0.52	0.45	0.34	0.25	0.62	0.38	0.43	0.25	0.4	0.62	0.46
KY523104.1_AUL78622.1_MF405918.2_QKU34533.1_HYPOTHETICAL_PROTEIN_CDS	0.28	0.24	0.29	0.15	0.01	0.41	0.27	0.05	0.26	0.43	0.41
KY523104.1_AUL78623.1_MF405918.2_QKU34534.1_HYPOTHETICAL_PROTEIN_CDS	0.35	0.6	0.4	0.22	0.1	0.42	0.32	0.32	0.45	0.31	0.38
KY523104.1_AUL78624.1_MF405918.2_QKU34535.1_HYPOTHETICAL_PROTEIN_CDS	0.37	0.04	0.23	0.01	0.02	0.37	0.57	0.1	0.35	0.19	0.39
KY523104.1_AUL78625.1_MF405918.2_QKU34536.1_HYPOTHETICAL_PROTEIN_CDS	0.17	0.06	0.25	0	0.02	0.25	0.55	0.12	0.11	0.31	0.06
KY523104.1_AUL78626.1_MF405918.2_QKU34537.1_HYPOTHETICAL_PROTEIN_CDS	0.4	0.67	0.39	0.12	0.49	0.43	0.43	0.49	0.44	0.16	0.31
KY523104.1_AUL78627.1_MF405918.2_QKU34538.1_HYPOTHETICAL_PROTEIN_CDS	0.22	0.5	0.36	0.05	0	0.27	0.33	0.5	0.36	0.32	0.21
KY523104.1_AUL78628.1_MF405918.2_QKU34539.1_HYPOTHETICAL_PROTEIN_CDS	0.4	0.79	0.29	0.25	0.43	0.05	0.34	0.04	0.38	0	0.26
KY523104.1_AUL78629.1_MF405918.2_QKU34540.1_PUTATIVE_PROTEIN_KINASE_CDS	0.45	0.61	0.26	0	0.07	0.29	0.52	0.82	0.54	0	0.42
KY523104.1_AUL78630.1_MF405918.2_QKU34541.1_IG_FAMILY_PROTEIN_CDS	0.4	0.66	0.3	0.36	0.05	0.7	0.42	0.26	0.46	0.41	0.41
KY523104.1_AUL78631.1_MF405918.2_QKU34542.1_HYPOTHETICAL_PROTEIN_CDS	0.37	0.01	0.24	0	0	0.47	0.58	0.08	0.36	0.23	0.31
KY523104.1_AUL78632.1_MF405918.2_QKU34543.1_PUTATIVE_ORFAN_CDS	0.31	0.06	0.19	0.02	0.03	0.13	0.16	0.1	0.05	0.29	0.52
KY523104.1_AUL78633.1_MF405918.2_QKU34544.1_HYPOTHETICAL_PROTEIN_CDS	0.48	0.26	0.32	0.3	0.23	0.32	0.27	0.1	0.44	0.44	0.46
KY523104.1_AUL78634.1_MF405918.2_QKU34545.1_HYPOTHETICAL_PROTEIN_CDS	0.42	0.37	0.24	0.35	0.14	0.2	0.44	0.48	0.58	0.46	0.47
KY523104.1_AUL78635.1_MF405918.2_QKU34546.1_PUTATIVE_ORFAN_CDS	0.44	0.62	0.21	0.08	0.14	0.26	0.59	0.15	0.47	0.05	0.5
KY523104.1_AUL78636.1_MF405918.2_QKU34547.1_PUTATIVE_ORFAN_CDS	0.31	0.23	0.4	0.4	0.13	0.27	0.31	0.57	0.52	0.47	0.25
KY523104.1_AUL78635.1_MF405918.2_QKU34546.1_PUTATIVE_ORFAN_CDS	0.31	0.23	0.4	0.4	0.13	0.27	0.31	0.57	0.52	0.47	0.25
KY523104.1_AUL78637.1_MF405918.2_QKU34548.1_CHEMOTAXIS_CDS	0.33	0.42	0.36	0.21	0.02	0.44	0.31	0.16	0.42	0.39	0.43
KY523104.1_AUL78638.1_MF405918.2_QKU34549.1_HYPOTHETICAL_PROTEIN_CDS	0.4	0.42	0.47	0.99	0.46	0.44	0.27	0.17	0.41	0.38	0.45
KY523104.1_AUL78639.1_MF405918.2_QKU34550.1_HYPOTHETICAL_PROTEIN_CDS	0.28	0.25	0.35	0.2	0.05	0.35	0.29	0.09	0.37	0.42	0.39
KY523104.1_AUL78640.1_MF405918.2_QKU34551.1_MG257_PROTEIN_CDS	0.29	0.09	0.24	0.08	0.41	0.23	0.29	0.11	0.13	0.41	0.35
KY523104.1_AUL78641.1_MF405918.2_QKU34552.1_PUTATIVE_ORFAN_CDS	0.01	0.04	0.09	0	0	0.05	0	0	0.09	0.28	0
KY523104.1_AUL78642.1_MF405918.2_QKU34553.1_STRUCTURAL_PPIASE-LIKE_PROTEIN_CDS	0.4	0.21	0.3	0.09	0.12	0.55	0.63	0.18	0.44	0.11	0.47
KY523104.1_AUL78643.1_MF405918.2_QKU34554.1_PUTATIVE_ORFAN_CDS	0.12	0	0.04	0	0	0.02	0.04	0	0.01	0.11	0.19
KY523104.1_AUL78643.1_MF405918.2_QKU34554.1_PUTATIVE_ORFAN_CDS	0.28	0.18	0.13	0.02	0	0.19	0.13	0.02	0.03	0.35	0.38
KY523104.1_AUL78644.1_MF405918.2_QKU34555.1_PUTATIVE_ORFAN_CDS	0.72	0.58	0.41	0.39	0.3	0.69	0.32	0.09	0.45	0.47	0.55
KY523104.1_AUL78645.1_MF405918.2_QKU34556.1_HYPOTHETICAL_PROTEIN_CDS	0.24	0.23	0.22	0.08	0.43	0.28	0.19	0.05	0.07	0.44	0.27
KY523104.1_AUL78646.1_MF405918.2_QKU34557.1_PROTEIN_OF_HYPOTHETICAL_PROTEIN_FUNCTION_DUF437_CDS	0.27	0.18	0.24	0.16	0.03	0.3	0.44	0.08	0.1	0.4	0.38

KY523104.1_AUL78647.1_MF405918.2_QKU34558.1_PUTATIVE_PHOSPHOGLYCERATE_MUTASE_FAMILY_PROTEIN_CDS	0.22	0.03	0.75	0.07	0	0.2	0.19	0.05	0.11	0.41	0.07
KY523104.1_AUL78648.1_MF405918.2_QKU34559.1_PUTATIVE_ORFAN_CDS	0.36	0.03	0.28	0.11	0.25	0.26	0.15	0.51	0.2	0.32	0.43
KY523104.1_AUL78649.1_MF405918.2_QKU34560.1_HYPOTHETICAL_PROTEIN_CDS	0.23	0.5	0.43	0.1	0.79	0.31	0.06	0.25	0.09	0.45	0.18
KY523104.1_AUL78650.1_MF405918.2_QKU34561.1_PUTATIVE_ORFAN_CDS	0.21	0.41	0.35	0.09	0	0.48	0.21	0.07	0.47	0.5	0.09
KY523104.1_AUL78651.1_MF405918.2_QKU34562.1_PUTATIVE_ORFAN_CDS	0.4	0.31	0.4	0.27	0.56	0.43	0.33	0.33	0.28	0.54	0.44
KY523104.1_AUL78652.1_MF405918.2_QKU34563.1_PUTATIVE_ORFAN_CDS	0.06	0.05	0.05	0	0	0.04	0.43	0.02	0.02	0.16	0.05
KY523104.1_AUL78653.1_MF405918.2_QKU34564.1_CYSTEINYL-TRNA_SYNTHETASE_CDS	0.32	0.31	0.37	0.16	0.22	0.42	0.38	0.54	0.48	0.43	0.37
KY523104.1_AUL78654.1_MF405918.2_QKU34565.1_HYPOTHETICAL_PROTEIN_CDS	0.33	0.22	0.33	0.04	0	0.42	0.57	0.54	0.38	0.24	0.31
KY523104.1_AUL78655.1_MF405918.2_QKU34566.1_PUTATIVE_ORFAN_CDS	0.42	0.71	0.54	0.44	0.64	0.38	0.15	0.26	0.37	0.31	0.4
KY523104.1_AUL78656.1_MF405918.2_QKU34567.1_MIMIVIRUS_TRANSLATION_INITIATION_FACTOR_2_SUBUNIT_ALP	0.3	0.47	0.31	0.15	0	0.28	0.45	0.17	0.46	0.36	0.37
KY523104.1_AUL78657.1_MF405918.2_QKU34568.1_PUTATIVE_ORFAN_CDS	0.21	0.29	0.31	0.06	0.01	0.22	0.39	0.41	0.33	0.43	0.18
KY523104.1_AUL78658.1_MF405918.2_QKU34569.1_PUTATIVE_UBIQUITIN-CONJUGATING_ENZYME_E2_CDS	0.53	0.65	0.39	0.26	0.43	0.42	0.25	0.15	0.43	0.35	0.47
KY523104.1_AUL78659.1_MF405918.2_QKU34570.1_HYPOTHETICAL_PROTEIN_CDS	0.26	0.25	0.22	0.25	0.04	0.26	0.2	0.04	0.15	0.42	0.36
KY523104.1_AUL78660.1_MF405918.2_QKU34571.1_HYPOTHETICAL_PROTEIN_CDS	0.27	0.38	0.33	0.12	0	0.25	0.55	0.24	0.48	0.25	0.23
KY523104.1_AUL78661.1_MF405918.2_QKU34572.1_HYPOTHETICAL_PROTEIN_CDS	0.4	0.33	0.21	0.04	0.04	0.17	0.64	0.16	0.48	0.05	0.41
KY523104.1_AUL78662.1_MF405918.2_QKU34573.1_HYPOTHETICAL_PROTEIN_CDS	0.59	0.36	0.33	0.22	0.32	0.66	0.4	0.67	0.5	0.29	0.46
KY523104.1_AUL78663.1_MF405918.2_QKU34574.1_PUTATIVE_ORFAN_CDS	0.35	0.19	0.26	0.15	0.42	0.27	0.19	0.07	0.11	0.44	0.42
KY523104.1_AUL78664.1_MF405918.2_QKU34575.1_INHIBITOR_(BI)-1/YCCA-LIKE_PROTEIN_FAMILY_CDS	0.21	0.25	0.33	0.05	0.41	0.32	0.25	0.34	0.27	0.43	0.13
KY523104.1_AUL78665.1_MF405918.2_QKU34576.1_HYPOTHETICAL_PROTEIN_CDS	0.17	0.36	0.25	0.05	0.01	0.43	0.15	0.02	0.06	0.35	0.17
KY523104.1_AUL78666.1_MF405918.2_QKU34577.1_PUTATIVE_ORFAN_CDS	0.4	0.62	0.02	0	0.14	0.07	0.76	0.24	0.32	0	0.48
KY523104.1_AUL78667.1_MF405918.2_QKU34578.1_PUTATIVE_ORFAN_CDS	0.14	0.04	0.12	0	0	0.05	0.23	0.02	0.34	0.23	0.16
KY523104.1_AUL78668.1_MF405918.2_QKU34579.1_HYPOTHETICAL_PROTEIN_CDS	0.36	0.1	0.32	0.64	0.27	0.43	0.1	0.25	0.45	0.44	0.44
KY523104.1_AUL78669.1_MF405918.2_AUL79942.2_PUTATIVE_ANKYRIN_REPEAT_PROTEIN_CDS	0.35	0.43	0.36	0.45	0.02	0.63	0.27	0.23	0.42	0.44	0.4
KY523104.1_AUL78670.1_MF405918.2_QKU34580.1_PUTATIVE_GLUTAMINE_AMIDOTRANSFERASE-LIKE_PROTEIN_CDS	0.39	0.61	0.43	0.32	0.46	0.37	0.25	0.37	0.43	0.29	0.34
KY523104.1_AUL78671.1_MF405918.2_QKU34581.1_ARGINYL_TRNA-SYNTHETASE_CDS	0.37	0.26	0.35	0.45	0.29	0.38	0.24	0.36	0.49	0.44	0.42
KY523104.1_AUL78672.1_MF405918.2_QKU34582.1_PUTATIVE_OVARIAN_CANCER-ASSOCIATED_2_PROTEIN_CDS	0.27	0.15	0.25	0.08	0.12	0.22	0.22	0.07	0.14	0.44	0.34
KY523104.1_AUL78673.1_MF405918.2_QKU34583.1_PUTATIVE_ORFAN_CDS	0.26	0.11	0.28	0.08	0.04	0.25	0.33	0.42	0.1	0.4	0.32
KY523104.1_AUL78674.1_MF405918.2_QKU34584.1_HYPOTHETICAL_PROTEIN_CDS	0.35	0.54	0.41	0.14	0.21	0.33	0.06	0.08	0.25	0.37	0.41
KY523104.1_AUL78675.1_MF405918.2_QKU34585.1_LEUCYL-TRNA_SYNTHETASE_CDS	0.4	0.66	0.38	0.22	0.12	0.45	0.44	0.52	0.44	0.17	0.41
KY523104.1_AUL78676.1_MF405918.2_QKU34586.1_HYPOTHETICAL_PROTEIN_CDS	0.21	0.03	0.27	0.7	0	0.24	0.02	0.05	0.47	0.38	0.09
KY523104.1_AUL78677.1_MF405918.2_QKU34587.1_MACRO_DOMAIN_CONTAINING_PROTEIN_CDS	0.4	0.27	0.29	0.23	0.07	0.31	0.45	0.13	0.17	0.42	0.45
KY523104.1_AUL78678.1_MF405918.2_QKU34588.1_HYPOTHETICAL_PROTEIN_CDS	0.25	0.38	0.33	0.4	0.37	0.31	0.5	0.32	0.33	0.29	0.21
KY523104.1_AUL78679.1_MF405918.2_QKU34589.1_HYPOTHETICAL_PROTEIN_CDS	0.39	0.23	0.45	0.01	0.66	0.32	0.02	0.38	0.37	0.59	0.32
KY523104.1_AUL78680.1_MF405918.2_QKU34590.1_HYPOTHETICAL_PROTEIN_CDS	0.79	0.14	0.31	0.32	0	0.3	0.02	0.12	0.4	0.37	0.52
KY523104.1_AUL78681.1_MF405918.2_QKU34591.1_HYPOTHETICAL_PROTEIN_CDS	0.26	0.21	0.32	0.13	0.12	0.41	0.2	0.12	0.48	0.44	0.32
KY523104.1_AUL78682.1_MF405918.2_QKU34592.1_HYPOTHETICAL_PROTEIN_CDS	0.21	0.24	0.18	0.05	0	0.15	0.33	0.04	0.19	0.41	0.15
KY523104.1_AUL78683.1_MF405918.2_QKU34593.1_PUTATIVE_ORFAN_CDS	0.19	0.12	0.08	0	0.03	0.05	0.28	0.02	0.03	0.22	0.1
KY523104.1_AUL78684.1_MF405918.2_QKU34594.1_PUTATIVE_ORFAN_CDS	0.44	0.07	0.4	0	0.84	0.38	0.15	0.28	0.19	0.56	0.34
KY523104.1_AUL78685.1_MF405918.2_QKU34595.1_HYPOTHETICAL_PROTEIN_CDS	0.77	0.55	0.01	0.09	0.11	0.13	0.62	0.02	0.73	0.55	0.67
KY523104.1_AUL78686.1_MF405918.2_QKU34596.1_HD_SUPERFAMILY_HYDROLASE_CDS	0.53	0.18	0.32	0.36	0.2	0.34	0.4	0.17	0.32	0.48	0.47
KY523104.1_AUL78687.1_MF405918.2_QKU34597.1_PUTATIVE_CHEMOTAXIS_PROTEIN_CHED_CDS	0.26	0.53	0.43	0.44	0.11	0.37	0.35	0.44	0.3	0.29	0.27
KY523104.1_AUL78688.1_MF405918.2_QKU34598.1_PUTATIVE_ORFAN_CDS	0.22	0.33	0.33	0.17	0.02	0.25	0.4	0.43	0.24	0.41	0.19

KY523104.1_AUL78689.1_MF405918.2_QKU34599.1_DEHA2D17908P_CDS	0.4	0	0.33	0	0	0.02	0.21	0.82	0.41	0.03	0.43
KY523104.1_AUL78690.1_MF405918.2_QKU34600.1_HYPOTHETICAL_PROTEIN_CDS	0.47	0.12	0.28	0.45	0.42	0.37	0.25	0.33	0.48	0.44	0.47
KY523104.1_AUL78691.1_MF405918.2_QKU34601.1_BIFUNCTIONAL_METALLOPROTEASE_UBIQUITIN-PROTEIN_LIGASE_	0.4	0.64	0.37	0.06	0.09	0.57	0.53	0.48	0.47	0.19	0.34
KY523104.1_AUL78692.1_MF405918.2_QKU34602.1_PUTATIVE_ORFAN_CDS	0.65	0.07	0.26	0.17	0.18	0.29	0.53	0.12	0.12	0.37	0.47
KY523104.1_AUL78693.1_MF405918.2_QKU34603.1_TONB_BOX-LIKE_PROTEIN_CDS	0.15	0.02	0.35	0.02	0.45	0.29	0.47	0.11	0.23	0.4	0.04
KY523104.1_AUL78694.1_MF405918.2_QKU34604.1_PUTATIVE_ORFAN_CDS	0.39	0.53	0.29	0.08	0	0.43	0.55	0.2	0.4	0.23	0.45
KY523104.1_AUL78695.1_MF405918.2_QKU34605.1_MG275_PROTEIN_CDS	0.23	0.12	0.37	0.06	0.42	0.44	0.35	0.35	0.29	0.45	0.07
KY523104.1_AUL78696.1_MF405918.2_QKU34606.1_PUTATIVE_ORFAN_CDS	0.26	0.07	0.32	0.13	0.06	0.35	0.52	0.11	0.09	0.36	0.37
KY523104.1_AUL78697.1_MF405918.2_QKU34607.1_PUTATIVE_ORFAN_CDS	0.4	0.67	0.39	0.32	0.02	0.34	0.22	0.31	0.46	0.26	0.39
KY523104.1_AUL78698.1_MF405918.2_QKU34608.1_HYPOTHETICAL_PROTEIN_CDS	0.37	0.33	0.41	0.13	0.2	0.43	0.29	0.55	0.32	0.19	0.42
KY523104.1_AUL78699.1_MF405918.2_QKU34609.1_GLYCYL_TRNA-SYNTHETASE_CDS	0.34	0.38	0.42	0.27	0.19	0.35	0.47	0.26	0.49	0.37	0.37
KY523104.1_AUL78700.1_MF405918.2_QKU34610.1_HYPOTHETICAL_PROTEIN_CDS	0.41	0.68	0.29	0.04	0.02	0.44	0.55	0.75	0.54	0.08	0.36
KY523104.1_AUL78701.1_MF405918.2_QKU34611.1_HYPOTHETICAL_PROTEIN_CDS	0.4	0.33	0.22	0.08	0.24	0.21	0.6	0.19	0.41	0.07	0.41
KY523104.1_AUL78702.1_MF405918.2_QKU34612.1_HYPOTHETICAL_PROTEIN_CDS	0.5	0.13	0.31	0.3	0.04	0.45	0.21	0.14	0.43	0.37	0.46
KY523104.1_AUL78703.1_MF405918.2_QKU34613.1_A32-LIKE_PACKAGING_ATPASE_CDS	0.4	0.74	0.28	0.19	0.06	0.05	0.52	0.12	0.39	0	0.06
KY523104.1_AUL78704.1_MF405918.2_QKU34614.1_TYROSINE-TRNA_SYNTHETASE_CDS	0.4	0.13	0.31	0.04	0.01	0.62	0.61	0.44	0.41	0.32	0.28
KY523104.1_AUL78705.1_MF405918.2_QKU34615.1_AAEL017480-PA_CDS	0.46	0.57	0.39	0.21	0.48	0.4	0.34	0.16	0.23	0.39	0.44
KY523104.1_AUL78706.1_MF405918.2_QKU34616.1_HYPOTHETICAL_PROTEIN_CDS	0.21	0.02	0.41	0.01	0.01	0.34	0.52	0.51	0.12	0.36	0.14
KY523104.1_AUL78707.1_MF405918.2_QKU34617.1_HYPOTHETICAL_PROTEIN_CDS	0.78	0.14	0.39	0.24	0.16	0.46	0.21	0.27	0.36	0.34	0.48
KY523104.1_AUL78708.1_MF405918.2_QKU34618.1_PUTATIVE_ORFAN_CDS	0.25	0.25	0.34	0.09	0.45	0.31	0.34	0.15	0.15	0.49	0.19
KY523104.1_AUL78709.1_MF405918.2_QKU34619.1_HYPOTHETICAL_PROTEIN_CDS	0.4	0.43	0.4	0.1	0.64	0.28	0.22	0.52	0.48	0.81	0.39
KY523104.1_AUL78710.1_MF405918.2_QKU34620.1_MIMIVIRUS_PEPTIDE_CHAIN_RELEASE_FACTOR_ERF1_CDS	0.21	0.17	0.32	0.09	0.25	0.24	0.38	0.14	0.48	0.41	0.08
KY523104.1_AUL78711.1_MF405918.2_QKU34621.1_HYPOTHETICAL_PROTEIN_CDS	0.62	0.72	0.4	0.27	0.48	0.44	0.35	0.17	0.41	0.21	0.5
KY523104.1_AUL78712.1_MF405918.2_QKU34622.1_HYPOTHETICAL_PROTEIN_CDS	0.11	0.02	0.14	0	0.41	0.09	0.1	0.02	0.35	0.39	0.04
KY523104.1_AUL78713.1_MF405918.2_QKU34623.1_HYPOTHETICAL_PROTEIN_CDS	0.22	0.21	0.27	0.06	0.04	0.25	0.51	0.13	0.13	0.33	0.17
KY523104.1_AUL78714.1_MF405918.2_QKU34624.1_MIMIVIRUS_ELONGATION_FACTOR_AEF-2_CDS	0.4	0.73	0.53	0.03	0.55	0.6	0.62	0.72	0.42	0	0.19
KY523104.1_AUL78715.1_MF405918.2_QKU34625.1_PUTATIVE_ORFAN_CDS	0.28	0.17	0.2	0.17	0.02	0.22	0.26	0.04	0.13	0.41	0.35
KY523104.1_AUL78716.1_MF405918.2_QKU34626.1_PUTATIVE_ORFAN_CDS	0.13	0.09	0.09	0	0.01	0.05	0.21	0.04	0.06	0.22	0.22
KY523104.1_AUL78717.1_MF405918.2_QKU34627.1_HYPOTHETICAL_PROTEIN_CDS	0.4	0.19	0.1	0.01	0	0.34	0.67	0.11	0.32	0.04	0.35
KY523104.1_AUL78718.1_MF405918.2_QKU34628.1_MG283_PROTEIN_CDS	0.73	0.26	0.24	0.44	0.11	0.3	0.42	0.05	0.43	0.43	0.52
KY523104.1_AUL78719.1_MF405918.2_QKU34629.1_PUTATIVE_CFXQ-LIKE_PROTEIN_CDS	0.4	0.57	0.21	0.01	0	0.29	0.61	0.44	0.4	0.09	0.44
KY523104.1_AUL78720.1_MF405918.2_QKU34630.1_HYPOTHETICAL_PROTEIN_CDS	0.49	0.62	0.3	0.29	0.47	0.63	0.41	0.28	0.47	0.43	0.47
KY523104.1_AUL78721.1_MF405918.2_QKU34631.1_PUTATIVE_ORFAN_CDS	0.39	0.07	0.19	0.01	0.06	0.35	0.58	0.09	0.35	0.13	0.41
KY523104.1_AUL78722.1_MF405918.2_QKU34632.1_PUTATIVE_ORFAN_CDS	0.37	0.24	0.33	0.09	0.02	0.28	0.09	0.53	0.26	0.29	0.41
KY523104.1_AUL78723.1_MF405918.2_QKU34633.1_PUTATIVE_ORFAN_CDS	0.41	0.25	0.31	0.21	0.59	0.38	0.46	0.58	0.29	0.56	0.45
KY523104.1_AUL78724.1_MF405918.2_QKU34634.1_GLUTAREDOXIN_CDS	0.17	0.48	0.34	0.06	0.01	0.26	0.29	0.39	0.1	0.28	0.19
KY523104.1_AUL78725.1_MF405918.2_QKU34635.1_CYTIDINE_DEAMINASE_CDS	0.24	0.23	0.37	0.03	0.66	0.34	0.25	0.45	0.16	0.46	0.19
KY523104.1_AUL78726.1_MF405918.2_QKU34636.1_HYPOTHETICAL_PROTEIN_CDS	0.8	0.02	0.26	0.21	0.02	0.29	0.04	0.09	0.27	0.35	0.5
KY523104.1_AUL78727.1_MF405918.2_QKU34637.1_PUTATIVE_ORFAN_CDS	0.27	0.32	0.3	0.12	0.02	0.32	0.41	0.19	0.11	0.42	0.38
KY523104.1_AUL78728.1_MF405918.2_QKU34638.1_PUTATIVE_ORFAN_CDS	0.23	0.12	0.16	0.03	0.01	0.08	0.16	0.02	0.59	0.39	0.21
KY523104.1_AUL78729.1_MF405918.2_QKU34639.1_PUTATIVE_ORFAN_CDS	0.4	0.35	0.34	0.2	0.57	0.36	0.5	0.39	0.34	0.55	0.42
KY523104.1_AUL78730.1_MF405918.2_QKU34640.1_PUTATIVE_ORFAN_CDS	0.3	0.33	0.36	0.18	0.01	0.37	0.43	0.28	0.41	0.43	0.4

KY523104.1_AUL78731.1_MF405918.2_QKU34641.1_HYPOTHETICAL_PROTEIN_CDS	0.38	0.06	0.32	0.4	0.04	0.33	0.35	0.13	0.44	0.47	0.47
KY523104.1_AUL78732.1_MF405918.2_QKU34642.1_MG301_PROTEIN_CDS	0.57	0.44	0.4	0.2	0.12	0.41	0.24	0.47	0.37	0.32	0.45
KY523104.1_AUL78733.1_MF405918.2_QKU34643.1_HYPOTHETICAL_PROTEIN_CDS	0.5	0.42	0.4	0.24	0.11	0.36	0.39	0.38	0.27	0.37	0.45
KY523104.1_AUL78734.1_MF405918.2_QKU34644.1_PUTATIVE_ORFAN_CDS	0.19	0.08	0.35	0	0.1	0.31	0.08	0.27	0.09	0.44	0.02
KY523104.1_AUL78735.1_MF405918.2_QKU34645.1_HYPOTHETICAL_PROTEIN_CDS	0.35	0.26	0.43	0.53	0.16	0.51	0.51	0.47	0.44	0.38	0.39
KY523104.1_AUL78736.1_MF405918.2_QKU34646.1_HYPOTHETICAL_PROTEIN_CDS	0.4	0.64	0.42	0.51	0.05	0.44	0.6	0.18	0.51	0.09	0.44
KY523104.1_AUL78737.1_MF405918.2_QKU34647.1_PUTATIVE_HELICASE_CDS	0.4	0.72	0.45	0.62	0	0.45	0.54	0.42	0.59	0.04	0.23
KY523104.1_AUL78738.1_MF405918.2_QKU34648.1_HELICASE_III/_VV_D5-TYPE_ATPASE_N-TERMINUS_CDS	0.29	0.17	0.33	0.07	0.06	0.31	0.55	0.48	0.29	0.29	0.32
KY523104.1_AUL78739.1_MF405918.2_QKU34649.1_DNA-DEPENDENT_RNA_POLYMERASE_SUBUNIT_RPB9_CDS	0.59	0.67	0.47	0.25	0.63	0.36	0.26	0.25	0.34	0.42	0.47
KY523104.1_AUL78740.1_MF405918.2_QKU34650.1_P87_CDS	0.4	0.22	0.36	0.23	0.27	0.6	0.52	0.36	0.42	0.44	0.45
KY523104.1_AUL78741.1_MF405918.2_QKU34651.1_DNA-DIRECTED_RNA_POLYMERASE_SUBUNIT_6_CDS	0.36	0.64	0.46	0.24	0.03	0.43	0.29	0.21	0.47	0.27	0.2
KY523104.1_AUL78742.1_MF405918.2_QKU34652.1_RNA_LIGASE_2_CDS	0.35	0.28	0.33	0.21	0.01	0.44	0.5	0.26	0.47	0.39	0.44
KY523104.1_AUL78743.1_MF405918.2_QKU34653.1_HYPOTHETICAL_PROTEIN_CDS	0.37	0.34	0.43	0.46	0.17	0.4	0.18	0.23	0.45	0.39	0.42
KY523104.1_AUL78744.1_MF405918.2_AUL80036.2_HYPOTHETICAL_PROTEIN_CDS	0.2	0.17	0.14	0.01	0.01	0.06	0.3	0.02	0.03	0.42	0.13
KY523104.1_AUL77648.1_MF405918.2_QKU33590.1_PUTATIVE_ORFAN_CDS	0.3	0.07	0.3	0.05	0.04	0.22	0.35	0.55	0.13	0.31	0.4
KY523104.1_AUL78450.1_MF405918.2_QKU34371.1_PUTATIVE_ORFAN_CDS	0.02	0	0.02	0	0	0.01	0	0	0	0.11	0.02
KY523104.1_AUL78642.1_MF405918.2_QKU34553.1_STRUCTURAL_PPIASE-LIKE_PROTEIN_CDS	0.02	0.01	0.03	0	0	0.01	0	0	0	0.1	0.02
KY523104.1_AUL78653.1_MF405918.2_QKU34564.1_CYSTEINYL-TRNA_SYNTHETASE_CDS	0	0	0	0	0	0	0	0	0	0.07	0.54
KY523104.1_AUL78506.1_MF405918.2_QKU34423.1_DNA_POLYMERASE_FAMILY_X_CDS	0.11	0.37	0.11	0.01	0	0.07	0.14	0.01	0.01	0.26	0.07
KY523104.1_AUL77901.1_MF405918.2_QKU33841.1_ABC_TRANSPORTER_CDS	0.08	0.2	0.01	0	0	0.01	0	0	0	0.26	0.02
KY523104.1_AUL78328.1_MF405918.2_QKU34255.1_HYPOTHETICAL_PROTEIN_CDS	0.43	0.16	0.3	0.29	0.02	0.31	0.13	0.08	0.46	0.45	0.46
KY523104.1_AUL78612.1_MF405918.2_QKU34523.1_PUTATIVE_ORFAN_CDS	0.08	0.07	0.08	0	0	0.03	0.09	0.01	0.03	0.21	0.07
KY523104.1_AUL78469.1_MF405918.2_QKU34388.1_PUTATIVE_ORFAN_CDS	0.32	0.62	0.44	0.1	0.01	0.41	0.51	0.35	0.41	0.18	0.21
KY523104.1_AUL78343.1_MF405918.2_QKU34270.1_DUAL_SPECIFICITY_PHOSPHATASE_CDS	0.31	0.1	0.28	0.03	0.01	0.22	0.04	0.15	0.05	0.36	0.06
KY523104.1_AUL78433.1_MF405918.2_QKU34354.1_PUTATIVE_ORFAN_CDS	0.31	0.12	0.29	0.09	0.01	0.32	0.57	0.13	0.4	0.28	0.38
KY523104.1_AUL77484.1_MF405918.2_QKU33434.1_HYPOTHETICAL_PROTEIN_CDS	0	0.01	0.01	0	0	0.01	0.03	0	0.01	0.14	0.02
KY523104.1_AUL78636.1_MF405918.2_QKU34547.1_PUTATIVE_ORFAN_CDS	0.42	0.04	0.21	0.09	0.05	0.27	0.6	0.05	0.48	0.22	0.51
KY523104.1_AUL78611.1_MF405918.2_QKU34522.1_PUTATIVE_ORFAN_CDS	0.01	0	0.11	0	0	0.02	0	0.6	0.01	0.13	0.02
KY523104.1_AUL77485.1_MF405918.2_QKU33435.1_MG971_PROTEIN_CDS	0.01	0.01	0.01	0	0	0.02	0	0.01	0.01	0.08	0.02
KY523104.1_AUL77668.1_MF405918.2_QKU33609.1_NTP_PYROPHOSPHOHYDROLASE_CDS	0.01	0.04	0.03	0	0	0.02	0	0	0	0.36	0.04
KY523104.1_AUL78081.1_MF405918.2_QKU34016.1_HYPOTHETICAL_PROTEIN_CDS	0.04	0.02	0.04	0	0	0.02	0.11	0.01	0.02	0.1	0.03
KY523104.1_AUL78578.1_MF405918.2_QKU34492.1_PUTATIVE_ORFAN_CDS	0.39	0.4	0.49	0.89	0.52	0.46	0.21	0.38	0.31	0.39	0.42
KY523104.1_AUL77685.1_MF405918.2_QKU33626.1_ANKYRIN_REPEAT_PROTEIN_CDS	0.56	0.09	0.03	0	0	0.02	0.21	0	0.01	0.13	0.56
KY523104.1_AUL78109.1_MF405918.2_QKU34043.1_DNA_TOPOISOMERASE_1B_CDS	0.03	0.1	0.01	0	0	0.01	0	0	0	0.26	0
KY523104.1_AUL78705.1_MF405918.2_QKU34615.1_AAEL017480-PA_CDS	0.17	0.01	0.01	0	0	0.01	0.02	0	0	0.15	0.05
KY523104.1_AUL77669.1_MF405918.2_QKU33610.1_PUTATIVE_ORFAN_CDS	0.27	0.02	0.05	0	0	0.05	0	0.01	0	0.22	0.1
KY523104.1_AUL78342.1_MF405918.2_QKU34269.1_PUTATIVE_ORFAN_CDS	0.31	0.1	0.28	0.03	0.01	0.22	0.04	0.15	0.05	0.36	0.06
KY523104.1_AUL77961.1_MF405918.2_QKU33899.1_HYPOTHETICAL_PROTEIN_CDS	0.02	0.01	0.05	0	0	0.03	0.04	0	0	0.17	0
KY523104.1_AUL78010.1_MF405918.2_QKU33946.1_HYPOTHETICAL_PROTEIN_CDS	0.04	0.02	0.01	0	0	0	0	0	0.01	0.15	0.74
KY523104.1_AUL78011.1_MF405918.2_QKU33947.1_GLYCOSYL_TRANSFERASE_CDS	0.05	0.03	0.04	0	0	0.02	0.12	0	0	0.16	0.05
KY523104.1_AUL77512.1_MF405918.2_QKU33460.1_MACROCIN_O-METHYLTRANSFERASE_CDS	0.2	0.02	0.01	0	0	0.01	0	0	0.02	0.26	0.61
KY523104.1_AUL78210.1_MF405918.2_QKU34139.1_PUTATIVE_ORFAN_CDS	0.03	0.01	0.08	0	0	0.05	0.52	0.02	0.04	0.28	0

KY523104.1_AUL78601.1_MF405918.2_QKU34513.1_HYPOTHETICAL_PROTEIN_CDS	0.06	0.05	0.04	0	0	0.02	0.05	0	0	0.14	0.01
KY523104.1_AUL77684.1_MF405918.2_QKU33625.1_HYPOTHETICAL_PROTEIN_CDS	0.04	0.02	0.02	0	0	0.02	0.23	0.01	0.06	0.09	0.07
KY523104.1_AUL78706.1_MF405918.2_QKU34616.1_HYPOTHETICAL_PROTEIN_CDS	0.15	0.01	0.03	0	0	0.02	0	0	0	0.15	0.02
KY523104.1_AUL77513.1_MF405918.2_QKU33461.1_MG19_PROTEIN_CDS	0.64	0.05	0.03	0	0	0.03	0	0	0	0.12	0.32
KY523104.1_AUL78334.1_MF405918.2_QKU34261.1_PUTATIVE_ORFAN_CDS	0.21	0.12	0.32	0.02	0	0.5	0.25	0.29	0.5	0.38	0.08
KY523104.1_AUL77537.1_MF405918.2_QKU33485.1_PUTATIVE_ORFAN_CDS	0.17	0.47	0.32	0.05	0.02	0.27	0.38	0.52	0.13	0.24	0.23
KY523104.1_AUL77525.1_MF405918.2_QKU33473.1_PUTATIVE_ORFAN_CDS	0	0	0.03	0	0	0.02	0	0	0	0.11	0
KY523104.1_AUL78080.1_MF405918.2_QKU34015.1_HYPOTHETICAL_PROTEIN_CDS	0.15	0.12	0.05	0	0.03	0.05	0.3	0.08	0.1	0.13	0.02
KY523104.1_AUL78003.1_MF405918.2_QKU33939.1_HYPOTHETICAL_PROTEIN_CDS	0.01	0	0.03	0	0	0.01	0	0	0	0.12	0.02
KY523104.1_AUL78652.1_MF405918.2_QKU34563.1_PUTATIVE_ORFAN_CDS	0.01	0.02	0.04	0	0	0.02	0	0	0	0.16	0.04
KY523104.1_AUL78315.1_MF405918.2_QKU34242.1_PUTATIVE_ORFAN_CDS	0.7	0.06	0.31	0.2	0	0.29	0.22	0.19	0.15	0.32	0.51
KY523104.1_AUL77900.1_MF405918.2_QKU33840.1_PUTATIVE_ORFAN_CDS	0.02	0.01	0.01	0	0	0.01	0	0	0	0.16	0.27
KY523104.1_AUL78108.1_MF405918.2_QKU34042.1_HYPOTHETICAL_PROTEIN_CDS	0.25	0.01	0.05	0	0	0.03	0	0	0	0.15	0.35
KY523104.1_AUL77919.1_MF405918.2_QKU33859.1_PUTATIVE_ORFAN_CDS	0.24	0.35	0.33	0.06	0.64	0.32	0.17	0.22	0.19	0.5	0.21
KY523104.1_AUL78168.1_MF405918.2_QKU34101.1_HISTIDINE_PROTEIN_KINASE_SLN1_CDS	0.33	0.24	0.33	0.01	0.02	0.3	0.37	0.19	0.2	0.34	0.28
KY523104.1_AUL77965.1_MF405918.2_QKU33903.1_HYPOTHETICAL_PROTEIN_CDS	0.3	0.62	0.72	0.27	0.03	0.74	0.36	0.55	0.52	0.38	0.05
